# Identification of the vascular plants of Churchill, Manitoba, using a DNA barcode library

**DOI:** 10.1186/1472-6785-12-25

**Published:** 2012-11-28

**Authors:** Maria L Kuzmina, Karen L Johnson, Hannah R Barron, Paul DN Hebert

**Affiliations:** 1 Biodiversity Institute of Ontario, University of Guelph, Guelph, ON, Canada; 2 The Manitoba Museum, Botany Department, Manitoba, Canada; 3 Trent University, Environmental and Life Sciences, Peterborough, ON, Canada

**Keywords:** Arctic, DNA barcoding, *rbc*L, *mat*K, ITS2, Species resolution, Climate change, Biomonitoring

## Abstract

**Background:**

Because arctic plant communities are highly vulnerable to climate change, shifts in their composition require rapid, accurate identifications, often for specimens that lack diagnostic floral characters. The present study examines the role that DNA barcoding can play in aiding floristic evaluations in the arctic by testing the effectiveness of the core plant barcode regions (*rbc*L, *mat*K) and a supplemental ribosomal DNA (ITS2) marker for a well-studied flora near Churchill, Manitoba.

**Results:**

This investigation examined 900 specimens representing 312 of the 354 species of vascular plants known from Churchill. Sequencing success was high for *rbc*L: 95% for fresh specimens and 85% for herbarium samples (mean age 20 years). ITS2 worked equally well for the fresh and herbarium material (89% and 88%). However, sequencing success was lower for *mat*K, despite two rounds of PCR amplification, which reflected less effective primer binding and sensitivity to the DNA degradation (76% of fresh, 45% of herbaria samples). A species was considered as taxonomically resolved if its members showed at least one diagnostic difference from any other taxon in the study and formed a monophyletic clade. The highest species resolution (69%) was obtained by combining information from all three genes. The joint sequence information for *rbc*L and *mat*K distinguished 54% of 286 species, while *rbc*L and ITS2 distinguished 63% of 285 species. Discrimination of species within *Salix*, which constituted 8% of the flora, was particularly problematic. Despite incomplete resolution, the barcode results revealed 22 misidentified herbarium specimens, and enabled the identification of field specimens which were otherwise too immature to identify. Although seven cases of ITS2 paralogy were noted in the families Cyperaceae, Juncaceae and Juncaginaceae, this intergenic spacer played an important role in resolving congeneric plant species at Churchill.

**Conclusions:**

Our results provided fast and cost-effective solution to create a comprehensive, effective DNA barcode reference library for a local flora.

## Background

Climate change has already led to substantial modification in the composition of Arctic plant communities [1] as reflected by shifting ranges and genetic differentiation [2]. Many arctic plant species are likely to lose genetic diversity due to their limited dispersal capacity, and consequent range reduction [3], making them particularly vulnerable to climate change. Identifying the impacts of climate change on the composition of plant communities is currently the focus of many studies in the Arctic which employ two main approaches. The first examines the impact of manipulations in light, temperature and nutrient regimes on species composition and richness [4-7]. The second approach involves direct examination of plant community composition to identify species that are particularly effective predictors of shifts in vegetation in response to climate change [8]. Both approaches require rapid and accurate identification of plants, many of which lack diagnostic floral or fruit characters at the time of their collection.

DNA barcoding employs sequence diversity in short, standardized gene region(s) to facilitate species identification [9]. Two gene regions from the chloroplast genome, *rbc*L and *mat*K, have been adopted as the standard barcodes for land plants [10]. Both of these genes have played a very important role in phylogenetic reconstructions for land plants due to their strong phylogenetic signal [11-14]. Their capacity to resolve species in local floras has now been tested in many settings, particularly in species-rich tropical communities. As well, numerous studies have tested the additional discrimination provided by supplemental chloroplast (*trn*H*-psb*A*, atp*B*-atp*H*, rpo*C1) and nuclear (ITS) markers [15-19]. All prior studies have reported 100% success in generic-level assignments, while success in species-level assignment has ranged from 50 – 92% for the two-locus barcode (*rbc*L &*mat*K), and from 70 – 98% with one or more supplementary markers. However, in all analyzed cases DNA barcoding has proven an efficient approach for the evaluation of hyper-diverse floras.

The nuclear ribosomal DNA region ITS and its two components, ITS1 and ITS2, have been extensively utilized for studies on the molecular systematics of plants because of their high rate of nucleotide substitution and relative ease of amplification, sequencing and alignment [20]. Among the varied supplemental barcode markers, ITS2 shows particular promise because its short length (160-320 bp) and the availability of universal primers make it easy to recover. Although it has been suggested that ITS2 exhibits too much paralogy, and is too susceptible to fungal contamination to be adopted as a DNA barcode marker [21,22], it delivered 92.7% discrimination in a recent study on 4800 species of medicinal plants [17]. Given this high performance, ITS2 merits serious consideration as a standard marker for plant barcoding.

Although plant communities in temperate and arctic regions are much less diverse than those in the tropics [23], they may not be easier targets for DNA barcode analysis because rates of molecular evolution in both plastid and nuclear genomes appear lower in groups of flowering plants with low diversity [24] and in plant species from high latitudes [25]. However, there is some evidence that arctic plant communities have experienced more rapid speciation, due to intense processes of hybridization, refugial isolation and range shifts [26]. The question of how this affects the performance of DNA barcoding for the identification of plant species has seen little investigation. However, a recent study of the flora at a temperate site in Canada revealed 93% success in species identification with *rbc*L &*mat*K, while the addition of the *trn*H*-psb*A intergenic spacer raised resolution to 95% [27].

The present study tests the effectiveness of DNA barcoding for the identification of species in the flora at Churchill, Manitoba, Canada. Our decision to work at this locality reflects an ongoing effort to assemble a comprehensive DNA barcode library for all animal and plant species at Churchill. Sequence information was collected for three gene regions (*rbc*L, *mat*K, ITS2) from 312 of the 354 species of vascular plants known from this locale [28,29]. Since herbarium collections can aid the rapid creation of comprehensive DNA barcode libraries [30], we compared the success of barcode recovery from herbarium and freshly collected specimens preserved in silica gel. We also investigated factors affecting sequence recovery for these three gene regions in a high-throughput barcoding setting, and adjusted protocols to enhance success. Finally, we compared the success of species identification in this arctic flora with those reported for temperate and tropical floras.

## Methods

### Study area

The Churchill area lies within the Hudson Bay Lowlands in a region where quartzite and dolomite bedrock has created a wide range of microhabitats. Poor drainage has led to the formation of extensive peat bogs, while broad tidal flats lie along the margin of Hudson Bay. Churchill is positioned in southern hypoarctic tundra with elements of high boreal subzone following along the Churchill River [31], with stable coexistence of oceanic and continental floristic elements [32]. The community of vascular plants around Churchill has been re-established in the 8000 years since deglaciation [29,33,34].

### Tissue collection and identification

We examined 900 specimens including representatives of 312 species, 147 genera, 51 families, and 24 orders. Plant tissue from 540 specimens (60% of total) was collected from 35 localities around Churchill in July 2009, and dried in silica gel at room temperature. The remaining 360 specimens derived from the University of Manitoba Herbarium (WIN) and the Manitoba Museum’s Botany Department (MMMN) and had a mean age of 20 years. When available, several individuals (2-5) per species from different populations were analyzed. Freshly collected specimens were identified using standard taxonomic references [35-38], with subsequent confirmation through comparison with specimens in WIN, MMMN, and CAN. The identification of willows (*Salix*) was confirmed by George Argus (CAN). Vouchers for the 540 freshly collected specimens, representing 241 species have been deposited in the BIO Herbarium (OAC) at the University of Guelph with duplicates at the Churchill Northern Studies Centre. The sequences for three barcode markers: *rbc*L, *mat*K, and ITS2 are publicly accessible in the project entitled “Plants of Churchill 2009” on BOLD [39], and are also available on GenBank under the accession numbers shown in Additional file 1.

### Botanical nomenclature

We adopted the Checklist of the Panarctic Flora (PAF) Vascular Plants [40] to alleviate problems created by the frequent lack of standardization in name usage for species with Holarctic distributions. Some generic names diverge from those in the most recent taxonomic treatments for the North America flora [35-38,41] where *Oxycoccus* is placed within *Vaccinium*[42]*, Cyrtorhyncha* within *Ranunculus*[43]*,* two species of *Chamerion* are assigned to *Epilobium*, and *Comarum palustre* is treated as *Potentilla palustris*[36]. However, some new assignments (*Arctous*, *Orthilia*) have been accepted in both the Flora of North America, and in PAF [40,41]. According to the PAF checklist, 87 of 147 genera in our study were represented by a single species. Family and ordinal assignments follow the Angiosperm Phylogeny Group III [14].

### DNA extraction, PCR and sequencing

DNA extraction followed standard protocols at the Canadian Centre for DNA barcoding (CCDB) for plants [44]. In brief, small amounts of dry plant tissue (0.5 cm^2^) were placed into racked sterile mini tube strips. A 3.17 mm stainless steel bead was added to each tube before it was closed with a sterile cap strip. The tissue was then ground into fine powder using a Tissue Lyser (Qiagen, USA) with rack adapters at 28 Hz for 30 seconds; the adaptor was then rotated, and one more round of grinding was applied. The powdered tissues were incubated with 2x CTAB buffer at 65°C for 1 hour and DNA was then extracted using semi-automated method employing glass fiber filtration [45,46]. The final concentration of the eluted DNA was 20-40 ng/μL.

Three gene regions (*rbc*L*, mat*K, ITS2) were amplified using the CCDB plant protocol [46,47] with Platinum ® Taq DNA polymerase (Invitrogen), and pre-made frozen plates [48]. Different PCR conditions were employed for *mat*K than for the other two regions (Table 1). Strong amplification of *rbc*L and ITS2 was obtained with low concentrations of primers (0.1 μM), dNTPs (0.05 mM), and Taq polymerase (0.024 U/μL). Subsequent 5-10x dilution of the amplicons enabled direct sequencing without PCR purification. One primer set (rbcLa-F [49] and rbcLa-R [16]) was used for all *rbc*L analysis and another for all ITS2 analysis (ITS-S2F [17], and ITS4 [50]). The *mat*K region required higher concentrations of all reagents: primers (0.5 μM), dNTPs (0.2 mM), and Taq polymerase (0.1 U/μL) to optimize amplicon recovery. A 10-fold dilution of DNA (2-4 ng/μL), and a smaller reaction volume (7.5 μL) improved the quality of amplicons for *mat*K, while reducing costs. We employed two primer sets to aid the recovery of *mat*K. The first round was performed with matK-1RKIM-f and matK-3FKIM-r [pers.com. Ki-Joong Kim]. Failed samples were assembled into a new plate and amplified with matK_390f and matK_1326r [51]. Primer sequences for *rbc*L, *mat*K, and ITS2 are available on the CCDB Protocols website [52] and in Table 1. The cycle sequencing reaction and subsequent clean-up employed standard CCDB protocols [53] with products analyzed on an ABI 3730xl capillary sequencer. 

**Table 1:** Primers and PCR protocols

		
**PCR recipe for *rbc *L and ITS2 (total volume of the reaction: 12.5 μL)**		
**Reagents**	**Final concentration**	**Volume per reaction (μL)**
**10%trehalose**	5%	6.25
**ddH20**		2.00
**10X buffer**	1x	1.25
**50 mM MgCl2**	2.5 mM	0.625
**10 μM primer F**	0.1	0.125
**10 μM primer R**	0.1	0.125
**10 mM dNTPs**	0.05	0.0625
**Polymerase (5 U/μl)**	0.024 U/μL	0.06
**TOTAL**		10.50
**DNA template (20-40 ng/μL)**		2 .00
**PCR recipe for *mat*K (total volume of the reaction: 7.5 μL)**		
**Reagents**	**Final concentration**	**Volume per reaction(μL)**
**20**% **trehalose**	5%	1.875
**ddH20**		2.60
**10X buffer**	1x	0.75
**50 mM MgCl2**	1.5 mM	0.225
**10 μM primer F**	0.5	0.375
**10 μM primer R**	0.5	0.375
**10 mM dNTPs**	0.2	0.15
**Polymerase (5 U/μl)**	0.1 U/μL	0.15
**TOTAL**		6.50
**DNA template (2-4 ng/μL)**		1.00
**Primer sets**		
	**Sequence**	**Reference**
***rbc*L primers**		
rbcLa-F	ATGTCACCACAAACAGAGACTAAAGC	[49] Levin et al. 2003
rbcLa-R	GTAAAATCAAGTCCACCRCG	[16] Kress & Erickson, 2009
***mat*K primers**		
MatK-1RKIM-f	ACCCAGTCCATCTGGAAATCTTGGTTC	Ki-Joong Kim, pers. comm.
MatK-3FKIM-r	CGTACAGTACTTTTGTGTTTACGAG	Ki-Joong Kim, pers. comm.
MatK_390f	CGATCTATTCATTCAATATTTC	[51] Cuenoud et al. 2002
MatK_1326r	TCTAGCACACGAAAGTCGAAGT	[51] Cuenoud et al. 2002
**ITS2 primers**		
ITS2-S2F	ATGCGATACTTGGTGTGAAT	[17] Chen et al. 2010
ITS4	TCCTCCGCTTATTGATATGC	[50] White et al. 1990

### Sequence data analysis

Sequence chromatograms were edited using CodonCode Aligner v.3.7.1 (CodonCode Co, USA). The traces were assembled into bidirectional contigs, primer sequences were removed, and all ambiguous base calls were checked manually. Contigs were compared using the MUSCLE multiple sequence alignment algorithm [54] implemented in CodonCode Aligner. The preliminary alignment with MUSCLE facilitated both the identification and correction of sequencing errors, mostly involving indels in homopolymer regions. BLAST was employed to recognize and exclude any fungal/algal sequences among ITS2 amplicons. The fasta sequences were visualized in BioEdit [55] and MEGA 5 [56], and double-checked for editing errors. A final alignment for the *rbc*L and *mat*K sequences was generated using back-translation in transAlign [57]. This step was particularly important to create the most parsimonious translated alignment for *matK* due to its multiple indels when taxonomically distant groups were compared. TransAlign also helped to reveal ORF shifts caused by editing errors (single missing or extra nucleotides), or pseudogenes. Sequences for ITS2 were clustered into 17 groups composed of species in a single or closely related group of orders. The sequences in each cluster were then aligned with BioEdit using CLUSTAL with a gap penalty of 5 for both pairwise and multiple alignments. An ITS2 sequence was considered as paralogous if it was assigned to a clade (or clades) that conflicted with the species assignment based on chloroplast marker(s) and morphology. The total alignments for *rbc*L, *mat*K and ITS2 alignments were concatenated into a profiled alignment with SequenceMatrix 1.5 alpha9 [58].

Data management and calculation of mean pairwise distances (MPD,%) were performed in BOLD [39]. The standard deviation (σ) was calculated to estimate dispersion of this parameter. The correlation coefficient (r) between three pairs of markers was calculated using MPD values for the families as covariates. Significance test (p-value) was done by Z-score method. All specimens with sequence data for ITS2 and for at least one of the two chloroplast markers (*rbc*L, *mat*K) were included in three datasets (*rbc*L &*mat*K; *rbc*L & ITS2; *rbc*L, *mat*K & ITS2). Analysis was performed using Maximum parsimony (MP) to establish if a particular taxon formed a monophyletic clade. Prior to analysis, fasta files were converted into Nexus format using the Nexus Class Library [59]. PAUP was then used to implement Parsimony analysis. Support for branching patterns was assessed using the parsimony ratchet [60]. Consensus tree was generated from a set of the most parsimonious trees, and a single tree for each dataset was visualized in iTOL [61,62]. The species resolution for the supermatrix based on the three markers was compared with those for matrices based on two markers (*rbc*L &*mat*K*; rbc*L & ITS2). Congeneric species were considered as resolved when individuals within one species showed at least one consistent diagnostic difference from other species and produced a monophyletic clade in the MP tree. Since some genera were represented by a single species, their percent of species resolution, which actually reflects generic resolution, was calculated separately from the genera with more than one species. This approach helped to indicate sensitivity of different combinations of markers to species resolution within genera.

## Results and discussion

### Sequencing success

**Figure 1:**
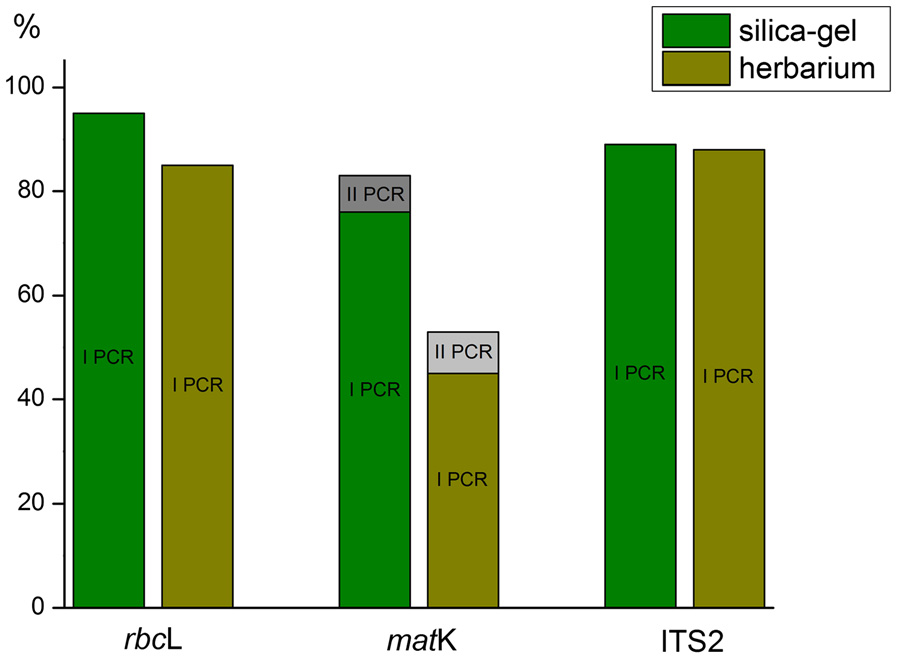
**Sequencing success (%) for tissue samples preserved in silica-gel and collected from herbarium specimens (mean age 20 years).** Green: plant tissue preserved in silica-gel. Olive: plant tissue collected from herbarium specimens. *rbc*L: one round of PCR with the primers rbcLa-F/rbcLa-R. *mat*K: two consequent rounds of PCR with the primers matK-1RKIM-f/matK-3FKIM-r and matK_390f/matK_1326r (for the DNA samples failed in the first round). ITS2: one round of PCR with the primers ITS-S2F/ITS4.

ITS2 sequences were obtained with equal success from fresh and herbarium material (89%, and 88% respectively), while sequence recovery for *rbc*L was 10% lower for herbarium than fresh specimens (85% versus 95%). The first set of *mat*K primers showed 36% less success from herbarium than fresh specimens (45% versus 76%). The second set of PCR primers delivered *mat*K records for another 7% of the fresh specimens, producing an overall 83% success, while 8% new records were obtained from herbarium samples, raising their overall success to 53% (Figure 1, Additional file 1**). These results indicate a strong correlation between recovery of the markers and their length (circa 800 bp for *mat*K, 552 bp for *rbc*L, and circa 350 bp for ITS2), reflecting DNA degradation in the herbarium specimens. However, the low success in sequence recovery for *mat*K also reflected difficulties in primer binding as evidenced by the 19% lower success in its recovery from fresh specimens in comparison with rbcL. The second round of PCR for *mat*K did amplify some groups that initially failed, but the overall recovery still fell well below those for the other two markers. 

**Figure 2:**
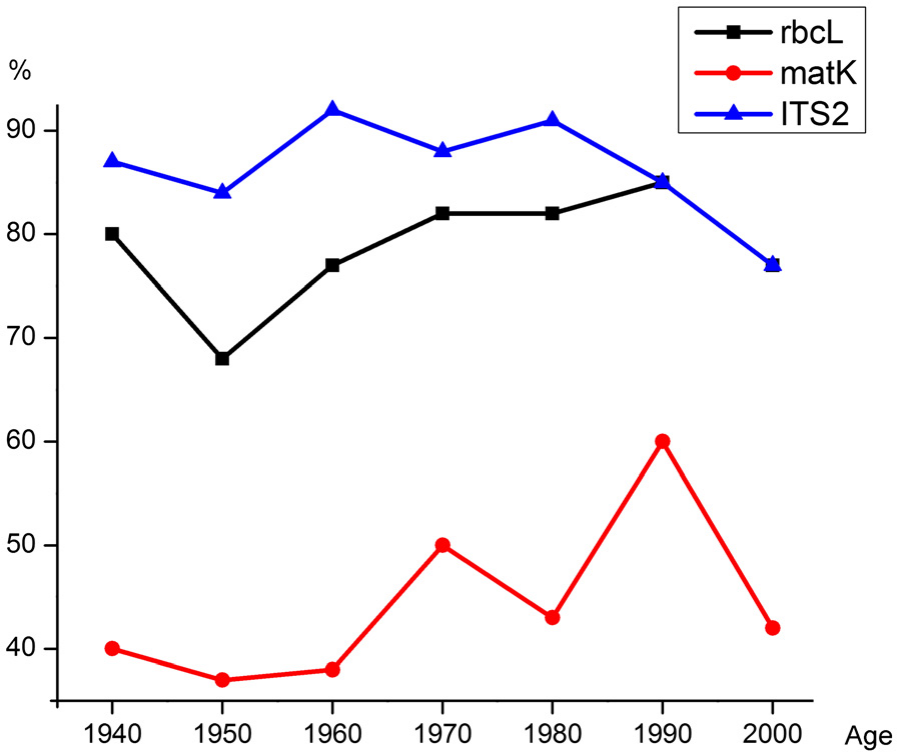
**Sequencing success (%) versus age of the herbarium samples.** Black: *rbc*L. Red: *mat*K. Blue: ITS2.

There was no noticeable association between the age of herbarium specimens and sequence recovery (Figure 2). ITS2 showed a high, stable recovery rate across all ages. The *rbc*L barcode also demonstrated good recovery that was not strongly impacted by age of the herbarium samples. Although herbarium specimens had lower recovery for *mat*K, their age did not markedly affect success. However, sequencing success was substantially lower for all three genes in genera *(Plantago, Atriplex, Anemone, Amerorchis)* with relatively thick leaves, likely due to slower desiccation of their tissues and consequent DNA degradation*.* This observation confirms previous evidence that the quality of herbarium preservation plays a more important role for sequence recovery than age of the samples [63]. Most of the herbarium specimens that we examined generated barcode sequences, revealing that herbaria can both accelerate the development of comprehensive barcode reference libraries and allow this work to be completed cost-effectively. Additionally, the analysis of herbarium vouchers and dialogue with curators increased the reliability of the identifications for the specimens that we analyzed. There was a reciprocal benefit for the collections, as barcode screening revealed inconsistencies in identification that provoked reconsideration of initial identifications. 

Our work revealed several sources of interpretational complexity. No cases of ITS2 contamination were detected in the freshly collected specimens, but about 1% of the sequences from herbarium samples were of fungal or algal origin. Several cases of apparent sampling errors were also detected in herbarium specimens of graminoids (Poaceae, Juncaceae, and Cyperaceae) which may reflect mixed samples. Therefore, it is critical to record the exact site on the herbarium sheet where the tissue sample is obtained. Finally, the second round of PCR amplified *mat*K pseudogenes in four species (*Amerorchis rotundifolia, Limnorchis aquilonis, Limnorchis huronensis*, *Salix myrtillifolia* - Additional file 2). All sequences reflecting contamination or pseudogenes were excluded from further analysis.

Success in sequence recovery ranged from a low of 72% (*matK*) to a high of 90% (*rbc*L), with 88% success for ITS2. Twenty nine specimens failed to provide a sequence for both plastid markers, creating 871 specimens with sequence records for both ITS2 and one of these genes (*rbc*L &*mat*K from 68%, *rbc*L & ITS2 from 82%). Information for all three genes was available for 63% of the samples. In total 286 of the 312 species (92%) that we examined had sequence data for either *rbc*L or *mat*K allowing their inclusion in our evaluation of species resolution.

### Within-family distances

**Figure 3:**
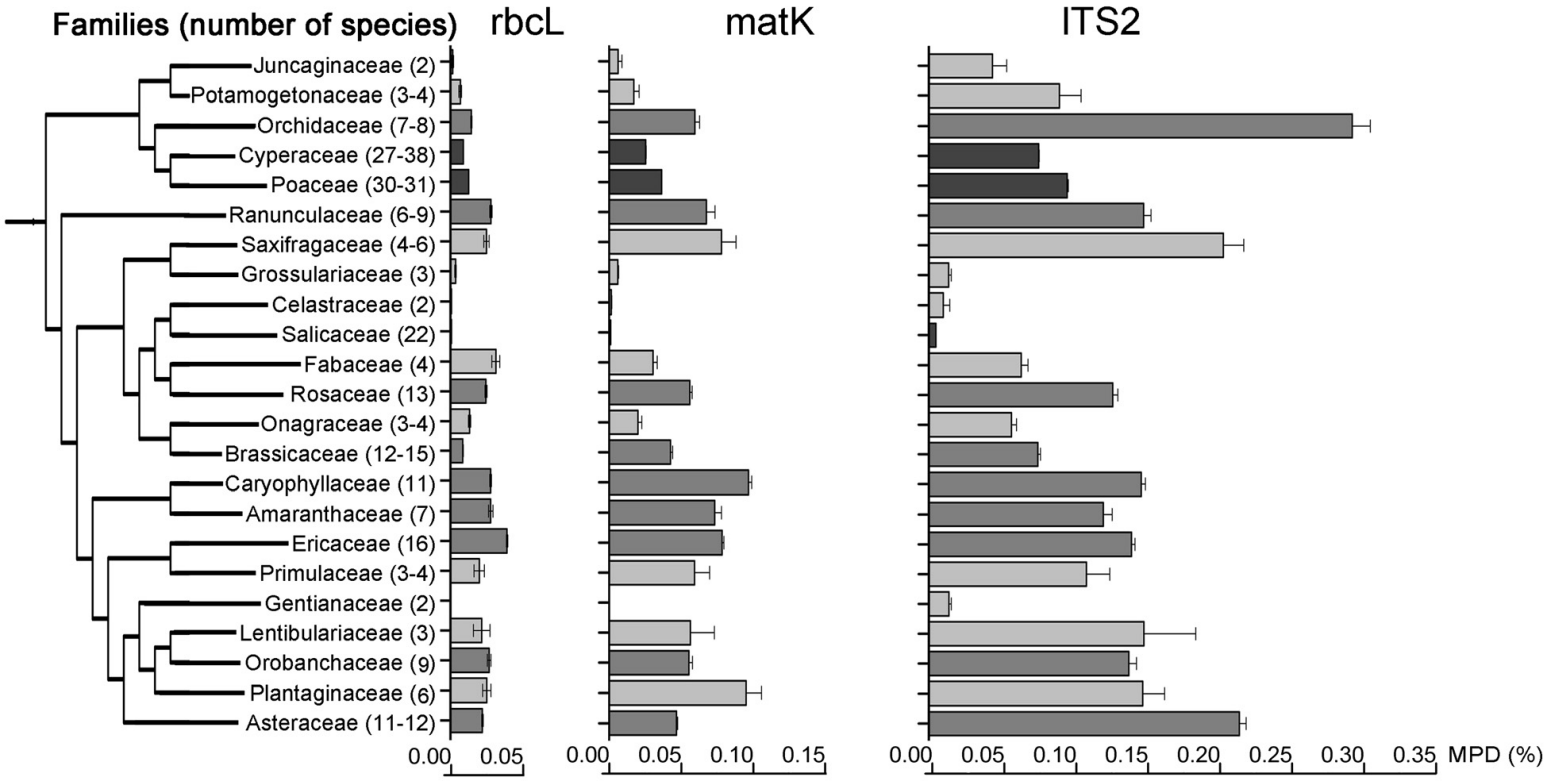
**Within families mean pairwise distance (MPD) analysis for *rbc *L, *mat *K, and ITS2.** Families with two and more species with available barcodes have been selected. Families are displayed in phylogenetic order, with tree constructed using Phylomatic [72]. The number of species is shown in round brackets next to the family name. In some cases this number slightly varied depending on the available barcodes for three different markers. Light grey: 2-6 species. Grey: 7-16 species. Dark grey: 22-38 species. The bars show means for the pairwise distances (%) between all specimens within the families with available barcodes for the markers *rbc*L (average 0.02%), *mat*K (average 0.04%), ITS2 (average 0.12%). However, the correlation of MPD values is examined between two chloroplast markers (*rbc*L and *mat*K), as well as between two chloroplast (*rbc*L and *mat*K) and a nuclear marker (ITS2). The error bars indicate standard deviations (σ) from mean.

Although mean pairwise distances (MPD) did not show a tight correlation with the resolution capacity of each marker, the comparison of this parameter within families represented by more than one species did reveal differences in the substitution rate among the three markers, and among taxonomic groups (Figure 3). The mean MPD for *rbc*L (0.017%) was noticeably lower than those for *mat*K (0.04%), and ITS2 (0.12%). The MPD for each marker also varied significantly among families, with a strong positive correlation between the two chloroplast markers (*rbc*L/*mat*K: r=0.84; p<0.05) as well as between the chloroplast and nuclear markers (*rbc*L/ITS2: r=0.60; *mat*K/ITS2: r=0.75; p<0.05), confirming prior evidence for a positive relationship between rates of molecular evolution in the chloroplast and nuclear genomes [20]. For example, families (Ranunculaceae, Saxifragaceae, Ericaceae, Caryophyllaceae, Amaranthaceae, Plantaginaceae) with high variation in *rbc*L and *mat*K also showed high sequence variation in ITS2. Conversely, other families (Juncaginaceae, Grossulariaceae, Celastraceae, Salicaceae, Gentianaceae) showed very low genetic variation in all three markers. The Brassicaceae was exceptional as its members showed relatively high sequence variation for *mat*K, and ITS2 despite low divergence for *rbc*L. Six other families (Orchidaceae, Poaceae, Cyperaceae, Rosaceae, Lentibulariaceae, Asteraceae) showed markedly higher variation for ITS2 than for the chloroplast markers, indicating the potential importance of this gene region for discriminating closely related species within these families. 

The MPD value for a family was not linked to the number of species within it. For example, the most speciose families in this study (Cyperaceae, Poaceae) had moderate or low variation for all three markers. Moreover, although *Salix* was the most diverse genus (22 species) in the flora at Churchill, it showed some of the lowest MPD scores for all three genes. This observation supports prior evidence for differing rates of molecular evolution among plant families [64], and reveals that the most diverse plant families in the arctic do not have the highest rates of molecular evolution, a pattern which differs from the positive relationship between species number and rates of molecular evolution in flowering plants at the global scale [24].

### Intraspecific variation and ITS2 paralogy

Intraspecific variation was not observed for *rbc*L in any of the 205 species with two or more sequence records. However, intraspecific variation was detected in 18 of 156 species with multiple records for *mat*K and in 35 of 189 species with multiple records for ITS2. Twelve of these species demonstrated intraspecific variation for both markers, six only showed intraspecific variation for *mat*K, while twenty three showed intraspecific variation for ITS2 (Additional file 3).

Paralogous sequences were detected in 7 of the 189 species with multiple records for ITS2. Some of these cases involved assignments to closely related species (e.g. *Triglochin palustris, Luzula groenlandica, Carex rotundata, Carex saxatilis)*, a pattern which might reflect hybridisation events. However, in other cases (*Carex capillaris, Carex concinna, Kobresia myosuroides*), the ITS variants formed independent, distant clades within the same family, while the corresponding chloroplast markers assigned these individuals to monophyletic clades, excluding DNA contamination as a source of the paralogy.

### Species resolution with different barcode markers

**Figure 4:**
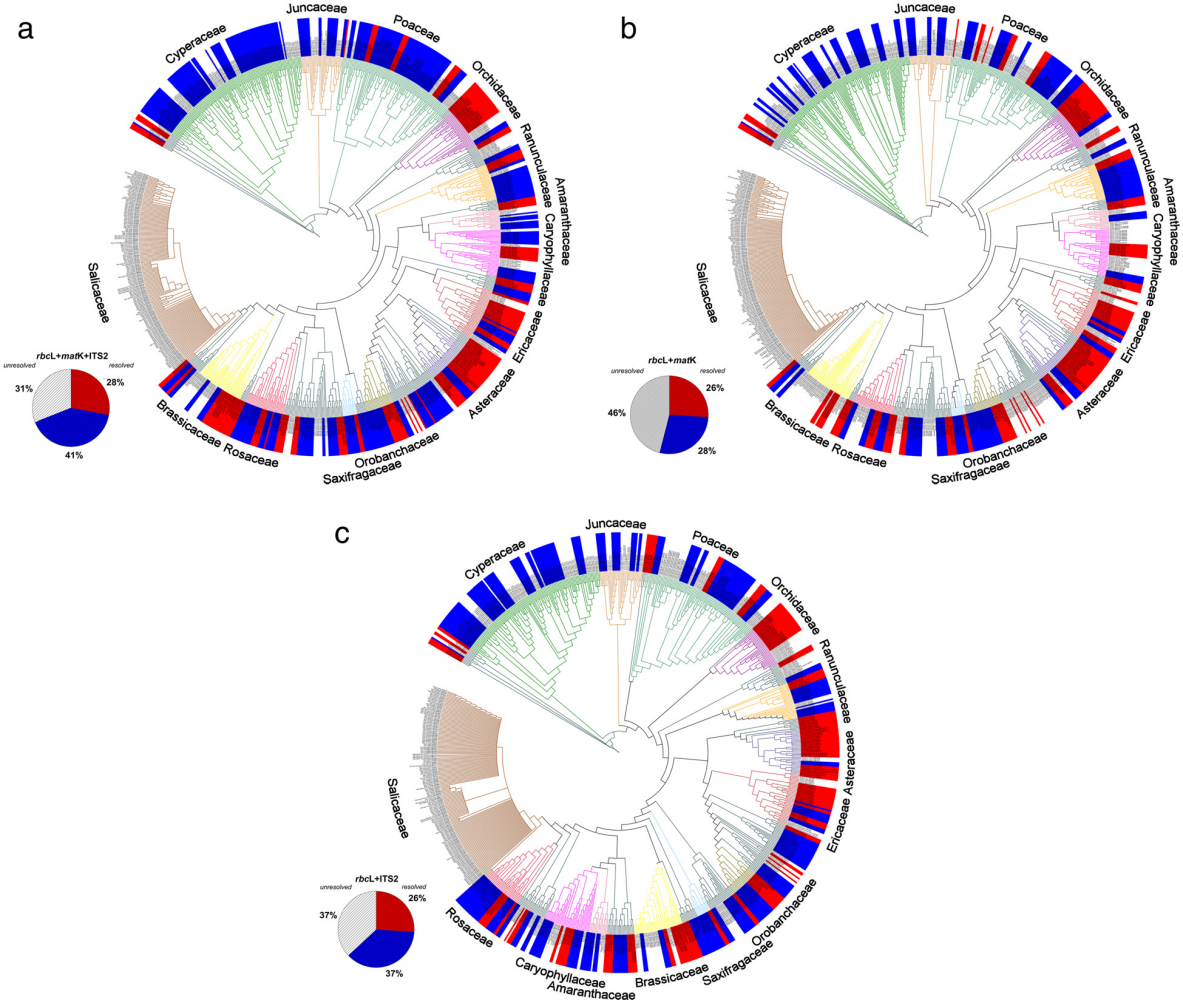
**Species resolution with combined data for *rbc*L, *mat *K and ITS2 (consensus trees from MP-ratchet analysis).** Red: genera with a single species. Blue: genera with more than one species. **a**) *rbc*L, *mat*K & ITS2: 28% species resolution for the genera with a single species, and 41% for the genera with more than one species. **b**) *rbc*L &*mat*K: 26% species resolution for the genera with a single species, and 28% for the genera with more than one species. **c**) *rbc*L & ITS2: 26% species resolution for the genera with a single species, and 37% for the genera with more than one species.

The discriminatory power of the three barcode regions was considered in both pairwise and in the sole three-way combination. The consensus maximum parsimony (MP) tree based on all three markers (Figure 4a) was compared with those for two markers rbcL & matK (Figure 4b), and rbcL & ITS2 (Figure 4c). Because 28% of the species in the analysis belong to genera with a single species at Churchill, their resolution is effectively at a generic level. These taxa are indicated by red labels in the tree diagrams, while species in polytypic genera are represented in blue. The percent of resolved species from monotypic genera was included in overall calculation of the species resolution, but the percent of the species resolution for congeners is provided separately to demonstrate sensitivity of this parameter to the selection of markers. As expected, the combination of three markers (*rbc*L, *mat*K, & ITS2) delivered both the highest overall species resolution (69%) and the highest resolution of congeneric species (41%). By contrast, the tree built with the two standard barcodes (*rbc*L &*mat*K) showed 54% overall resolution, and just 28% resolution of congeners. Interestingly, the combination of *rbc*L & ITS2 delivered 63% overall species resolution and 37% resolution for the species in polytypic genera. Despite the incomplete resolution, the DNA barcode data revealed that 22 of 333 herbarium specimens were misidentified (Additional file 1*). Nearly 8% of the species (21 of 279) were only resolved by ITS2, while 1% of the species (3 of 279) were resolved by both *mat*K and ITS2, while another 1% of the species were only resolved by *mat*K (Additional file 4). Seven congeneric species pairs lacked resolution with all three markers: *Rhododendron tomentosum & R. groenlandicum, Arctous alpina & A. ruber, Cerastium alpinum & C. beeringianum, Limnorchis huronensis & L. aquilonis, Leymus innovatus & L. mollis, Elymus trachycaulis & E. violaceus, Puccinellia nuttaliana & P. lucida.* In addition, two species (*Arctophila fulva* &*Dupontia fisheri*) in different, but closely allied, genera could not be discriminated. 

The 22 species of *Salix* presented a particularly dramatic case of compromised resolution. Despite their morphological distinctiveness, all but one (*Salix serissima*) of these species shared an identical *rbc*L haplotype. Thirteen *mat*K haplotypes were detected, but all were shared by more than one species. ITS2 also formed 11 polyphyletic clades which showed no congruence with the *mat*K clades. These results correspond with previous studies of phylogenetic relationships in willows based on *rbc*L, *mat*K and ITS [65,66], and likely reflect the impact of introgressive hybridisation [67,68]. Since members of the genus *Salix* comprised 8% of all species analyzed from Churchill, they substantially lowered the overall resolution of barcodes for the local flora.

## Conclusions

Rates of species-level resolution have varied widely in past tests of the efficacy of plant DNA barcodes. Some of this variation reflects the differing nature of the studies; some have adopted a floristic approach [15-19], while others have targeted a taxonomic assemblage [69,70]. Other variation in resolution success reflects differences in the number and combination of gene markers used. Our study tested the capacity of the standard plant markers, *rbc*L and *mat*K, and one of the most frequent supplemental markers, ITS2, to resolve the species-poor flora at Churchill. We observed 69% species-level resolution with the three-locus data including 41% resolution for congeneric taxa. The two marker combinations were less effective: *rbc*L &*mat*K delivered 54% (28% congeners) resolution, while *rbc*L & ITS2 produced 63% (37% congeners) resolution. As a result, the inclusion of *mat*K raised overall species resolution by 6% and the resolution of congeners by 4% from results based on just *rbc*L and ITS2. Comparison of *rbc*L & ITS2 versus *rbc*L &*mat*K demonstrated an overall rise of 9% in species resolution, all reflecting better resolution of congeners, suggesting that ITS2 will aid resolution of congeners in the more diverse floras.

ITS2 data helped to resolve closely related species in some families (Brassicaceae, Rosaceae, Poaceae, Cyperaceae) with relatively high MPD. However, it did not aid resolution in the Orchidaceae although this family had the highest MPD (3%), perhaps because it reflected high intra-specific variation in two monotypic orchid genera (*Corallorhiza trifida, Amerorchis rotundifolia*). Several cases of ITS2 paralogy were noted in the Juncaginaceae, Juncaceae, and Cyperaceae (2%), and a few cases (1%) of fungal and algal contamination were detected in herbarium samples, but these complications did not seriously compromise the utility of ITS2 in discriminating congeneric species.

The *mat*K sequence information did increase the stability of the phylogeny as the three gene phylogeny had higher MP-ratchet support scores than either of the two gene analyses. It also provided additional, and in some cases unique, diagnostic traits for species resolution (e.g.*, Calamagrostis*). However, the sensitivity of this marker to DNA degradation, the lack of universal primers and the complexity of sequence editing and alignment meant that the recovery of sequence information for this gene region was expensive. At present, the most cost-effective, rapid screening for the Churchill flora lies in the analysis of *rbc*L and ITS2.

The two standard DNA barcodes together with ITS2 delivered lower species-level resolution than those obtained in most prior floristic studies in the tropics and temperate zone. In part, this may reflect the fact that our study examined a higher percentage of the local flora than many earlier investigations, and may therefore, have included a higher percentage of closely related taxa. Although the number of species examined in previous analyses was similar to that in our study (approx. 300 species), they derived from much more diverse floras, e.g. the tree plots in Amazon and Panama examined just about 3% of the resident species [15,16,71]. The higher success in species resolution for 4600 species of medicinal plants [17] may reflect the inclusion of phylogenetically diverse taxa. The low species resolution at Churchill might additionally reflect lowered rates of molecular evolution in low diversity plant communities [24,25] and in low temperate settings, but disentangling the impacts of these varied factors will require further study.

## Abbreviations

BOLD: Barcode of Life Data System; CAN: National Herbarium of Canada, Canadian Museum of Nature; CCDB: Canadian Centre for DNA Barcoding; CNSC: Churchill Northern Studies Centre; KSR: Koffler Scientific Reserve, Ontario; MMMN: Herbarium at the Manitoba Museum, Winnipeg; MP: Maximum Parsimony analysis; MPD: Mean pairwise distance; OAC: Herbarium at the Biodiversity Institute of Ontario, University of Guelph; PAF: Checklist of the Panarctic Flora Vascular Plants; WIN: University of Manitoba Herbarium.

## Authors' contributions

MK designed the experiment, collected and identified plant material, generated and analysed molecular data, performed statistical analysis, and also drafted the manuscript. KJ planned and performed field investigations, and also collected and identified plant material. HB collected and identified plant material, aided in molecular analysis, and also helped draft the manuscript. PH aided study design, revised the manuscript, and also gained support for the work. All authors read and approved the final manuscript.

## Supplementary Material

Additional file 1 Specimen information with GenBank accession numbers. Click here for file

Additional file 2 Pseudogenes amplified with matK_390f/matK_1326r. Click here for file

Additional file 3 Cases of intraspecific variation. Click here for file

Additional file 4 Species resolution with different markers. Click here for file
