# Supplementary material for: Identification of the vascular plants of Churchill, Manitoba, using a DNA barcode library
Source: BMC Ecol. 2012 Nov 28;12:25. doi: 10.1186/1472-6785-12-25 (PMC3538695; doi:10.1186/1472-6785-12-25)
Supplement: Additional file 1 — Specimen information with GenBank accession numbers. [file 1472-6785-12-25-S1.docx]

| **Sample ID** | **Identification** | **GB *rbc*L** | **GB *mat*K** | **GB ITS2** | **Lat** | **Lon** | **Collection Date** | **Collectors** | **Institution Storing** |
| --- | --- | --- | --- | --- | --- | --- | --- | --- | --- |
| 09PROBE-05547 | *Equisetum fluviatile ** | JN965528 |  | JN999196 | 58.6920 | -94.1320 | 27-Jul-09 | M.Kuzmina | BIO |
| 09PROBE-05312 | *Equisetum scirpoides* | JN965526 |  |  | 58.6920 | -94.1320 | 21-Jul-2009 | K.Johnson | BIO |
| 09PROBE-05460 | *Equisetum variegatum* | JN965527 |  |  | 58.7720 | -94.1630 | 24-Jul-2009 | M.Kuzmina | BIO |
| 09PROBE-05068 | *Botrychium lunaria* | JN965301 |  |  | 58.3350 | -93.0180 | 19-Jul-2009 | B.Bennett | BIO |
| 09PROBE-05082 | *Botrychium lunaria* | JN965302 |  | JN998984 | 58.6290 | -93.7980 | 19-Jul-2009 | K.Johnson, M.Kuzmina | BIO |
| 09PROBE-05272 | *Botrychium lunaria* | JN965303 |  |  | 58.7340 | -94.1120 | 21-Jul-2009 | M.Kuzmina, K.Johnson | BIO |
| 09PROBE-05342 | *Botrychium lunaria* | JN965304 |  |  | 58.7890 | -94.2270 | 21-Jul-2009 | M.Kuzmina | BIO |
| 09PROBE-05059 | *Juniperus communis* | JN965611 |  | JN999275 | 58.6330 | -93.7900 | 19-Jul-2009 | K.Johnson, M.Kuzmina | BIO |
| 09PROBE-05243 | *Juniperus communis* | JN965612 |  |  | 58.1780 | -93.6370 | 21-Jul-2009 | B.Bennett | BIO |
| 09PROBE-05050 | *Larix laricina* | JN965623 |  |  | 58.6330 | -93.7900 | 19-Jul-2009 | K.Johnson, M.Kuzmina | BIO |
| 09PROBE-05308 | *Larix laricina* | JN965624 |  | JN999287 | 58.6920 | -94.1320 | 21-Jul-2009 | K.Johnson | BIO |
| 09PROBE-05052 | *Picea glauca* | JN965724 |  | JN999372 | 58.6330 | -93.7900 | 19-Jul-2009 | K.Johnson, M.Kuzmina | BIO |
| 09PROBE-05315 | *Picea mariana* | JN965725 |  |  | 58.6920 | -94.1320 | 21-Jul-2009 | K.Johnson | BIO |
| 09PROBE-05903 | *Lemna minor* | JN965625 |  |  | 57.1670 | -90.8330 | 06-Jul-1982 | K.L.Johnson | MMMN |
| 09PROBE-05300 | *Triglochin maritima* | JN966045 |  | JN999679 | 58.6260 | -94.2300 | 21-Jul-2009 | M.Kuzmina, K.Johnson | BIO |
| 09PROBE-05366 | *Triglochin maritima */*** | JN966048 | JN966720 | JN999683 | 58.7890 | -94.2270 | 21-Jul-2009 | M.Kuzmina | BIO |
| 09PROBE-05476 | *Triglochin maritima* | JN966046 | JN966718 | JN999681 | 58.7050 | -94.0520 | 26-Jul-2009 | M.Kuzmina | BIO |
| 09PROBE-05523 | *Triglochin maritima *** | JN966047 | JN966719 | JN999680 | 58.6260 | -94.2300 | 27-Jul-2009 | M.Kuzmina | BIO |
| 09PROBE-05398 | *Triglochin palustris *** | JN966049 | JN966721 | JN999682 | 58.7720 | -94.1630 | 24-Jul-2009 | M.Kuzmina | BIO |
| 09PROBE-05471 | *Potamogeton alpinus* | JN965760 | JN966452 | JN999404 | 58.7050 | -94.0520 | 26-Jul-2009 | M.Kuzmina | BIO |
| 09PROBE-05646 | *Potamogeton alpinus ** | JN966024 |  | JN999657 | 58.6260 | -94.2290 | 28-Jul-1984 | M.Zbigniewicz | WIN |
| 09PROBE-05649 | *Potamogeton alpinus *** | JN965759 | JN966451 |  | 58.6260 | -94.2290 | 07-Aug-2007 | D.Punter | WIN |
| 09PROBE-05651 | *Potamogeton perfoliatus ssp richardsonii *** | JN965761 | JN966453 | JN999405 | 58.6260 | -94.2290 | 12-Jul-1984 | H.J.Scoggan | WIN |
| 09PROBE-05644 | *Stuckenia filiformis *** | JN966022 | JN966697 | JN999659 | 58.6260 | -94.2290 | 04-Aug-1983 | M.Zbigniewicz | WIN |
| 09PROBE-05645 | *Stuckenia filiformis *** | JN966023 | JN966698 | JN999658 | 58.6260 | -94.2290 | 31-Jul-1984 | M.Zbigniewicz | WIN |
| 09PROBE-05647 | *Stuckenia filiformis *** | JN966025 | JN966699 | JN999660 | 58.6260 | -94.2290 | 01-Aug-1994 | G.M.Keleher | WIN |
| 09PROBE-05648 | *Stuckenia vaginata* | JN966026 |  |  | 58.6260 | -94.2290 |  |  | WIN |
| 09PROBE-05081 | *Tofieldia pusilla* | JN966039 | JN966712 | JN999675 | 58.6290 | -93.7980 | 19-Jul-2009 | K.Johnson, M.Kuzmina | BIO |
| 09PROBE-05204 | *Tofieldia pusilla* | JN966040 | JN966713 | JN999674 | 58.7470 | -93.8630 | 20-Jul-2009 | M.Kuzmina, K.Johnson | BIO |
| 09PROBE-05367 | *Tofieldia pusilla* | JN966041 | JN966714 | JN999673 | 58.7890 | -94.2270 | 21-Jul-2009 | M.Kuzmina | BIO |
| 09PROBE-05313 | *Maianthemum trifolium* | JN965667 | JN966366 |  | 58.6920 | -94.1320 | 21-Jul-2009 | K.Johnson | BIO |
| 09PROBE-05207 | *Amerorchis rotundifolia* | JN965223 | JN966073 | JN998913 | 58.7470 | -93.8630 | 20-Jul-2009 | M.Kuzmina, K.Johnson | BIO |
| 09PROBE-05378 | *Amerorchis rotundifolia *** | JN965224 | JN966074 | JN998910 | 58.6630 | -94.1670 | 22-Jul-2009 | H.Barron | BIO |
| 09PROBE-05542 | *Amerorchis rotundifolia* | JN965225 | JN966075 | JN998912 | 58.6920 | -94.1320 | 27-Jul-2009 | M.Kuzmina | BIO |
| 09PROBE-05600 | *Amerorchis rotundifolia* | JN965226 | JN966076 | JN998911 | 58.7470 | -94.1150 | 29-Jul-2009 | M.Kuzmina | BIO |
| 09PROBE-05810 | *Amerorchis rotundifolia* | JN965692 |  |  | 57.8550 | -92.7480 | 20-Jul-1970 | D.Punter | WIN |
| 09PROBE-05822 | *Amerorchis rotundifolia* | JN965227 |  | JN998908 | 57.8290 | -92.8020 | 19-Jul-2003 | E.Punter, M.Piercey-Normore | WIN |
| 09PROBE-05823 | *Amerorchis rotundifolia* | JN965228 |  | JN998909 | 57.8510 | -92.7580 | 01-Jul-1984 | M.Zbigniewicz | WIN |
| 09PROBE-05349 | *Corallorhiza trifida* | JN965452 | JN966248 | JN999128 | 58.7890 | -94.2270 | 21-Jul-2009 | M.Kuzmina | BIO |
| 09PROBE-05377 | *Corallorhiza trifida* | JN965453 | JN966249 | JN999127 | 58.6630 | -94.1670 | 22-Jul-2009 | H.Barron | BIO |
| 09PROBE-05382 | *Corallorhiza trifida* | JN965454 | JN966250 | JN999126 | 57.3510 | -93.5230 | 21-Jul-2009 | S.Ponomarenko | BIO |
| 09PROBE-05397 | *Corallorhiza trifida* | JN965455 | JN966251 | JN999124 | 58.7600 | -94.0880 | 22-Jul-2009 | M.Kuzmina, K.Johnson | BIO |
| 09PROBE-05548 | *Corallorhiza trifida* | JN965456 | JN966252 | JN999125 | 58.6920 | -94.1320 | 27-Jul-2009 | M.Kuzmina | BIO |
| 09PROBE-05278 | *Cypripedium passerinum* | JN965458 |  |  | 58.7340 | -94.1120 | 21-Jul-2009 | M.Kuzmina, K.Johnson | BIO |
| 09PROBE-05535 | *Cypripedium passerinum *** | JN965459 | JN966253 | JN999130 | 58.6260 | -94.2300 | 27-Jul-2009 | M.Kuzmina | BIO |
| 09PROBE-05819 | *Cypripedium passerinum* | JN965460 |  | JN999131 | 57.8560 | -92.7530 | 04-Jul-1984 | M.Zbigniewicz | WIN |
| 09PROBE-05820 | *Cypripedium passerinum* | JN965461 |  |  | 57.8350 | -92.7960 | 20-Jul-1970 | D.Punter | WIN |
| 09PROBE-05821 | *Cypripedium passerinum* | JN965462 |  | JN999129 | 57.8510 | -92.7580 | 15-Jul-1983 | M.Zbigniewicz | WIN |
| 09PROBE-05310 | *Limnorchis aquilonis ** | JN965639 | JN966352 | JN999297 | 58.6920 | -94.1320 | 21-Jul-2009 | K.Johnson | BIO |
| 09PROBE-05376 | *Limnorchis aquilonis ** | JN965640 | JN966353 |  | 58.6630 | -94.1670 | 22-Jul-2009 | H.Barron | BIO |
| 09PROBE-05811 | *Limnorchis aquilonis *** | JN965632 | JN966351 | JN999295 | 57.8510 | -92.7580 | 05-Jul-1971 | R.E.Longton | WIN |
| 09PROBE-05812 | *Limnorchis aquilonis* | JN965633 |  | JN999294 | 57.8300 | -92.8080 | 22-Jul-2003 | E.Punter, M.Piercey-Normore | WIN |
| 09PROBE-05815 | *Limnorchis huronensis* | JN965636 |  |  | 57.8300 | -92.8080 | 28-Jul-1994 | G.M.Keleher | WIN |
| 09PROBE-05816 | *Limnorchis huronensis* | JN965637 |  | JN999296 | 57.8300 | -92.8080 | 19-Aug-1972 | J.Hunt | WIN |
| 09PROBE-05817 | *Limnorchis huronensis* | JN965638 |  |  | 57.8300 | -92.8080 | 29-Jul-1983 | M.Zbigniewicz | WIN |
| 09PROBE-05464 | *Limnorchis hyperborea* | JN965641 | JN966354 | JN999298 | 58.7310 | -93.7800 | 24-Jul-2009 | M.Kuzmina | BIO |
| 09PROBE-05532 | *Limnorchis hyperborea *** | JN965642 | JN966355 |  | 58.6260 | -94.2300 | 27-Jul-2009 | M.Kuzmina | BIO |
| 09PROBE-05813 | *Limnorchis hyperborea ** | JN965634 |  | JN999293 | 57.8370 | -92.8170 | 20-Jul-2003 | E.Punter, M.Piercey-Normore | WIN |
| 09PROBE-05814 | *Limnorchis hyperborea ** | JN965635 |  | JN999292 | 57.8370 | -92.8170 | 19-Jul-2003 | E.Punter | WIN |
| 09PROBE-05461 | *Listera cordata* | JN965648 | JN966362 | JN999304 | 58.7310 | -93.7800 | 24-Jul-2009 | P.D.N. Hebert | BIO |
| 09PROBE-05208 | *Lysiella obtusata* | JN965664 | JN966363 | N999314 | 58.7470 | -93.8630 | 20-Jul-2009 | M.Kuzmina, K.Johnson | BIO |
| 09PROBE-05276 | *Lysiella obtusata* | JN965665 | JN966364 | JN999316 | 58.7340 | -94.1120 | 21-Jul-2009 | M.Kuzmina, K.Johnson | BIO |
| 09PROBE-05347 | *Lysiella obtusata* | JN965666 | JN966365 | JN999315 | 58.7890 | -94.2270 | 21-Jul-2009 | M.Kuzmina | BIO |
| 09PROBE-05034 | *Carex aquatilis* | JN965331 | JN966167 | JN999013 | 58.7390 | -94.1120 | 18-Jul-2009 | K.Johnson, M.Kuzmina | BIO |
| 09PROBE-05360 | *Carex aquatilis* | JN965332 | JN966168 |  | 58.7890 | -94.2270 | 21-Jul-2009 | M.Kuzmina | BIO |
| 09PROBE-05498 | *Carex aquatilis* | JN965333 | JN966169 | JN999012 | 58.7800 | -94.1950 | 27-Jul-2009 | M.Kuzmina | BIO |
| 09PROBE-05522 | *Carex aquatilis* | JN965334 | JN966170 | JN999014 | 58.6260 | -94.2300 | 27-Jul-2009 | M.Kuzmina | BIO |
| 09PROBE-05240 | *Carex atrofusca* | JN965339 | JN966172 | JN999015 | 58.7470 | -93.8630 | 20-Jul-2009 | M.Kuzmina, K.Johnson | BIO |
| 09PROBE-05736 | *Carex atrofusca* | JN965335 |  | JN999018 | 58.7400 | -93.8200 | 19-Jul-2003 | E.Punter, M.Piercey-Normore | WIN |
| 09PROBE-05737 | *Carex atrofusca* | JN965336 |  | JN999019 | 58.7400 | -93.8200 | 23-Jul-2003 | E.Punter, M.Piercey-Normore | WIN |
| 09PROBE-05738 | *Carex atrofusca *** | JN965337 | JN966171 | JN999016 | 58.7400 | -93.8200 | 22-Jul-2003 | E.Punter, M.Piercey-Normore | WIN |
| 09PROBE-05739 | *Carex atrofusca* | JN965338 |  | JN999017 | 57.8350 | -92.7960 | 23-Jul-2003 | E.Punter, M.Piercey-Normore | WIN |
| 09PROBE-05740 | *Carex aurea* | JN965340 |  |  | 57.8380 | -92.7940 | 20-Aug-1983 | M.Zbigniewicz | WIN |
| 09PROBE-05741 | *Carex aurea* | JN965341 |  | JN999020 | 58.4670 | -93.2100 | 26-Jul-1969 | D.Punter | WIN |
| 09PROBE-05742 | *Carex aurea* | JN965342 |  |  | 58.4080 | -93.0660 | 15-Jul-1973 | K.L.Johnson | WIN |
| 09PROBE-05743 | *Carex bicolor* | JN965343 |  |  | 57.8530 | -92.7580 | 29-Jul-1994 | G.M.Keleher | WIN |
| 09PROBE-05744 | *Carex canescens* | JN965344 |  | JN999021 | 58.3040 | -92.9820 | 07-Aug-2007 | D.Punter | WIN |
| 09PROBE-05399 | *Carex capillaris ** | JN965378 | JN966190 | JN999051 | 58.7720 | -94.1630 | 24-Jul-2009 | M.Kuzmina | BIO |
| 09PROBE-05576 | *Carex capillaris* | JN965350 |  | JN999022 | 58.7690 | -93.8560 | 29-Jul-2009 | M.Kuzmina | BIO |
| 09PROBE-05582 | *Carex capillaris* | JN965351 | JN966174 |  | 58.7450 | -93.8950 | 29-Jul-2009 | M.Kuzmina | BIO |
| 09PROBE-05745 | *Carex capillaris* | JN965345 |  | JN999027 | 58.7600 | -93.2200 | 13-Jul-1983 | J.M.Shay | WIN |
| 09PROBE-05746 | *Carex capillaris* | JN965346 |  | JN999026 | 58.7670 | -93.2510 | 22-Jul-2003 | E.Punter, M.Piercey-Normore | WIN |
| 09PROBE-05747 | *Carex capillaris *** | JN965347 | JN966173 | JN999025 | 58.4070 | -93.0620 | 21-Jul-2003 | E.Punter | WIN |
| 09PROBE-05748 | *Carex capillaris* | JN965348 |  | JN999024 | 57.0030 | -92.3040 | 30-Jul-1994 | G.M.Keleher | WIN |
| 09PROBE-05749 | *Carex capillaris* | JN965349 |  | JN999023 | 57.0030 | -92.3040 | 19-Jul-2003 | E.Punter, M.Piercey-Normore | WIN |
| 09PROBE-05750 | *Carex capitata* | JN965352 |  | JN999028 | 57.0030 | -92.3040 | 23-Jul-2003 | E.Punter, M.Piercey-Normore | WIN |
| 09PROBE-05751 | *Carex capitata *** | JN965353 | JN966175 | JN999029 | 57.0030 | -92.3040 | 20-Jul-2003 | E.Punter, M.Piercey-Normore | WIN |
| 09PROBE-05254 | *Carex chordorrhiza* | JN965354 |  | JN999030 | 58.1780 | -93.6370 | 21-Jul-2009 | B.Bennett | BIO |
| 09PROBE-05550 | *Carex chordorrhiza *** | JN965355 | JN966176 | JN999032 | 58.7870 | -93.7200 | 25-Jul-2009 | M.Kuzmina | BIO |
| 09PROBE-05032 | *Carex concinna *** | JN965356 | JN966177 |  | 58.7390 | -94.1120 | 18-Jul-2009 | K.Johnson, M.Kuzmina | BIO |
| 09PROBE-05054 | *Carex concinna* | JN965357 | JN966178 | JN999035 | 58.6330 | -93.7900 | 19-Jul-2009 | K.Johnson, M.Kuzmina | BIO |
| 09PROBE-05089 | *Carex concinna* | JN965358 | JN966179 | N999034 | 58.6290 | -93.7980 | 19-Jul-2009 | K.Johnson, M.Kuzmina | BIO |
| 09PROBE-05390 | *Carex concinna* | JN965359 | JN966180 | JN999033 | 58.7600 | -94.0880 | 22-Jul-2009 | M.Kuzmina, K.Johnson | BIO |
| 09PROBE-05752 | *Carex garberi* | JN965360 |  | JN999036 | 57.0030 | -92.3040 | 20-Jul-2003 | E.Punter, M.Piercey-Normore | WIN |
| 09PROBE-05753 | *Carex garberi* | JN965361 |  |  | 57.0030 | -92.3040 | 26-Jul-1969 | D.Punter | WIN |
| 09PROBE-05754 | *Carex garberi* | JN965362 |  | JN999037 | 57.0030 | -92.3040 | 21-Jul-2002 | B.A.Ford at al. | WIN |
| 09PROBE-05072 | *Carex glacialis* | JN965367 |  |  | 58.3350 | -93.0180 | 19-Jul-2009 | B.Bennett | BIO |
| 09PROBE-05628 | *Carex glacialis *** | JN965368 | JN966183 |  | 58.7570 | -94.0740 | 30-Jul-2009 | M.Kuzmina | BIO |
| 09PROBE-05757 | *Carex glacialis* | JN965363 |  | JN999040 | 57.0030 | -92.3040 | 19-Jul-2002 | B.A.Ford at al. | WIN |
| 09PROBE-05758 | *Carex glacialis *** | JN965364 | JN966181 | JN999041 | 57.0030 | -92.3040 | 19-Jul-2003 | E.Punter, M.Piercey-Normore | WIN |
| 09PROBE-05759 | *Carex glacialis* | JN965365 |  | JN999038 | 57.0030 | -92.3040 | 16-Jul-2004 | B.A.Ford at al. | WIN |
| 09PROBE-05761 | *Carex glacialis *** | JN965366 | JN966182 | JN999039 | 57.0030 | -92.3040 | 20-Jul-2002 | B.A.Ford at al. | WIN |
| 09PROBE-05029 | *Carex glareosa *** | JN965371 | JN966184 | JN999043 | 58.7390 | -94.1120 | 18-Jul-2009 | K.Johnson, M.Kuzmina | BIO |
| 09PROBE-05340 | *Carex glareosa* | JN965372 | JN966185 | JN999044 | 58.7890 | -94.2270 | 21-Jul-2009 | M.Kuzmina | BIO |
| 09PROBE-05573 | *Carex glareosa *** | JN965373 | JN966186 |  | 58.7690 | -93.8560 | 29-Jul-2009 | M.Kuzmina | BIO |
| 09PROBE-05755 | *Carex glareosa* | JN965369 |  | JN999046 | 57.0030 | -92.3040 | 05-Jul-1984 | M.Zbigniewicz | WIN |
| 09PROBE-05756 | *Carex glareosa* | JN965370 |  | JN999045 | 57.0030 | -92.3040 | 12-Jul-1983 | M.Zbigniewicz | WIN |
| 09PROBE-05239 | *Carex gynocrates* | JN965374 | JN966187 | JN999050 | 58.7470 | -93.8630 | 20-Jul-2009 | M.Kuzmina, K.Johnson | BIO |
| 09PROBE-05375 | *Carex gynocrates* | JN965375 | JN966188 | JN999047 | 58.7890 | -94.2270 | 21-Jul-2009 | M.Kuzmina | BIO |
| 09PROBE-05487 | *Carex gynocrates* | JN965376 |  | JN999048 | 58.7870 | -93.7200 | 25-Jul-2009 | M.Kuzmina | BIO |
| 09PROBE-05544 | *Carex gynocrates* | JN965377 | JN966189 | N999049 | 58.6920 | -94.1320 | 27-Jul-2009 | M.Kuzmina | BIO |
| 09PROBE-05769 | *Carex livida* | JN965380 |  | N999053 | 57.0030 | -92.3040 | 20-Aug-1983 | M.Zbigniewicz | WIN |
| 09PROBE-05770 | *Carex mackenziei* | JN965381 |  | N999056 | 57.0030 | -92.3040 | 02-Jul-1984 | M.Zbigniewicz | WIN |
| 09PROBE-05771 | *Carex mackenziei* | JN965382 |  | JN999055 | 58.4670 | -93.2100 | 06-Aug-1983 | M.Zbigniewicz | WIN |
| 09PROBE-05772 | *Carex mackenziei* | JN965383 |  | JN999054 | 58.6780 | -93.1450 | 14-Jul-1983 | M.Zbigniewicz | WIN |
| 09PROBE-05022 | *Carex maritima* | JN965384 | JN966192 | JN999058 | 58.7710 | -93.8510 | 18-Jul-2009 | K.Johnson, M.Kuzmina | BIO |
| 09PROBE-05222 | *Carex maritima* | JN965385 | JN966193 | JN999059 | 58.7640 | -93.8970 | 20-Jul-2009 | M.Kuzmina, K.Johnson | BIO |
| 09PROBE-05318 | *Carex maritima *** | JN965386 | JN966194 | JN999060 | 58.7610 | -94.0370 | 20-Jul-2009 | M.Kuzmina, K.Johnson | BIO |
| 09PROBE-05365 | *Carex maritima* | JN965387 | JN966195 | JN999057 | 58.7890 | -94.2270 | 21-Jul-2009 | M.Kuzmina | BIO |
| 09PROBE-05400 | *Carex media* | JN965388 | JN966196 | JN999061 | 58.7720 | -94.1630 | 24-Jul-2009 | M.Kuzmina | BIO |
| 09PROBE-05774 | *Carex microglochin* | JN965389 |  | JN999062 | 58.4670 | -93.2100 | 13-Jul-1983 | J.M.Shay | WIN |
| 09PROBE-05775 | *Carex microglochin* | JN965390 |  | JN999063 | 58.4670 | -93.2100 | 19-Jul-2002 | B.A.Ford | WIN |
| 09PROBE-05776 | *Carex microglochin* | JN965391 |  | N999064 | 57.8350 | -92.7960 | 23-Jul-2003 | E.Punter, M.Piercey-Normore | WIN |
| 09PROBE-05489 | *Carex paupercula ** | JN965395 | JN966200 | JN999066 | 58.7870 | -93.7200 | 25-Jul-2009 | M.Kuzmina | BIO |
| 09PROBE-05236 | *Carex rariflora* | JN965392 | JN966197 | JN999067 | 58.7710 | -93.8430 | 20-Jul-2009 | M.Kuzmina, K.Johnson | BIO |
| 09PROBE-05259 | *Carex rariflora* | JN965393 | JN966198 | JN999068 | 58.1780 | -93.6370 | 21-Jul-2009 | B.Bennett | BIO |
| 09PROBE-05372 | *Carex rariflora* | JN965394 | JN966199 | JN999069 | 58.7890 | -94.2270 | 21-Jul-2009 | M.Kuzmina | BIO |
| 09PROBE-05248 | *Carex rotundata ** | JN965379 | JN966191 | JN999052 | 58.1780 | -93.6370 | 21-Jul-2009 | B.Bennett | BIO |
| 09PROBE-05260 | *Carex rotundata* | JN965396 | JN966201 | JN999070 | 58.1780 | -93.6370 | 21-Jul-2009 | B.Bennett | BIO |
| 09PROBE-05572 | *Carex rotundata *** |  | JN966202 |  | 58.7690 | -93.8560 | 29-Jul-2009 | M.Kuzmina | BIO |
| 09PROBE-05626 | *Carex rotundata* | JN965397 | JN966203 | JN999073 | 58.7630 | -94.0870 | 30-Jul-2009 | M.Kuzmina | BIO |
| 09PROBE-05779 | *Carex rotundata* | JN965398 |  | JN999072 | 57.8300 | -92.8080 | 19-Jul-2002 | B.A.Ford at al. | WIN |
| 09PROBE-05780 | *Carex rotundata* | JN965399 |  | JN999071 | 57.8300 | -92.8080 | 20-Jul-2002 | B.A.Ford at al. | WIN |
| 09PROBE-05781 | *Carex rupestris* | JN965400 |  | JN999074 | 57.8300 | -92.8080 | 20-Jul-2002 | B.A.Ford at al. | WIN |
| 09PROBE-05782 | *Carex rupestris *** | JN965401 | JN966204 | JN999077 | 57.8300 | -92.8080 | 19-Jul-2002 | B.A.Ford at al. | WIN |
| 09PROBE-05784 | *Carex rupestris *** |  | JN966205 | JN999076 | 57.8550 | -92.7480 | 14-Jul-1993 | G.M.Keleher | WIN |
| 09PROBE-05789 | *Carex saxatilis* | JN965402 |  | JN999078 | 57.8370 | -92.8170 | 21-Jul-2002 | B.A.Ford at al. | WIN |
| 09PROBE-05790 | *Carex saxatilis* | JN965403 |  | JN999080 | 58.3830 | -93.2250 | 14-Jul-1993 | G.M.Keleher | WIN |
| 09PROBE-05791 | *Carex saxatilis* | JN965404 |  | JN999081 | 58.3830 | -93.2250 | 20-Aug-1983 | M.Zbigniewicz | WIN |
| 09PROBE-05792 | *Carex saxatilis* | JN965405 |  | JN999082 | 58.3830 | -93.2250 | 20-Jul-1994 | G.M.Keleher, E.Punter | WIN |
| 09PROBE-05793 | *Carex saxatilis* | JN965406 |  | JN999083 | 58.3830 | -93.2250 | 23-Jul-2003 | E.Punter, M.Piercey-Normore | WIN |
| 09PROBE-05794 | *Carex saxatilis* | JN965407 | JN966206 | JN999084 | 58.3830 | -93.2250 | 23-Jul-2003 | E.Punter, M.Piercey-Normore | WIN |
| 09PROBE-05091 | *Carex scirpoidea* | JN965408 | JN966207 | JN999086 | 58.6290 | -93.7980 | 19-Jul-2009 | K.Johnson, M.Kuzmina | BIO |
| 09PROBE-05100 | *Carex scirpoidea *** | JN965409 | JN966208 | JN999085 | 58.6330 | -93.7900 | 19-Jul-2009 | K.Johnson, M.Kuzmina | BIO |
| 09PROBE-05785 | *Carex subspathacea* | JN965411 |  | JN999089 | 57.8300 | -92.8080 | 05-Jul-1983 | M.Zbigniewicz | WIN |
| 09PROBE-05786 | *Carex subspathacea* | JN965412 |  | JN999090 | 57.8300 | -92.8080 | 16-Jul-1983 | M.Zbigniewicz | WIN |
| 09PROBE-05787 | *Carex subspathacea* | JN965413 |  | JN999087 | 57.8300 | -92.8080 | 02-Aug-1983 | M.Zbigniewicz | WIN |
| 09PROBE-05795 | *Carex utriculata *** |  | JN966210 | JN999091 | 58.3830 | -93.2250 | 07-Aug-2007 | D.Punter | WIN |
| 09PROBE-05092 | *Carex vaginata* | JN965414 |  | JN999095 | 58.6290 | -93.7980 | 19-Jul-2009 | K.Johnson, M.Kuzmina | BIO |
| 09PROBE-05206 | *Carex vaginata *** | JN965415 | JN966211 |  | 58.7470 | -93.8630 | 20-Jul-2009 | M.Kuzmina, K.Johnson | BIO |
| 09PROBE-05246 | *Carex vaginata* | JN965416 |  |  | 58.1780 | -93.6370 | 21-Jul-2009 | B.Bennett | BIO |
| 09PROBE-05264 | *Carex vaginata* | JN965417 | JN966212 | JN999096 | 58.7340 | -94.1120 | 21-Jul-2009 | M.Kuzmina, K.Johnson | BIO |
| 09PROBE-05541 | *Carex vaginata* | JN965418 | JN966213 | JN999092 | 58.6920 | -94.1320 | 27-Jul-2009 | M.Kuzmina | BIO |
| 09PROBE-05543 | *Carex vaginata* | JN965419 | JN966214 | N999094 | 58.6920 | -94.1320 | 27-Jul-2009 | M.Kuzmina | BIO |
| 09PROBE-05575 | *Carex vaginata* | JN965420 |  | JN999093 | 58.7690 | -93.8560 | 29-Jul-2009 | M.Kuzmina | BIO |
| 09PROBE-05250 | *Carex williamsii* | JN965421 | JN966215 | JN999097 | 58.1780 | -93.6370 | 21-Jul-2009 | B.Bennett | BIO |
| 09PROBE-05357 | *Carex williamsii* | JN965422 | JN966216 | JN999098 | 58.7890 | -94.2270 | 21-Jul-2009 | M.Kuzmina | BIO |
| 09PROBE-05574 | *Carex williamsii* | JN965423 | JN966217 |  | 58.7690 | -93.8560 | 29-Jul-2009 | M.Kuzmina | BIO |
| 09PROBE-05778 | *Carex sp.* | JN965457 |  |  | 57.8380 | -92.7940 | 12-Jul-2004 | B.A.Ford at al. | WIN |
| 09PROBE-05796 | *Eleocharis acicularis* | JN965500 |  |  | 58.6730 | -93.2560 | 23-Jul-2002 | B.A.Ford at al. | WIN |
| 09PROBE-05797 | *Eleocharis acicularis* | JN965501 |  | JN999170 | 58.6730 | -93.2560 | 03-Aug-1983 | M.Zbigniewicz | WIN |
| 09PROBE-05456 | *Eleocharis palustris *** | JN965502 | JN966276 | JN999172 | 58.7720 | -94.1630 | 24-Jul-2009 | M.Kuzmina | BIO |
| 09PROBE-05800 | *Eleocharis palustris* | JN965504 |  | JN999171 | 58.4670 | -93.2100 | 03-Aug-1983 | M.Zbigniewicz | WIN |
| 09PROBE-05801 | *Eleocharis uniglumis* | JN965505 |  | JN999175 | 58.3830 | -93.2570 | 01-Jul-1969 | D.Punter | WIN |
| 09PROBE-05802 | *Eleocharis uniglumis* | JN965506 |  | JN999174 | 57.7880 | -93.3790 | 26-Jul-1970 | D.Punter | WIN |
| 09PROBE-05298 | *Eriophorum angustifolium* | JN965529 | JN966288 | JN999197 | 58.6260 | -94.2300 | 21-Jul-2009 | M.Kuzmina, K.Johnson | BIO |
| 09PROBE-05345 | *Eriophorum angustifolium* | JN965530 | JN966289 | JN999198 | 58.7890 | -94.2270 | 21-Jul-2009 | M.Kuzmina | BIO |
| 09PROBE-05450 | *Eriophorum angustifolium* | JN965531 | JN966290 | JN999200 | 58.7720 | -94.1630 | 24-Jul-2009 | M.Kuzmina | BIO |
| 09PROBE-05475 | *Eriophorum angustifolium* | JN965532 | JN966291 | JN999199 | 58.7050 | -94.0520 | 26-Jul-2009 | M.Kuzmina | BIO |
| 09PROBE-05521 | *Eriophorum angustifolium* | JN965533 | JN966292 | JN999201 | 58.6260 | -94.2300 | 27-Jul-2009 | M.Kuzmina | BIO |
| 09PROBE-05477 | *Eriophorum callitrix* | JN965535 | JN966294 | JN999202 | 58.7050 | -94.0520 | 26-Jul-2009 | M.Kuzmina | BIO |
| 09PROBE-05803 | *Eriophorum callitrix* | JN965536 |  | JN999206 | 59.8300 | -92.8080 | 22-Jul-2003 | E.Punter, M.Piercey-Normore | WIN |
| 09PROBE-05804 | *Eriophorum callitrix* | JN965537 |  | JN999204 | 57.8370 | -92.8170 | 11-Jul-2004 | B.A.Ford at al. | WIN |
| 09PROBE-05805 | *Eriophorum callitrix* | JN965538 |  | JN999205 | 57.7920 | -93.3660 | 02-Jul-1983 | J.M.Shay | WIN |
| 09PROBE-05346 | *Eriophorum scheuchzeri ** | JN965534 | JN966293 | JN999203 | 58.7890 | -94.2270 | 21-Jul-2009 | M.Kuzmina | BIO |
| 09PROBE-05808 | *Eriophorum scheuchzeri* | JN965540 |  | JN999209 | 57.8510 | -92.7580 | 15-Jul-1973 | K.L.Johnson | WIN |
| 09PROBE-05577 | *Kobresia myosuroides *** | JN965618 | JN966345 | JN999280 | 58.7690 | -93.8560 | 29-Jul-2009 | M.Kuzmina | BIO |
| 09PROBE-05768 | *Kobresia myosuroides* | JN965617 |  | JN999281 | 57.0030 | -92.3040 | 30-Jul-1994 | G.M.Keleher | WIN |
| 09PROBE-05763 | *Kobresia simpliciuscula* | JN965619 |  |  | 57.0030 | -92.3040 | 19-Jul-2003 | E.Punter, M.Piercey-Normore | WIN |
| 09PROBE-05764 | *Kobresia simpliciuscula* | JN965620 |  | JN999286 | 57.0030 | -92.3040 | 21-Jul-2003 | E.Punter, M.Piercey-Normore | WIN |
| 09PROBE-05765 | *Kobresia simpliciuscula* | JN965621 |  | JN999285 | 57.0030 | -92.3040 | 05-Aug-1983 | M.Zbigniewicz | WIN |
| 09PROBE-05766 | *Kobresia simpliciuscula* | JN965622 |  | JN999282 | 57.0030 | -92.3040 | 30-Jul-1994 | G.M.Keleher | WIN |
| 09PROBE-05540 | *Trichophorum sp.* | JN966042 | JN966715 | JN999676 | 58.6920 | -94.1320 | 27-Jul-2009 | M.Kuzmina | BIO |
| 09PROBE-05078 | *Trichophorum caespitosum *** | JN966043 | JN966716 | JN999677 | 58.6290 | -93.7980 | 19-Jul-2009 | K.Johnson, M.Kuzmina | BIO |
| 09PROBE-05374 | *Trichophorum caespitosum* | JN966044 | JN966717 | N999678 | 58.7890 | -94.2270 | 21-Jul-2009 | M.Kuzmina | BIO |
| 09PROBE-05454 | *Juncus alpinoarticulatus* | JN965593 |  | JN999259 | 58.7720 | -94.1630 | 24-Jul-2009 | M.Kuzmina | BIO |
| 09PROBE-05712 | *Juncus alpinoarticulatus* | JN965591 |  | JN999261 | 58.7510 | -93.8230 | 14-Jul-1984 | M.Zbigniewicz | WIN |
| 09PROBE-05713 | *Juncus alpinoarticulatus* | JN965592 |  | JN999260 | 58.7400 | -93.8200 | 29-Jul-1994 | G.M.Keleher | WIN |
| 09PROBE-05714 | *Juncus alpinoarticulatus ** | JN965590 |  | JN999258 | 58.7400 | -93.8200 | 14-Jul-1983 | M.Zbigniewicz | WIN |
| 09PROBE-05720 | *Juncus arcticus ssp alaskanus* | JN965594 |  | JN999262 | 58.7400 | -93.8200 | 18-Jul-2002 | B.A.Ford at al. | WIN |
| 09PROBE-05221 | *Juncus arcticus ssp arcticus* | JN965595 |  | JN999264 | 58.7640 | -93.8970 | 20-Jul-2009 | M.Kuzmina, K.Johnson | BIO |
| 09PROBE-05270 | *Juncus arcticus ssp arcticus *** | JN965596 | JN966338 |  | 58.7340 | -94.1120 | 21-Jul-2009 | M.Kuzmina, K.Johnson | BIO |
| 09PROBE-05281 | *Juncus arcticus ssp arcticus *** | JN965597 | JN966339 | JN999263 | 58.6260 | -94.2300 | 21-Jul-2009 | M.Kuzmina, K.Johnson | BIO |
| 09PROBE-05719 | *Juncus arcticus var. alaskanus* | JN965598 |  | JN999265 | 58.7400 | -93.8200 | 26-Jul-1994 | G.M.Keleher | WIN |
| 09PROBE-05717 | *Juncus balticus* | JN965599 |  | JN999266 | 58.7400 | -93.8200 | 20-Jul-2002 | B.A.Ford, et al. | WIN |
| 09PROBE-05718 | *Juncus balticus* | JN965600 |  | JN999267 | 58.7400 | -93.8200 | 19-Jul-2003 | E.Punter, M.Piercey-Normore | WIN |
| 09PROBE-05721 | *Juncus bufonius* | JN965601 |  | JN999269 | 58.7400 | -93.8200 | 30-Jul-1983 | M.Zbigniewicz | WIN |
| 09PROBE-05722 | *Juncus bufonius* | JN965602 |  | JN999268 | 58.7400 | -93.8200 | 29-Jul-1969 | D.Punter | WIN |
| 09PROBE-05715 | *Juncus castaneus* | JN965603 |  | JN999271 | 58.7400 | -93.8200 | 21-Jul-2003 | E.Punter | WIN |
| 09PROBE-05716 | *Juncus castaneus* | JN965604 |  | JN999270 | 58.7400 | -93.8200 | 22-Jul-2003 | E.Punter, M.Piercey-Normore | WIN |
| 09PROBE-05723 | *Juncus triglumis* | JN965605 |  | JN999272 | 58.7400 | -93.8200 | 23-Jul-2003 | E.Punter, M.Piercey-Normore | WIN |
| 09PROBE-05725 | *Juncus triglumis* | JN965607 |  | JN999273 | 58.7400 | -93.8200 | 05-Aug-1983 | M.Zbigniewicz | WIN |
| 09PROBE-05726 | *Juncus triglumis* | JN965608 |  | JN999274 | 58.7400 | -93.8200 | 20-Jul-2002 | B.A.Ford, et al. | WIN |
| 09PROBE-05727 | *Juncus triglumis* | JN965609 |  |  | 58.7400 | -93.8200 | 19-Jul-2002 | B.A.Ford, et al. | WIN |
| 09PROBE-05731 | *Luzula sp.* | JN965649 |  | JN999305 | 58.7400 | -93.8200 | 20-Jul-2002 | B.A.Ford, et al. | WIN |
| 09PROBE-05732 | *Luzula sp.* | JN965650 |  |  | 58.7400 | -93.8200 | 01-Jul-1973 | K.L.Johnson | WIN |
| 09PROBE-05729 | *Luzula confusa* | JN965651 |  |  | 58.7400 | -93.8200 | 21-Jul-2003 | E.Punter, M.Piercey-Normore | WIN |
| 09PROBE-05730 | *Luzula confusa* | JN965652 |  |  | 58.7400 | -93.8200 | 18-Jul-2002 | B.A.Ford, et al. | WIN |
| 09PROBE-05733 | *Luzula groenlandica* | JN965655 |  | JN999306 | 58.7400 | -93.8200 | 05-Aug-1983 | M.Zbigniewicz | WIN |
| 09PROBE-05900 | *Luzula groenlandica* | JN965656 |  |  | 57.0020 | -92.3060 | 23-Jul-1997 | D.R.M.Hatch | WIN |
| 09PROBE-05901 | *Luzula groenlandica* | JN965654 |  |  | 57.1670 | -90.8330 | 06-Jul-1982 | K.L.Johnson | MMMN |
| 09PROBE-05902 | *Luzula groenlandica* | JN965653 |  | JN999307 | 59.0670 | -94.8000 | 15-Jul-1973 | K.L.Johnson | MMMN |
| 09PROBE-05465 | *Luzula parviflora* | JN965659 |  | JN999312 | 58.7310 | -93.7800 | 24-Jul-2009 | M.Kuzmina | BIO |
| 09PROBE-05734 | *Luzula parviflora* | JN965657 |  | JN999309 | 58.7400 | -93.8200 | 20-Jul-2003 | E.Punter | WIN |
| 09PROBE-05735 | *Luzula parviflora* | JN965658 |  | JN999310 | 58.7400 | -93.8200 | 05-Jul-1971 | R.E.Longton | WIN |
| 09PROBE-05897 | *Luzula parviflora* | JN965660 |  | N999308 | 57.8290 | -92.8020 | 01-Aug-1985 | R.Staniforth | WIN |
| 09PROBE-05898 | *Luzula parviflora* | JN965661 |  | JN999311 | 58.3340 | -93.2250 | 12-Jul-1986 | K.L.Johnson | WIN |
| 09PROBE-05899 | *Luzula parviflora* | JN965662 |  |  | 57.0020 | -92.3060 | 15-Jul-1996 | K.L.Johnson | WIN |
| 09PROBE-05654 | *Agrostis scabra *** | JN965216 | JN966066 | JN998901 | 58.7720 | -93.8450 | 28-Jul-1949 | H.J.Scoggan | WIN |
| 09PROBE-05655 | *Alopecurus aequalis* | JN965218 | JN966068 | JN998903 | 58.7860 | -94.2020 | 28-Jul-1949 | H.J.Scoggan | WIN |
| 09PROBE-05656 | *Alopecurus aequalis *** | JN965219 | JN966069 | JN998904 | 58.7860 | -94.2020 | 30-Jul-1983 | M.Zbigniewicz | WIN |
| 09PROBE-05657 | *Alopecurus borealis* | JN965220 | JN966070 | JN998906 | 58.7400 | -93.8200 | 10-Jul-1983 | Prov.of Man-Wildlife branch | WIN |
| 09PROBE-05658 | *Alopecurus borealis* | JN965221 | JN966071 | JN998905 | 58.7400 | -93.8200 | 20-Jul-2002 | B.A.Ford | WIN |
| 09PROBE-05659 | *Alopecurus borealis* | JN965222 | JN966072 | JN998907 | 58.7400 | -93.8200 | 20-Jul-2002 | B.A.Ford | WIN |
| 09PROBE-05449 | *Arctagrostis latifolia *** | JN965257 | JN966103 | JN998935 | 58.7720 | -94.1630 | 24-Jul-2009 | M.Kuzmina | BIO |
| 09PROBE-05665 | *Arctophila fulva* | JN965260 | JN966106 | JN998941 | 58.6300 | -93.8190 | 26-Jul-1994 | G.M.Keleher | WIN |
| 09PROBE-05666 | *Arctophila fulva* | JN965261 |  | JN998940 | 58.6260 | -94.2290 | 10-Jul-1983 | J.M.Shay | WIN |
| 09PROBE-05667 | *Arctophila fulva* | JN965262 | JN966107 | JN998939 | 58.6260 | -94.2290 | 18-Jul-2002 | B.A.Ford | WIN |
| 09PROBE-05668 | *Arctophila fulva* | JN965263 | JN966108 | JN998938 | 58.6260 | -94.2290 | 21-Jul-2003 | E.Punter | WIN |
| 09PROBE-05660 | *Beckmannia syzigachne* | JN965291 | JN966134 | JN998974 | 58.7330 | -93.7330 | 08-Aug-1953 | E.Beckett | WIN |
| 09PROBE-05661 | *Beckmannia syzigachne* | JN965292 |  | JN998971 | 58.6300 | -93.8190 |  | H.J.Scoggan | WIN |
| 09PROBE-05662 | *Beckmannia syzigachne* | JN965293 | JN966135 | JN998972 | 58.6300 | -93.8190 | 11-Aug-1983 | M.Zbigniewicz | WIN |
| 09PROBE-05663 | *Beckmannia syzigachne* | JN965294 | JN966136 | JN998973 | 58.6300 | -93.8190 | 20-Jul-1970 | D.Punter | WIN |
| 09PROBE-05664 | *Bromopsis inermis* | JN965305 | JN966145 | JN998985 | 58.6300 | -93.8190 | 15-Jul-1953 | E.Beckett | WIN |
| 09PROBE-05515 | *Calamagrostis canadensis * /*** | JN965308 | JN966148 | JN998987 | 58.7700 | -94.1720 | 27-Jul-2009 | M.Kuzmina | BIO |
| 09PROBE-05674 | *Calamagrostis canadensis *** | JN965309 | JN966149 | JN998992 | 58.7500 | -94.1100 | 07-Aug-2007 | D.Punter | WIN |
| 09PROBE-05675 | *Calamagrostis canadensis *** | JN965310 | JN966150 | JN998991 | 58.7500 | -94.1100 | 01-Aug-1975 | W.Krivda | WIN |
| 09PROBE-05676 | *Calamagrostis canadensis *** | JN965311 | JN966151 | JN998990 | 58.7500 | -94.1100 | 28-Jul-1949 | H.J.Scoggan | WIN |
| 09PROBE-05669 | *Calamagrostis deschampsioides *** | JN965313 | JN966153 | JN998995 | 58.7500 | -94.1100 | 21-Jul-1984 | M.Zbigniewicz | WIN |
| 09PROBE-05670 | *Calamagrostis deschampsioides* | JN965314 | JN966154 | JN998994 | 58.7500 | -94.1100 | 02-Aug-1983 | M.Zbigniewicz | WIN |
| 09PROBE-05671 | *Calamagrostis deschampsioides *** | JN965315 | JN966155 | JN998993 | 58.7500 | -94.1100 | 16-Jul-1983 | M.Zbigniewicz | WIN |
| 09PROBE-05672 | *Calamagrostis deschampsioides *** | JN965316 | JN966156 | JN998997 | 58.7500 | -94.1100 | 25-Jul-1983 | M.Zbigniewicz | WIN |
| 09PROBE-05673 | *Calamagrostis deschampsioides* | JN965317 |  | JN998996 | 58.7500 | -94.1100 | 14-Jul-1983 | J.M.Shay | WIN |
| 09PROBE-05679 | *Calamagrostis deschampsioides */*** | JN965320 | JN966158 | JN998998 | 58.7500 | -94.1100 | 23-Jul-1984 | M.Zbigniewicz | WIN |
| 09PROBE-05682 | *Calamagrostis deschampsioides */*** | JN965306 | JN966146 | JN998986 | 58.7500 | -94.1100 | 14-Jul-2004 | B.A.Ford | WIN |
| 09PROBE-05688 | *Calamagrostis deschampsioides */*** | JN965307 | JN966147 | JN998988 | 58.7340 | -94.1120 | 21-Jul-2003 | E.Punter | WIN |
| 09PROBE-05396 | *Calamagrostis neglecta ** | JN965312 | JN966152 | N998989 | 58.7650 | -94.1420 | 22-Jul-2009 | M.Kuzmina | BIO |
| 09PROBE-05677 | *Calamagrostis neglecta* | JN965318 |  | JN999002 | 58.7500 | -94.1100 | 21-Jul-1984 | M.Zbigniewicz | WIN |
| 09PROBE-05678 | *Calamagrostis neglecta *** | JN965319 | JN966157 | JN999001 | 58.7500 | -94.1100 | 04-Aug-1983 | M.Zbigniewicz | WIN |
| 09PROBE-05680 | *Calamagrostis neglecta *** | JN965321 | JN966159 | JN999000 | 58.7500 | -94.1100 | 02-Aug-1983 | M.Zbigniewicz | WIN |
| 09PROBE-05681 | *Calamagrostis neglecta *** | JN965322 | JN966160 | JN998999 | 58.7500 | -94.1100 | 21-Jul-2003 | E Punter, M.Piercey-Normore | WIN |
| 09PROBE-05303 | *Deschampsia cespitosa* | JN965469 | JN966256 | JN999138 | 58.6260 | -94.2300 | 21-Jul-2009 | M.Kuzmina, K.Johnson | BIO |
| 09PROBE-05686 | *Deschampsia cespitosa *** | JN965466 | JN966254 | JN999139 | 58.7500 | -94.1100 | 13-Jul-1983 | J.M.Shay | WIN |
| 09PROBE-05687 | *Deschampsia cespitosa *** | JN965467 | JN966255 | JN999137 | 58.7340 | -94.1120 | 19-Jul-2003 | E.Punter | WIN |
| 09PROBE-05689 | *Deschampsia cespitosa* | JN965468 |  | JN999136 | 58.6920 | -94.1320 | 14-Jul-1984 | M.Zbigniewicz | WIN |
| 09PROBE-05683 | *Dupontia fisheri *** | JN965497 | JN966274 | JN999168 | 58.7500 | -94.1100 | 12-Jul-1983 | J.M.Shay | WIN |
| 09PROBE-05684 | *Dupontia fisheri *** | JN965498 | JN966275 | JN999167 | 58.7500 | -94.1100 | 04-Aug-1983 | M.Zbigniewicz | WIN |
| 09PROBE-05685 | *Dupontia fisheri* | JN965499 |  | JN999169 | 58.7500 | -94.1100 | 22-Jul-1970 | D.Punter | WIN |
| 09PROBE-05551 | *Elymus trachycaulus* | JN965509 | JN966279 |  | 58.6200 | -93.8310 | 28-Jul-2009 | H.Barron | BIO |
| 09PROBE-05613 | *Elymus trachycaulus *** | JN965510 | JN966280 | JN999176 | 58.7180 | -94.1240 | 29-Jul-2009 | M.Kuzmina | BIO |
| 09PROBE-05638 | *Elymus trachycaulus* | JN965507 | JN966277 | JN999177 | 58.7050 | -94.0520 | 15-Jun-1982 | K.Frego | WIN |
| 09PROBE-05639 | *Elymus trachycaulus *** | JN965508 | JN966278 | JN999178 | 58.6620 | -94.1650 | 22-Jun-1988 | D.Punter | WIN |
| 09PROBE-05640 | *Elymus violaceus* | JN965511 |  | JN999180 | 58.6620 | -94.1650 | 05-Aug-1954 | J.C.R. | WIN |
| 09PROBE-05653 | *Elymus violaceus* | JN965512 |  | JN999179 | 58.7720 | -93.8450 | 23-Jul-1970 | D.Punter | WIN |
| 09PROBE-05327 | *Festuca brachyphylla* | JN965548 | JN966301 | JN999215 | 58.7890 | -94.2270 | 21-Jul-2009 | M.Kuzmina | BIO |
| 09PROBE-05603 | *Festuca brachyphylla* | JN965549 | JN966302 | JN999218 | 58.7180 | -94.1240 | 29-Jul-2009 | M.Kuzmina | BIO |
| 09PROBE-05690 | *Festuca brachyphylla *** | JN965545 | JN966298 | JN999216 | 50.0470 | -95.0690 | 09-Aug-1972 | J.Hunt | WIN |
| 09PROBE-05691 | *Festuca brachyphylla *** | JN965546 | JN966299 | JN999217 | 56.3530 | -94.7140 | 01-Aug-1990 | G.M.Keleher | WIN |
| 09PROBE-05692 | *Festuca brachyphylla *** | JN965547 | JN966300 | JN999214 | 57.0500 | -90.0690 | 23-Jul-2002 | B.A.Ford | WIN |
| 09PROBE-05693 | *Festuca rubra *** | JN965550 | JN966303 | JN999219 | 58.6600 | -93.1900 | 19-Jul-2003 | E.Punter | WIN |
| 09PROBE-05694 | *Festuca rubra ssp richardsonii *** | JN965551 | JN966304 | JN999220 | 57.8300 | -92.8080 | 24-Jul-1949 | H.J.Scoggan | WIN |
| 09PROBE-05695 | *Hierochloe alpina *** | JN965577 | JN966327 | JN999245 | 58.7680 | -93.8680 | 15-Jul-1973 | K.L.Johnson | WIN |
| 09PROBE-05038 | *Hierochloe odorata* | JN965578 | JN966328 | JN999247 | 58.7390 | -94.1120 | 18-Jul-2009 | K.Johnson, M.Kuzmina | BIO |
| 09PROBE-05083 | *Hierochloe odorata* | JN965579 | JN966329 | JN999246 | 58.6290 | -93.7980 | 19-Jul-2009 | K.Johnson, M.Kuzmina | BIO |
| 09PROBE-05268 | *Hierochloe odorata* | JN965580 | JN966330 | JN999248 | 58.7340 | -94.1120 | 21-Jul-2009 | M.Kuzmina, K.Johnson | BIO |
| 09PROBE-05696 | *Hierochloe pauciflora *** | JN965581 | JN966331 | JN999249 | 59.0670 | -94.8000 | 24-Jul-2002 | B.A.Ford, et al. | WIN |
| 09PROBE-05697 | *Leymus innovatus* | JN965627 |  |  | 59.0670 | -94.8000 | 08-Aug-1953 | E.Beckett | WIN |
| 09PROBE-05698 | *Leymus innovatus *** | JN965628 | JN966347 |  | 58.7360 | -93.7890 | 19-Jul-2003 | E.Punter | WIN |
| 09PROBE-05226 | *Leymus mollis* | JN965629 | JN966348 | JN999290 | 58.7710 | -93.8430 | 20-Jul-2009 | M.Kuzmina, K.Johnson | BIO |
| 09PROBE-05284 | *Leymus mollis* | JN965630 | JN966349 | JN999289 | 58.6260 | -94.2300 | 21-Jul-2009 | M.Kuzmina, K.Johnson | BIO |
| 09PROBE-05479 | *Leymus mollis* | JN965631 | JN966350 | JN999291 | 58.7920 | -93.7510 | 25-Jul-2009 | M.Kuzmina | BIO |
| 09PROBE-05062 | *Poa alpina* | JN965736 | JN966429 | JN999384 | 58.6330 | -93.7900 | 19-Jul-2009 | K.Johnson, M.Kuzmina | BIO |
| 09PROBE-05111 | *Poa alpina* | JN965737 | JN966430 |  | 58.6170 | -93.8120 | 19-Jul-2009 | M.Kuzmina, K.Johnson | BIO |
| 09PROBE-05304 | *Poa alpina* | JN965738 | JN966431 | JN999383 | 58.6260 | -94.2300 | 21-Jul-2009 | M.Kuzmina, K.Johnson | BIO |
| 09PROBE-05451 | *Poa alpina* | JN965739 | JN966432 | JN999385 | 58.7720 | -94.1630 | 24-Jul-2009 | M.Kuzmina | BIO |
| 09PROBE-05531 | *Poa alpina* | JN965743 | JN966436 | JN999386 | 58.6260 | -94.2300 | 27-Jul-2009 | M.Kuzmina | BIO |
| 09PROBE-05373 | *Poa arctica* | JN965742 | JN966435 | JN999388 | 58.7890 | -94.2270 | 21-Jul-2009 | M.Kuzmina | BIO |
| 09PROBE-05704 | *Poa arctica *** | JN965740 | JN966433 | JN999387 | 58.7330 | -93.8080 |  |  | WIN |
| 09PROBE-05705 | *Poa arctica *** | JN965741 | JN966434 | JN999389 | 58.7320 | -93.8220 |  |  | WIN |
| 09PROBE-05393 | *Poa glauca* | JN965744 | JN966437 | JN999394 | 58.7650 | -94.1420 | 22-Jul-2009 | M.Kuzmina | BIO |
| 09PROBE-05455 | *Poa glauca* | JN965752 | JN966445 | JN999397 | 58.7720 | -94.1630 | 24-Jul-2009 | M.Kuzmina | BIO |
| 09PROBE-05494 | *Poa glauca* | JN965746 | JN966439 | JN999393 | 58.7800 | -94.1950 | 27-Jul-2009 | M.Kuzmina | BIO |
| 09PROBE-05525 | *Poa glauca* | JN965747 | JN966440 | JN999391 | 58.6260 | -94.2300 | 27-Jul-2009 | M.Kuzmina | BIO |
| 09PROBE-05602 | *Poa glauca* | JN965735 | JN966428 | JN999381 | 58.7180 | -94.1240 | 29-Jul-2009 | M.Kuzmina | BIO |
| 09PROBE-05604 | *Poa glauca* | JN965748 | JN966441 | JN999392 | 58.7180 | -94.1240 | 29-Jul-2009 | M.Kuzmina | BIO |
| 09PROBE-05707 | *Poa palustris *** | JN965750 | JN966443 | JN999395 | 58.7320 | -93.8220 |  |  | WIN |
| 09PROBE-05112 | *Poa pratensis ssp alpigena* | JN965751 | JN966444 |  | 58.6170 | -93.8120 | 19-Jul-2009 | M.Kuzmina, K.Johnson | BIO |
| 09PROBE-05394 | *Poa pratensis ssp alpigena* | JN965745 | JN966438 | JN999390 | 58.7650 | -94.1420 | 22-Jul-2009 | M.Kuzmina | BIO |
| 09PROBE-05446 | *Poa pratensis ssp alpigena *** | JN965734 | JN966427 | JN999382 | 58.7720 | -94.1630 | 24-Jul-2009 | M.Kuzmina | BIO |
| 09PROBE-05701 | *Poa pratensis ssp alpigena* | JN965753 |  | JN999399 | 58.7340 | -93.8110 | 20-Jul-2003 | E.Punter | WIN |
| 09PROBE-05702 | *Poa pratensis ssp alpigena *** | JN965754 | JN966446 | JN999398 | 58.7340 | -93.8110 | 11-Jul-1984 | M.Zbigniewicz | WIN |
| 09PROBE-05703 | *Poa pratensis ssp alpigena *** | JN965755 | JN966447 | JN999400 | 58.7330 | -93.7830 | 28-Jul-1994 | G.M.Keleher | WIN |
| 09PROBE-05706 | *Poa pratensis ssp alpigena *** | JN965749 | JN966442 | JN999396 | 58.7320 | -93.8220 |  |  | WIN |
| 09PROBE-05699 | *Puccinellia langeana *** | JN965784 | JN966477 | JN999429 | 58.7330 | -93.7830 | 30-Jul-1984 | M.Zbigniewicz | WIN |
| 09PROBE-05700 | *Puccinellia lucida *** | JN965785 | JN966478 | JN999430 | 58.7510 | -93.8230 | 29-Jul-1994 | G.M.Keleher | WIN |
| 09PROBE-05395 | *Puccinellia nuttalliana* | JN965787 | JN966480 | JN999431 | 58.7650 | -94.1420 | 22-Jul-2009 | M.Kuzmina | BIO |
| 09PROBE-05708 | *Puccinellia nuttalliana *** | JN965786 | JN966479 | JN999432 | 58.7330 | -93.8080 | 25-Jul-1949 | H.J.Scoggan | WIN |
| 09PROBE-05709 | *Puccinellia phryganodes *** | JN965788 | JN966481 | JN999433 | 58.7500 | -93.8470 | 02-Aug-1983 | M.Zbigniewicz | WIN |
| 09PROBE-05711 | *Puccinellia phryganodes *** | JN965789 | JN966482 | JN999434 | 58.7510 | -93.8230 | 26-Jul-1994 | G.M.Keleher | WIN |
| 09PROBE-05110 | *Trisetum spicatum* | JN966052 | JN966724 | JN999686 | 58.6170 | -93.8120 | 19-Jul-2009 | M.Kuzmina, K.Johnson | BIO |
| 09PROBE-05283 | *Trisetum spicatum* | JN966053 | JN966725 | JN999687 | 58.6260 | -94.2300 | 21-Jul-2009 | M.Kuzmina, K.Johnson | BIO |
| 09PROBE-05338 | *Trisetum spicatum* | JN966054 | JN966726 | JN999688 | 58.7890 | -94.2270 | 21-Jul-2009 | M.Kuzmina | BIO |
| 09PROBE-05641 | *Sparganium natans* | JN966010 | JN966685 |  | 58.6260 | -94.2290 | 07-Aug-2007 | D.Punter | WIN |
| 09PROBE-05642 | *Sparganium natans* | JN966011 | JN966686 |  | 58.6260 | -94.2290 | 29-Jul-1994 | D.Punter | WIN |
| 09PROBE-05643 | *Sparganium natans* | JN966012 | JN966687 | JN999646 | 58.6260 | -94.2290 | 01-Aug-2009 | G.M.Keleher | WIN |
| 09PROBE-05215 | *Anemone multifida* |  | JN966084 |  | 58.6200 | -93.8310 | 20-Jul-2009 | H.Barron | BIO |
| 09PROBE-05317 | *Anemone multifida* | JN965238 | JN966085 | JN998923 | 58.7610 | -94.0370 | 20-Jul-2009 | M.Kuzmina, K.Johnson | BIO |
| 09PROBE-05356 | *Anemone multifida* | JN965239 | JN966086 | JN998921 | 58.7890 | -94.2270 | 21-Jul-2009 | M.Kuzmina | BIO |
| 09PROBE-05500 | *Anemone multifida* | JN965240 | JN966087 | JN998924 | 58.7800 | -94.1950 | 27-Jul-2009 | M.Kuzmina | BIO |
| 09PROBE-05611 | *Anemone multifida* | JN965241 | JN966088 | JN998925 | 58.7180 | -94.1240 | 29-Jul-2009 | M.Kuzmina | BIO |
| 09PROBE-05881 | *Anemone multifida* | JN965237 |  |  | 57.0020 | -92.3060 | 24-Jul-1979 | K.L.Johnson | MMMN |
| 09PROBE-05882 | *Anemone multifida* | JN965236 |  | JN998922 | 57.1670 | -90.8330 | 09-Jul-1982 | K.L.Johnson | MMMN |
| 09PROBE-05033 | *Anemone parviflora* | JN965244 | JN966091 |  | 58.7390 | -94.1120 | 18-Jul-2009 | K.Johnson, M.Kuzmina | BIO |
| 09PROBE-05096 | *Anemone parviflora* | JN965245 | JN966092 |  | 58.6290 | -93.7980 | 19-Jul-2009 | K.Johnson, M.Kuzmina | BIO |
| 09PROBE-05877 | *Anemone parviflora* | JN965235 | JN966083 | JN998920 | 57.8460 | -92.7500 | 24-Jul-1999 | K.Monson | MMMN |
| 09PROBE-05878 | *Anemone parviflora* | JN965243 | JN966090 |  | 58.6300 | -93.8190 | 12-Jul-1986 | K.L.Johnson | MMMN |
| 09PROBE-05879 | *Anemone parviflora *** | JN965242 | JN966089 |  | 57.8460 | -92.7500 | 24-Jul-1999 | K.Monson | MMMN |
| 09PROBE-05217 | *Anemone richardsonii* | JN965246 | JN966093 | JN998926 | 58.7640 | -93.8970 | 20-Jul-2009 | P.D.N. Hebert | BIO |
| 09PROBE-05293 | *Anemone richardsonii* | JN965247 | JN966094 | JN998927 | 58.6260 | -94.2300 | 21-Jul-2009 | M.Kuzmina, K.Johnson | BIO |
| 09PROBE-05579 | *Caltha palustris *** | JN965325 | JN966162 | JN999005 | 58.7450 | -93.8950 | 29-Jul-2009 | M.Kuzmina | BIO |
| 09PROBE-05875 | *Caltha palustris* | JN965324 |  | JN999004 | 59.0670 | -94.8000 | 15-Jul-1973 | K.L.Johnson | MMMN |
| 09PROBE-05492 | *Cyrtorhyncha cymbalaria* | JN965464 |  | JN999132 | 58.7800 | -94.1950 | 27-Jul-2009 | M.Kuzmina | BIO |
| 09PROBE-05558 | *Cyrtorhyncha cymbalaria* | JN965465 |  | JN999135 | 58.7690 | -93.8560 | 29-Jul-2009 | M.Kuzmina | BIO |
| 09PROBE-05866 | *Cyrtorhyncha cymbalaria* | JN965463 |  | JN999134 | 58.6300 | -93.8190 | 24-Aug-1973 | K.L.Johnson | MMMN |
| 09PROBE-05873 | *Ranunculus acris* | JN965796 |  | JN999441 | 57.0030 | -92.3040 | 21-Jul-1979 | K.L.Johnson | MMMN |
| 09PROBE-05874 | *Ranunculus acris* | JN965795 |  | JN999440 | 57.0030 | -92.3040 | 11-Jul-1969 | D.Punter | MMMN |
| 09PROBE-05481 | *Ranunculus aquatilis* | JN965799 | JN966488 | JN999442 | 58.7870 | -93.7200 | 25-Jul-2009 | M.Kuzmina | BIO |
| 09PROBE-05869 | *Ranunculus aquatilis* | JN965798 |  | JN999445 | 57.1670 | -90.8330 | 06-Sep-1988 | J.E.Christie | MMMN |
| 09PROBE-05872 | *Ranunculus aquatilis* | JN965797 |  | JN999444 | 56.3830 | -94.5000 | 05-Sep-1986 | S.Kroker | MMMN |
| 09PROBE-05030 | *Ranunculus arcticus* | JN965800 | JN966489 | JN999447 | 58.7220 | -93.4280 | 18-Jul-2009 | B.Bennett | BIO |
| 09PROBE-05064 | *Ranunculus arcticus *** | JN965801 | JN966490 | JN999446 | 58.3350 | -93.0180 | 19-Jul-2009 | B.Bennett | BIO |
| 09PROBE-05231 | *Ranunculus arcticus *** | JN965802 | JN966491 | JN999449 | 58.7710 | -93.8430 | 20-Jul-2009 | M.Kuzmina, K.Johnson | BIO |
| 09PROBE-05330 | *Ranunculus arcticus* | JN965803 |  | JN999448 | 58.7890 | -94.2270 | 21-Jul-2009 | M.Kuzmina | BIO |
| 09PROBE-05296 | *Thalictrum venulosum* | JN966037 |  | JN999671 | 58.6260 | -94.2300 | 21-Jul-2009 | M.Kuzmina, K.Johnson | BIO |
| 09PROBE-05853 | *Atriplex sp.** | JN965444 | JN966239 | JN999115 | 58.7400 | -93.8200 | 01-Aug-1985 | R.Staniforth | MMMN |
| 09PROBE-05854 | *Atriplex sp.*** |  | JN966238 | JN999114 | 58.7630 | -93.8850 | 01-Aug-1985 | R.Staniforth | MMMN |
| 09PROBE-05856 | *Atriplex glabriuscula* | JN965284 | JN966128 | JN998964 | 58.7400 | -93.8200 | 25-Aug-1973 | K.L.Johnson | MMMN |
| 09PROBE-05857 | *Atriplex glabriuscula *** | JN965283 | JN966127 | JN998963 | 59.0670 | -94.8000 | 15-Jul-1973 | K.L.Johnson | MMMN |
| 09PROBE-05859 | *Atriplex glabriuscula* | JN965282 | JN966126 | JN998962 | 59.0830 | -94.8000 | 13-Jul-1973 | K.L.Johnson | MMMN |
| 09PROBE-05860 | *Atriplex glabriuscula *** |  | JN966125 | JN998961 | 58.7630 | -93.8850 | 17-Jul-1984 | K.L.Johnson | MMMN |
| 09PROBE-05571 | *Chenopodium sp.** | JN965285 | JN966129 | JN998966 | 58.7690 | -93.8560 | 29-Jul-2009 | M.Kuzmina | BIO |
| 09PROBE-05848 | *Chenopodium capitatum* | JN965448 | JN966244 |  | 56.6170 | -93.8670 | 07-Jul-1990 | D.R.M.Hatch | MMMN |
| 09PROBE-05849 | *Chenopodium capitatum* | JN965447 | JN966243 | JN999119 | 56.5060 | -94.1070 | 11-Jul-1985 | S.Kroker | MMMN |
| 09PROBE-05850 | *Chenopodium capitatum* | JN965446 | JN966242 | JN999118 | 58.6300 | -93.8190 | 26-Aug-1973 | K.L.Johnson | MMMN |
| 09PROBE-05851 | *Chenopodium capitatum* |  | JN966241 | JN999117 | 58.6300 | -93.8190 | 24-Aug-1976 | K.L.Johnson | MMMN |
| 09PROBE-05852 | *Chenopodium capitatum* | JN965445 | JN966240 | JN999116 | 57.0030 | -92.3040 | 19-Jul-1970 | D.Punter | MMMN |
| 09PROBE-05862 | *Salicornia maritima* | JN965832 | JN966519 | JN999474 | 58.7500 | -93.4160 | 02-Sep-1982 | D.Bazely | MMMN |
| 09PROBE-05863 | *Salicornia maritima* | JN965831 |  | JN999473 | 58.7810 | -94.1870 | 09-Aug-1982 | K.L.Johnson | MMMN |
| 09PROBE-05861 | *Salicornia rubra* | JN965833 |  | JN999475 | 58.7340 | -94.1120 | 04-Aug-1980 | R.Staniforth | MMMN |
| 09PROBE-05865 | *Suaeda calceoliformis* | JN966027 | JN966700 | JN999661 | 58.7340 | -94.1120 | 29-Jul-1985 | R.Staniforth | MMMN |
| 09PROBE-05027 | *Arenaria humifusa* | JN965271 | JN966116 | JN998949 | 58.7390 | -94.1120 | 18-Jul-2009 | K.Johnson, M.Kuzmina | BIO |
| 09PROBE-05845 | *Cerastium alpinum* |  | JN966223 | JN999102 | 58.7400 | -93.8200 | 03-Jul-1981 | K.L.Johnson | MMMN |
| 09PROBE-05846 | *Cerastium alpinum* | JN965428 | JN966222 |  | 58.7400 | -93.8200 | 26-Jun-1981 | K.L.Johnson | MMMN |
| 09PROBE-05847 | *Cerastium alpinum* |  | JN966224 |  | 58.7400 | -93.8200 | 13-Jul-1990 | K.L.Johnson | MMMN |
| 09PROBE-05090 | *Cerastium beeringianum* | JN965429 | JN966225 | JN999105 | 58.6290 | -93.7980 | 19-Jul-2009 | K.Johnson, M.Kuzmina | BIO |
| 09PROBE-05361 | *Cerastium beeringianum* | JN965430 | JN966226 | JN999104 | 58.7890 | -94.2270 | 21-Jul-2009 | M.Kuzmina | BIO |
| 09PROBE-05383 | *Cerastium beeringianum* | JN965431 | JN966227 | JN999103 | 58.6630 | -94.1670 | 22-Jul-2009 | H.Barron | BIO |
| 09PROBE-05568 | *Cerastium beeringianum* | JN965432 |  |  | 58.7690 | -93.8560 | 29-Jul-2009 | M.Kuzmina | BIO |
| 09PROBE-05230 | *Honckenya peploides* | JN965588 | JN966337 | JN999256 | 58.7710 | -93.8430 | 20-Jul-2009 | M.Kuzmina, K.Johnson | BIO |
| 09PROBE-05553 | *Honckenya peploides* | JN965589 |  | JN999257 | 58.7690 | -93.8560 | 29-Jul-2009 | M.Kuzmina | BIO |
| 09PROBE-05839 | *Minuartia rubella* | JN965677 | JN966377 | JN999321 | 58.7400 | -93.8200 | 20-Jul-1994 | K.L.Johnson | MMMN |
| 09PROBE-05840 | *Minuartia rubella* | JN965676 | JN966376 | JN999324 | 58.7400 | -93.8200 | 10-Jul-1985 | K.L.Johnson | MMMN |
| 09PROBE-05841 | *Minuartia rubella* | JN965675 | JN966375 | JN999319 | 58.7380 | -93.8190 | 12-Jul-1990 | K.L.Johnson | MMMN |
| 09PROBE-05842 | *Minuartia rubella* | JN965674 | JN966374 | JN999322 | 58.7400 | -93.8200 | 12-Jul-1986 | K.L.Johnson | MMMN |
| 09PROBE-05843 | *Minuartia rubella* | JN965673 | JN966373 | JN999323 | 58.7500 | -94.1100 | 12-Aug-1982 | K.L.Johnson | MMMN |
| 09PROBE-05844 | *Minuartia rubella* |  | JN966372 | JN999320 | 58.7400 | -93.8200 | 27-Jun-1981 | K.L.Johnson | MMMN |
| 09PROBE-05838 | *Moehringia lateriflora* | JN965680 | JN966379 | JN999327 | 57.0030 | -92.3040 | 12-Jul-1969 | D.Punter | MMMN |
| 09PROBE-05587 | *Silene sp.* | JN965994 | JN966669 | JN999632 | 58.7470 | -94.1150 | 29-Jul-2009 | M.Kuzmina | BIO |
| 09PROBE-05832 | *Silene sp.* | JN965993 | JN966668 | JN999631 | 58.7810 | -94.1870 | 01-Aug-1985 | R.Staniforth | MMMN |
| 09PROBE-05228 | *Silene involucrata* | JN966000 | JN966676 | JN999638 | 58.7710 | -93.8430 | 20-Jul-2009 | M.Kuzmina, K.Johnson | BIO |
| 09PROBE-05286 | *Silene involucrata* | JN966001 | JN966677 |  | 58.6260 | -94.2300 | 21-Jul-2009 | M.Kuzmina, K.Johnson | BIO |
| 09PROBE-05341 | *Silene involucrata* | JN966002 | JN966678 | JN999637 | 58.7890 | -94.2270 | 21-Jul-2009 | M.Kuzmina | BIO |
| 09PROBE-05830 | *Silene involucrata* | JN965999 | JN966675 | JN999634 | 58.7400 | -93.8200 | 04-Jul-1942 | A.Simpson | MMMN |
| 09PROBE-05833 | *Silene involucrata* |  | JN966674 | JN999633 | 58.7400 | -93.8200 | 28-Jun-1970 | F.P.Ralston | MMMN |
| 09PROBE-05834 | *Silene involucrata* | JN965998 | JN966673 |  | 58.7500 | -94.1100 | 07-Jul-1985 | K.L.Johnson | MMMN |
| 09PROBE-05835 | *Silene involucrata* | JN965997 | JN966672 |  | 58.7380 | -93.8190 | 05-Jul-1981 | K.L.Johnson | MMMN |
| 09PROBE-05836 | *Silene involucrata* | JN965996 | JN966671 | JN999636 | 58.7380 | -93.8190 | 24-Jul-1996 | J.Klapecki | MMMN |
| 09PROBE-05837 | *Silene involucrata* | JN965995 | JN966670 | JN999635 | 57.8460 | -92.7500 | 15-Jul-1999 | K.Monson | MMMN |
| 09PROBE-05555 | *Stellaria longifolia* | JN966016 | JN966691 | JN999647 | 58.7690 | -93.8560 | 29-Jul-2009 | M.Kuzmina | BIO |
| 09PROBE-05826 | *Stellaria longifolia* | JN966015 | JN966690 | JN999648 | 57.0020 | -92.3060 | 21-Jul-1979 | K.L.Johnson | MMMN |
| 09PROBE-05827 | *Stellaria longifolia* | JN966014 | JN966689 | JN999651 | 57.0030 | -92.3040 | 30-Jul-1969 | D.Punter | MMMN |
| 09PROBE-05041 | *Stellaria longipes* | JN966017 | JN966692 | JN999652 | 58.7500 | -94.1100 | 18-Jul-2009 | K.Johnson, M.Kuzmina | BIO |
| 09PROBE-05282 | *Stellaria longipes* | JN966018 | JN966693 | JN999656 | 58.6260 | -94.2300 | 21-Jul-2009 | M.Kuzmina, K.Johnson | BIO |
| 09PROBE-05322 | *Stellaria longipes* | JN966019 | JN966694 | JN999654 | 58.7890 | -94.2270 | 21-Jul-2009 | M.Kuzmina | BIO |
| 09PROBE-05335 | *Stellaria longipes* | JN966020 | JN966695 | JN999653 | 58.7890 | -94.2270 | 21-Jul-2009 | M.Kuzmina | BIO |
| 09PROBE-05452 | *Stellaria longipes* | JN966021 | JN966696 | JN999655 | 58.7720 | -94.1630 | 24-Jul-2009 | M.Kuzmina | BIO |
| 09PROBE-05242 | *Drosera anglica *** | JN965492 | JN966271 |  | 58.1780 | -93.6370 | 21-Jul-2009 | B.Bennett | BIO |
| 09PROBE-05369 | *Drosera anglica* | JN965493 |  | JN999163 | 57.3510 | -93.5230 | 21-Jul-2009 | S.Ponomarenko | BIO |
| 09PROBE-05370 | *Drosera rotundifolia* | JN965494 |  | JN999164 | 57.3510 | -93.5230 | 21-Jul-2009 | S.Ponomarenko | BIO |
| 09PROBE-05444 | *Bistorta vivipara* | JN965298 | JN966142 | JN998981 | 58.7720 | -94.1630 | 24-Jul-2009 | M.Kuzmina | BIO |
| 09PROBE-05559 | *Bistorta vivipara* | JN965299 | JN966143 | JN998980 | 58.7690 | -93.8560 | 29-Jul-2009 | M.Kuzmina | BIO |
| 09PROBE-05620 | *Bistorta vivipara* | JN965300 | JN966144 | JN998982 | 58.6260 | -94.2300 | 29-Jul-2009 | M.Kuzmina | BIO |
| 09PROBE-05517 | *Rumex sp. *** | JN965829 | JN966516 |  | 58.7700 | -94.1720 | 27-Jul-2009 | M.Kuzmina | BIO |
| 09PROBE-05516 | *Rumex occidentalis *** | JN965830 | JN966517 |  | 58.7700 | -94.1720 | 27-Jul-2009 | M.Kuzmina | BIO |
| 09PROBE-05533 | *Parnassia kotzebuei* | JN965704 | JN966398 |  | 58.6260 | -94.2300 | 27-Jul-2009 | M.Kuzmina | BIO |
| 09PROBE-05534 | *Parnassia kotzebuei* | JN965705 | JN966399 |  | 57.3510 | -93.5230 | 21-Jul-2009 | S.Ponomarenko | BIO |
| 09PROBE-05591 | *Parnassia kotzebuei* | JN965706 | JN966400 | JN999353 | 58.7470 | -94.1150 | 29-Jul-2009 | M.Kuzmina | BIO |
| 09PROBE-05306 | *Parnassia palustris ssp neogaena* | JN965707 | JN966401 | JN999356 | 58.6260 | -94.2300 | 21-Jul-2009 | M.Kuzmina, K.Johnson | BIO |
| 09PROBE-05484 | *Parnassia palustris ssp neogaena* | JN965708 | JN966402 | JN999355 | 58.7920 | -93.7510 | 25-Jul-2009 | M.Kuzmina | BIO |
| 09PROBE-05502 | *Parnassia palustris ssp neogaena* | JN965709 | JN966403 | JN999354 | 58.7800 | -94.1950 | 27-Jul-2009 | M.Kuzmina | BIO |
| 09PROBE-05049 | *Geocaulon lividum* | JN965571 | JN966321 | JN999241 | 58.6330 | -93.7900 | 19-Jul-2009 | K.Johnson, M.Kuzmina | BIO |
| 09PROBE-05249 | *Geocaulon lividum* | JN965572 | JN966322 | JN999240 | 58.1780 | -93.6370 | 21-Jul-2009 | B.Bennett | BIO |
| 09PROBE-05589 | *Geocaulon lividum* | JN965573 | JN966323 | JN999239 | 58.7470 | -94.1150 | 29-Jul-2009 | M.Kuzmina | BIO |
| 09PROBE-05044 | *Myriophyllum sibiricum* | JN965688 |  | JN999339 | 58.7220 | -93.4280 | 18-Jul-2009 | B.Bennett | BIO |
| 09PROBE-05070 | *Myriophyllum sibiricum *** | JN965689 | JN966386 | JN999336 | 58.3350 | -93.0180 | 19-Jul-2009 | B.Bennett | BIO |
| 09PROBE-05473 | *Myriophyllum sibiricum *** | JN965690 | JN966387 | JN999334 | 58.7050 | -94.0520 | 26-Jul-2009 | M.Kuzmina | BIO |
| 09PROBE-05490 | *Myriophyllum sibiricum* | JN965691 |  | JN999335 | 58.7870 | -93.7200 | 25-Jul-2009 | M.Kuzmina | BIO |
| 09PROBE-05886 | *Myriophyllum sibiricum* | JN965687 |  | JN999340 | 59.0670 | -94.8000 | 15-Jul-1973 | K.L.Johnson | MMMN |
| 09PROBE-05066 | *Chrysosplenium tetrandrum* | JN965449 | JN966245 | JN999120 | 58.3350 | -93.0180 | 19-Jul-2009 | B.Bennett | BIO |
| 09PROBE-05094 | *Mitella nuda* | JN965678 | JN966378 | JN999326 | 58.6290 | -93.7980 | 19-Jul-2009 | K.Johnson, M.Kuzmina | BIO |
| 09PROBE-05113 | *Mitella nuda* | JN965679 |  | JN999325 | 58.6170 | -93.8120 | 19-Jul-2009 | M.Kuzmina, K.Johnson | BIO |
| 09PROBE-05389 | *Saxifraga aizoides* | JN965982 |  | JN999620 | 58.7600 | -94.0880 | 22-Jul-2009 | M.Kuzmina, K.Johnson | BIO |
| 09PROBE-05463 | *Saxifraga aizoides* | JN965983 |  | JN999621 | 58.7310 | -93.7800 | 24-Jul-2009 | M.Kuzmina | BIO |
| 09PROBE-05001 | *Saxifraga cespitosa *** | JN965984 | JN966660 | JN999623 | 58.7690 | -93.8620 | 18-Jul-2009 | K.Johnson, M.Kuzmina | BIO |
| 09PROBE-05071 | *Saxifraga cespitosa *** | JN965985 | JN966661 | JN999622 | 58.3350 | -93.0180 | 19-Jul-2009 | B.Bennett | BIO |
| 09PROBE-05388 | *Saxifraga oppositifolia* | JN965986 |  | JN999624 | 58.7600 | -94.0880 | 22-Jul-2009 | M.Kuzmina, K.Johnson | BIO |
| 09PROBE-05020 | *Saxifraga tricuspidata *** | JN965987 | JN966662 | JN999627 | 58.7710 | -93.8510 | 18-Jul-2009 | K.Johnson, M.Kuzmina | BIO |
| 09PROBE-05104 | *Saxifraga tricuspidata *** | JN965988 | JN966663 | JN999625 | 57.3510 | -93.5230 | 19-Jul-2009 | S.Ponomarenko | BIO |
| 09PROBE-05336 | *Saxifraga tricuspidata *** | JN965989 | JN966664 | JN999626 | 58.7890 | -94.2270 | 21-Jul-2009 | M.Kuzmina | BIO |
| 09PROBE-05025 | *Astragalus alpinus* | JN965275 |  | JN998953 | 58.7390 | -94.1120 | 18-Jul-2009 | K.Johnson, M.Kuzmina | BIO |
| 09PROBE-05036 | *Astragalus alpinus* | JN965276 | JN966120 | JN998954 | 58.7390 | -94.1120 | 18-Jul-2009 | K.Johnson, M.Kuzmina | BIO |
| 09PROBE-05267 | *Astragalus alpinus* | JN965277 | JN966121 | JN998955 | 58.7340 | -94.1120 | 21-Jul-2009 | M.Kuzmina, K.Johnson | BIO |
| 09PROBE-05019 | *Astragalus eucosmus* | JN965278 | JN966122 | JN998959 | 58.7690 | -93.8620 | 18-Jul-2009 | K.Johnson, M.Kuzmina | BIO |
| 09PROBE-05612 | *Astragalus eucosmus* | JN965279 | JN966123 | JN998956 | 58.7180 | -94.1240 | 29-Jul-2009 | M.Kuzmina | BIO |
| 09PROBE-05624 | *Astragalus eucosmus* | JN965280 | JN966124 | JN998958 | 58.7580 | -94.0680 | 29-Jul-2009 | M.Kuzmina | BIO |
| 09PROBE-05627 | *Astragalus eucosmus* | JN965281 |  | JN998957 | 58.7610 | -94.0870 | 30-Jul-2009 | M.Kuzmina | BIO |
| 09PROBE-05037 | *Hedysarum boreale ssp mackenziei* | JN965574 | JN966324 | JN999242 | 58.7390 | -94.1120 | 18-Jul-2009 | K.Johnson, M.Kuzmina | BIO |
| 09PROBE-05229 | *Hedysarum boreale ssp mackenziei* | JN965575 | JN966325 | JN999244 | 58.7710 | -93.8430 | 20-Jul-2009 | M.Kuzmina, K.Johnson | BIO |
| 09PROBE-05334 | *Hedysarum boreale ssp mackenziei* | JN965576 | JN966326 | JN999243 | 58.7890 | -94.2270 | 21-Jul-2009 | M.Kuzmina | BIO |
| 09PROBE-05299 | *Oxytropis varians* | JN965698 | JN966393 | JN999349 | 58.6260 | -94.2300 | 21-Jul-2009 | M.Kuzmina, K.Johnson | BIO |
| 09PROBE-05493 | *Oxytropis varians* | JN965699 | JN966394 | JN999347 | 58.7800 | -94.1950 | 27-Jul-2009 | M.Kuzmina | BIO |
| 09PROBE-05598 | *Oxytropis varians* | JN965700 | JN966395 | JN999348 | 58.7470 | -94.1150 | 29-Jul-2009 | M.Kuzmina | BIO |
| 09PROBE-05302 | *Alnus viridis ssp crispa *** | JN965217 | JN966067 |  | 58.6260 | -94.2300 | 21-Jul-2009 | M.Kuzmina, K.Johnson | BIO |
| 09PROBE-05010 | *Betula glandulosa* | JN965295 | JN966137 | JN998976 | 58.7690 | -93.8620 | 18-Jul-2009 | K.Johnson, M.Kuzmina | BIO |
| 09PROBE-05118 | *Betula glandulosa* | JN965296 | JN966138 | JN998977 | 58.6330 | -93.7900 | 19-Jul-2009 | K.Johnson, M.Kuzmina | BIO |
| 09PROBE-05279 | *Betula glandulosa* |  | JN966139 | JN998975 | 58.7340 | -94.1120 | 21-Jul-2009 | M.Kuzmina, K.Johnson | BIO |
| 09PROBE-05527 | *Betula glandulosa* | JN965297 | JN966140 | JN998978 | 58.6260 | -94.2300 | 27-Jul-2009 | M.Kuzmina | BIO |
| 09PROBE-05905 | *Betula pumila* |  | JN966141 | JN998979 | 57.8510 | -92.7580 | 21-Jul-2003 | E.Punter, M.Piercey-Normore | WIN |
| 09PROBE-05301 | *Myrica gale* | JN965684 | JN966383 | JN999333 | 58.6260 | -94.2300 | 21-Jul-2009 | M.Kuzmina, K.Johnson | BIO |
| 09PROBE-05462 | *Myrica gale* | JN965685 | JN966384 | JN999331 | 58.7310 | -93.7800 | 24-Jul-2009 | M.Kuzmina | BIO |
| 09PROBE-05526 | *Myrica gale* | JN965686 | JN966385 | JN999332 | 58.6260 | -94.2300 | 27-Jul-2009 | M.Kuzmina | BIO |
| 09PROBE-05316 | *Linum lewisii* | JN965646 | JN966360 | JN999303 | 58.7610 | -94.0370 | 20-Jul-2009 | M.Kuzmina, K.Johnson | BIO |
| 09PROBE-05507 | *Linum lewisii *** | JN965647 | JN966361 | JN999302 | 58.7800 | -94.1950 | 27-Jul-2009 | M.Kuzmina | BIO |
| 09PROBE-05087 | *Populus balsamifera* | JN965756 | JN966448 | JN999403 | 58.6290 | -93.7980 | 19-Jul-2009 | K.Johnson, M.Kuzmina | BIO |
| 09PROBE-05472 | *Populus balsamifera* | JN965757 | JN966449 | JN999402 | 58.7050 | -94.0520 | 26-Jul-2009 | M.Kuzmina | BIO |
| 09PROBE-05585 | *Populus balsamifera* | JN965758 | JN966450 | JN999401 | 58.7470 | -94.1150 | 29-Jul-2009 | M.Kuzmina | BIO |
| 09PROBE-05629 | *Salix alaxensis* | JN965837 | JN966522 | JN999477 | 58.7570 | -94.0740 | 30-Jul-2009 | M.Kuzmina | BIO |
| 09PROBE-05925 | *Salix alaxensis* | JN965836 |  | JN999478 | 58.7240 | -93.8380 | 29-Jul-1993 | G.M.Keleher, E.Punter | WIN |
| 09PROBE-05926 | *Salix alaxensis *** | JN965835 | JN966521 | JN999479 | 58.7040 | -93.8450 | 29-Jul-1993 | G.M.Keleher, E.Punter | WIN |
| 09PROBE-05927 | *Salix alaxensis* | JN965834 | JN966520 | JN999476 | 58.6260 | -94.2290 | 10-Jul-1983 | J.M.Shay | WIN |
| 09PROBE-05524 | *Salix alaxensis var alaxensis *** | JN965838 | JN966523 | JN999480 | 58.6260 | -94.2300 | 27-Jul-2009 | M.Kuzmina | BIO |
| 09PROBE-05633 | *Salix alaxensis var alaxensis* | JN965839 | JN966524 | JN999481 | 58.7050 | -94.0520 | 01-Aug-2009 | Rihard Joos | BIO |
| 09PROBE-05391 | *Salix arbusculoides* | JN965842 | JN966528 | JN999485 | 58.7600 | -94.0880 | 22-Jul-2009 | M.Kuzmina, K.Johnson | BIO |
| 09PROBE-05468 | *Salix arbusculoides* | JN965843 | JN966529 | JN999483 | 58.7050 | -94.0520 | 26-Jul-2009 | M.Kuzmina | BIO |
| 09PROBE-05618 | *Salix arbusculoides* | JN965844 | JN966530 | JN999487 | 58.7180 | -94.1240 | 29-Jul-2009 | M.Kuzmina | BIO |
| 09PROBE-05636 | *Salix arbusculoides* | JN965845 | JN966531 | JN999488 | 58.7050 | -94.0520 | 01-Aug-2009 | Rihard Joos | BIO |
| 09PROBE-05929 | *Salix arbusculoides* |  | JN966527 | JN999484 | 58.7400 | -93.8200 | 14-Jul-1993 | G.M.Keleher | WIN |
| 09PROBE-05930 | *Salix arbusculoides* | JN965841 | JN966526 | JN999482 | 57.8370 | -92.8170 | 20-Jul-2003 | E.Punter, M.Piercey-Normore | WIN |
| 09PROBE-05931 | *Salix arbusculoides *** | JN965840 | JN966525 | JN999486 | 58.7240 | -93.8380 | 29-Jul-1994 | G.M.Keleher, E.Punter | WIN |
| 09PROBE-05095 | *Salix arctophila* | JN965852 | JN966539 | JN999490 | 58.6290 | -93.7980 | 19-Jul-2009 | K.Johnson, M.Kuzmina | BIO |
| 09PROBE-05386 | *Salix arctophila* |  | JN966540 |  | 58.7540 | -93.9140 | 23-Jul-2009 | H.Barron | BIO |
| 09PROBE-05545 | *Salix arctophila* | JN965853 | JN966541 | JN999489 | 58.6920 | -94.1320 | 27-Jul-2009 | M.Kuzmina | BIO |
| L#09PROBE-0423_HOST | *Salix arctophila* |  | JN966538 |  | 58.6350 | -93.7880 | 28-Jul-2009 | E.Noel | BIO |
| L#09PROBE-0620_HOST | *Salix arctophila* | JN965851 | JN966537 | JN999491 | 58.6350 | -93.7880 | 29-Jul-2009 | E.Noel | BIO |
| L#09PROBE-0654_HOST | *Salix arctophila* | JN965850 | JN966536 | JN999492 | 58.6350 | -93.7880 | 29-Jul-2009 | E.Noel | BIO |
| L#09PROBE-0657_HOST | *Salix arctophila* | JN965849 | JN966535 | JN999493 | 58.6350 | -93.7880 | 29-Jul-2009 | E.Noel | BIO |
| L#09PROBE-0659_HOST | *Salix arctophila* | JN965848 | JN966534 | JN999494 | 58.6350 | -93.7880 | 29-Jul-2009 | E.Noel | BIO |
| L#09PROBE-1006_HOST | *Salix arctophila* | JN965847 | JN966533 | JN999495 | 58.6300 | -93.8190 | 26-Jul-2009 | E.Noel | BIO |
| L#09PROBE-1014_HOST | *Salix arctophila* | JN965846 | JN966532 | JN999496 | 58.6300 | -93.8190 | 26-Jul-2009 | E.Noel | BIO |
| 09PROBE-05932 | *Salix athabascensis* | JN965856 |  | JN999498 | 57.0030 | -92.3040 | 02-Jul-1969 | D.Punter | WIN |
| 09PROBE-05933 | *Salix athabascensis *** | JN965855 | JN966543 | JN999497 | 50.9900 | -96.1000 | 05-Jul-1951 | H.J.Scoggan | WIN |
| 09PROBE-05934 | *Salix athabascensis *** | JN965854 | JN966542 |  |  |  | 15-Jun-1972 | J.L.Parker | WIN |
| 09PROBE-05103 | *Salix bebbiana* | JN965858 | JN966545 | JN999503 | 58.6330 | -93.7900 | 19-Jul-2009 | K.Johnson, M.Kuzmina | BIO |
| 09PROBE-05537 | *Salix bebbiana* | JN965859 | JN966546 | JN999500 | 58.6260 | -94.2300 | 27-Jul-2009 | M.Kuzmina | BIO |
| 09PROBE-05538 | *Salix bebbiana* | JN965860 | JN966547 | JN999501 | 58.6260 | -94.2300 | 27-Jul-2009 | M.Kuzmina | BIO |
| 09PROBE-05630 | *Salix bebbiana* | JN965861 | JN966548 | JN999499 | 58.6920 | -94.1320 | 28-Jul-2009 | E.Noel | BIO |
| 09PROBE-05935 | *Salix bebbiana *** | JN965857 | JN966544 | JN999502 | 58.7400 | -93.8200 | 01-Aug-1959 | Canada Dept Forestry | WIN |
| 09PROBE-05634 | *Salix brachycarpa* | JN965870 |  | JN999511 | 58.7050 | -94.0520 | 01-Aug-2009 | Rihard Joos | BIO |
| 09PROBE-05936 | *Salix brachycarpa *** | JN965869 | JN966556 | JN999505 | 58.7500 | -94.1100 | 12-Jul-1983 | J.M.Shay | WIN |
| 09PROBE-05937 | *Salix brachycarpa* | JN965868 |  | JN999504 | 58.7400 | -93.8200 | 14-Jul-1983 | J.M.Shay | WIN |
| 09PROBE-05938 | *Salix brachycarpa *** | JN965867 | JN966555 | JN999513 | 58.7240 | -93.8380 | 29-Jul-1994 | G.M.Keleher, E.Punter | WIN |
| 09PROBE-05939 | *Salix brachycarpa *** |  | JN966554 | JN999512 | 58.3050 | -92.9820 | 18-Jul-2002 | B.A.Ford, et al. | WIN |
| 09PROBE-05940 | *Salix brachycarpa* | JN965866 | JN966553 | JN999509 | 58.3060 | -92.9900 | 18-Jul-2002 | B.A.Ford, et al. | WIN |
| 09PROBE-05941 | *Salix brachycarpa* | JN965865 | JN966552 | JN999510 | 58.3060 | -92.9840 | 18-Jul-2003 | B.A.Ford, et al. | WIN |
| L#09PROBE-0623_HOST | *Salix brachycarpa* | JN965863 | JN966550 | JN999507 | 58.6900 | -93.8500 | 26-Jul-2009 | E.Noel | BIO |
| L#09PROBE-0622_HOST | *Salix brachycarpa* | JN965864 | JN966551 | JN999508 | 58.7850 | -93.7370 | 25-Jul-2009 | E.Noel | BIO |
| L#09PROBE-1013_HOST | *Salix brachycarpa* | JN965862 | JN966549 | JN999506 | 58.6300 | -93.8190 | 26-Jul-2009 | E.Noel | BIO |
| 09PROBE-05011 | *Salix brachycarpa var brachycarpa* | JN965871 | JN966557 | JN999517 | 58.7690 | -93.8620 | 18-Jul-2009 | K.Johnson, M.Kuzmina | BIO |
| 09PROBE-05017 | *Salix brachycarpa var brachycarpa *** | JN965872 | JN966558 | JN999515 | 58.7690 | -93.8620 | 18-Jul-2009 | K.Johnson, M.Kuzmina | BIO |
| 09PROBE-05220 | *Salix brachycarpa var brachycarpa* | JN965873 | JN966559 | JN999514 | 58.7640 | -93.8970 | 20-Jul-2009 | M.Kuzmina, K.Johnson | BIO |
| 09PROBE-05234 | *Salix brachycarpa var brachycarpa *** | JN965874 | JN966560 | JN999516 | 58.7710 | -93.8430 | 20-Jul-2009 | M.Kuzmina, K.Johnson | BIO |
| 09PROBE-05235 | *Salix brachycarpa var brachycarpa* | JN965875 | JN966561 | JN999518 | 58.7710 | -93.8430 | 20-Jul-2009 | M.Kuzmina, K.Johnson | BIO |
| 09PROBE-05012 | *Salix calcicola* | JN965880 | JN966565 | JN999526 | 58.7690 | -93.8620 | 18-Jul-2009 | K.Johnson, M.Kuzmina | BIO |
| 09PROBE-05237 | *Salix calcicola* | JN965881 | JN966566 | JN999522 | 58.7470 | -93.8630 | 20-Jul-2009 | M.Kuzmina, K.Johnson | BIO |
| 09PROBE-05563 | *Salix calcicola* |  | JN966567 | JN999525 | 58.7690 | -93.8560 | 29-Jul-2009 | M.Kuzmina | BIO |
| 09PROBE-05596 | *Salix calcicola* | JN965882 | JN966568 | JN999521 | 58.7470 | -94.1150 | 29-Jul-2009 | M.Kuzmina | BIO |
| 09PROBE-05942 | *Salix calcicola* | JN965879 | JN966564 | JN999520 | 57.8370 | -92.8170 | 20-Jul-2003 | E.Punter, M.Piercey-Normore | WIN |
| 09PROBE-05943 | *Salix calcicola *** | JN965878 | JN966563 | JN999523 | 57.8300 | -92.8080 | 19-Jul-2003 | E.Punter, M.Piercey-Normore | WIN |
| 09PROBE-05944 | *Salix calcicola* | JN965877 | JN966562 | JN999519 | 57.8290 | -92.8020 | 19-Jul-2004 | E.Punter, M.Piercey-Normore | WIN |
| 09PROBE-05945 | *Salix calcicola* | JN965876 |  | JN999524 | 57.8350 | -92.7960 | 23-Jul-2003 | E.Punter, M.Piercey-Normore | WIN |
| 09PROBE-05006 | *Salix candida* | JN965892 | JN966578 | JN999527 | 58.7690 | -93.8620 | 18-Jul-2009 | K.Johnson, M.Kuzmina | BIO |
| 09PROBE-05269 | *Salix candida* | JN965893 | JN966579 | JN999532 | 58.7340 | -94.1120 | 21-Jul-2009 | M.Kuzmina, K.Johnson | BIO |
| 09PROBE-05469 | *Salix candida *** | JN965894 | JN966580 | JN999528 | 58.7050 | -94.0520 | 26-Jul-2009 | M.Kuzmina | BIO |
| 09PROBE-05923 | *Salix candida *** | JN965891 | JN966577 | JN999529 | 58.6550 | -93.1780 | 24-Jul-2002 | B.A.Ford, et al. | WIN |
| 09PROBE-05924 | *Salix candida* | JN965890 | JN966576 | JN999530 | 58.7670 | -93.2510 | 16-Jul-2004 | B.A.Ford, et al. | WIN |
| 09PROBE-05928 | *Salix candida* | JN965889 | JN966575 | JN999531 | 57.8300 | -92.8080 | 19-Jul-2003 | E.Punter, M.Piercey-Normore | WIN |
| L#09PROBE-0429_HOST | *Salix candida* | JN965888 | JN966574 | JN999533 | 58.6260 | -94.2290 | 27-Jul-2009 | E.Noel | BIO |
| L#09PROBE-0430_HOST | *Salix candida* | JN965887 | JN966573 | JN999534 | 58.6260 | -94.2290 | 27-Jul-2009 | E.Noel | BIO |
| L#09PROBE-0435_HOST | *Salix candida* | JN965886 | JN966572 | JN999535 | 58.6920 | -94.1320 | 27-Jul-2009 | E.Noel | BIO |
| L#09PROBE-0436_HOST | *Salix candida* | JN965885 | JN966571 | JN999536 | 58.6260 | -94.2290 | 27-Jul-2009 | E.Noel | BIO |
| L#09PROBE-0437_HOST | *Salix candida* | JN965884 | JN966570 | JN999537 | 58.6920 | -94.1320 | 27-Jul-2009 | E.Noel | BIO |
| L#09PROBE-0613_HOST | *Salix candida* | JN965883 | JN966569 | JN999538 | 58.6320 | -93.7880 | 29-Jul-2009 | E.Noel | BIO |
| 09PROBE-05257 | *Salix fuscescens* | JN965895 | JN966581 | JN999539 | 58.1780 | -93.6370 | 21-Jul-2009 | B.Bennett | BIO |
| 09PROBE-05946 | *Salix glauca* | JN965900 | JN966586 |  | 57.8510 | -92.7580 | 21-Jul-2004 | E.Punter, M.Piercey-Normore | WIN |
| 09PROBE-05947 | *Salix glauca *** | JN965899 | JN966585 | JN999542 | 57.8300 | -92.8080 | 22-Jul-2005 | E.Punter, M.Piercey-Normore | WIN |
| 09PROBE-05948 | *Salix glauca* | JN965898 | JN966584 | JN999541 | 57.8510 | -92.7580 | 21-Jul-2004 | E.Punter, M.Piercey-Normore | WIN |
| 09PROBE-05949 | *Salix glauca* | JN965897 | JN966583 | JN999540 | 58.3830 | -93.2250 | 20-Jul-2002 | B.A.Ford, et al. | WIN |
| 09PROBE-05950 | *Salix glauca *** | JN965896 | JN966582 | JN999543 | 58.7240 | -93.8380 | 29-Jul-1994 | G.M.Keleher, E.Punter | WIN |
| 09PROBE-05093 | *Salix glauca ssp callicarpaea* | JN965901 | JN966587 | JN999547 | 58.6290 | -93.7980 | 19-Jul-2009 | K.Johnson, M.Kuzmina | BIO |
| 09PROBE-05101 | *Salix glauca ssp callicarpaea* | JN965902 | JN966588 | JN999545 | 58.6330 | -93.7900 | 19-Jul-2009 | K.Johnson, M.Kuzmina | BIO |
| 09PROBE-05102 | *Salix glauca ssp callicarpaea* | JN965903 | JN966589 | JN999544 | 58.6330 | -93.7900 | 19-Jul-2009 | K.Johnson, M.Kuzmina | BIO |
| 09PROBE-05509 | *Salix glauca ssp callicarpaea* | JN965904 |  | JN999546 | 58.7800 | -94.1950 | 27-Jul-2009 | M.Kuzmina | BIO |
| 09PROBE-05622 | *Salix glauca ssp callicarpaea* | JN965905 | JN966590 | JN999548 | 58.7180 | -94.1240 | 29-Jul-2009 | M.Kuzmina | BIO |
| 09PROBE-05951 | *Salix herbacea* | JN965907 |  | JN999549 | 60.0000 | -98.1670 | 29-Jul-1950 | H.J.Scoggan | WIN |
| 09PROBE-05952 | *Salix herbacea* | JN965906 |  | JN999550 | 59.3670 | -97.7670 | 10-Aug-1950 | H.J.Scoggan | WIN |
| L#09PROBE-0615_HOST | *Salix lanata* | JN965911 | JN966594 | JN999552 | 58.6350 | -93.7870 | 29-Jul-2009 | E.Noel | BIO |
| L#09PROBE-0618_HOST | *Salix lanata* | JN965910 | JN966593 | JN999555 | 58.6350 | -93.7870 | 29-Jul-2009 | E.Noel | BIO |
| L#09PROBE-0648_HOST | *Salix lanata* | JN965909 | JN966592 | JN999554 | 58.7640 | -93.8950 | 29-Jul-2009 | E.Noel | BIO |
| L#09PROBE-0650_HOST | *Salix lanata* | JN965908 | JN966591 | JN999553 | 58.7690 | -93.8890 | 29-Jul-2009 | E.Noel | BIO |
| 09PROBE-05241 | *Salix myrtillifolia* | JN965918 | JN966600 | JN999558 | 58.1780 | -93.6370 | 21-Jul-2009 | B.Bennett | BIO |
| 09PROBE-05955 | *Salix myrtillifolia *** | JN965917 | JN966599 | JN999563 | 58.7400 | -93.8200 | 01-Jul-1975 | J.Serger | WIN |
| 09PROBE-05956 | *Salix myrtillifolia *** | JN965916 | JN966598 | JN999557 | 57.0030 | -92.3040 | 07-Jul-1969 | D.Punter | WIN |
| 09PROBE-05957 | *Salix myrtillifolia* | JN965915 | JN966597 | JN999562 | 58.6750 | -94.1670 | 04-Jul-1973 | P.Cotterill | WIN |
| 09PROBE-05958 | *Salix myrtillifolia* | JN965914 | JN966596 | JN999561 | 58.7400 | -93.8200 | 04-Aug-1953 | E.Beckett | WIN |
| 09PROBE-05959 | *Salix myrtillifolia* | JN965913 |  | JN999560 | 57.0030 | -92.3040 | 24-Jul-1949 | H.J.Scoggan | WIN |
| 09PROBE-05960 | *Salix myrtillifolia* | JN965912 | JN966595 | JN999559 | 58.7040 | -93.8450 | 29-Jul-1994 | G.M.Keleher, E.Punter | WIN |
| 09PROBE-05261 | *Salix pedicellaris* | JN965922 |  |  | 58.4680 | -93.6500 | 22-Jul-2009 | B.Bennett | BIO |
| 09PROBE-05961 | *Salix pedicellaris* | JN965921 | JN966602 | JN999564 | 57.0030 | -92.3040 | 07-Aug-2007 | D.Punter | WIN |
| 09PROBE-05962 | *Salix pedicellaris* | JN965920 |  | JN999565 | 57.0030 | -92.3040 | 28-Jul-1949 | H.J.Scoggan | WIN |
| 09PROBE-05963 | *Salix pedicellaris* | JN965919 | JN966601 | JN999566 | 57.0030 | -92.3040 | 28-Jul-1950 | H.J.Scoggan | WIN |
| 09PROBE-05549 | *Salix pellita* | JN965923 | JN966603 | JN999568 | 58.6920 | -94.1320 | 27-Jul-2009 | M.Kuzmina | BIO |
| 09PROBE-05631 | *Salix pellita* | JN965924 | JN966604 | JN999567 | 58.7570 | -94.0740 | 30-Jul-2009 | M.Kuzmina | BIO |
| 09PROBE-05005 | *Salix planifolia* | JN965953 | JN966633 | JN999571 | 58.7690 | -93.8620 | 18-Jul-2009 | K.Johnson, M.Kuzmina | BIO |
| 09PROBE-05280 | *Salix planifolia* | JN965954 | JN966634 | JN999570 | 58.6260 | -94.2300 | 21-Jul-2009 | M.Kuzmina, K.Johnson | BIO |
| 09PROBE-05491 | *Salix planifolia* | JN965955 | JN966635 |  | 58.7050 | -94.0520 | 26-Jul-2009 | M.Kuzmina | BIO |
| 09PROBE-05504 | *Salix planifolia* | JN965956 | JN966636 | JN999576 | 58.7800 | -94.1950 | 27-Jul-2009 | M.Kuzmina | BIO |
| 09PROBE-05964 | *Salix planifolia* | JN965952 | JN966632 | JN999577 | 58.4080 | -93.0660 | 08-Jul-2002 | B.A.Ford, et al. | WIN |
| 09PROBE-05965 | *Salix planifolia* | JN965951 | JN966631 | JN999578 | 57.8370 | -92.8170 | 20-Jul-2003 | E.Punter, M.Piercey-Normore | WIN |
| 09PROBE-05966 | *Salix planifolia* | JN965950 | JN966630 | JN999579 | 57.8510 | -92.7580 | 21-Jul-2003 | E.Punter, M.Piercey-Normore | WIN |
| 09PROBE-05967 | *Salix planifolia* | JN965949 | JN966629 | JN999580 | 58.6730 | -93.2560 | 21-Jul-2002 | B.A.Ford, et al. | WIN |
| 09PROBE-05968 | *Salix planifolia* | JN965948 | JN966628 | JN999581 | 57.7920 | -93.3660 | 11-Jul-2004 | B.A.Ford, et al. | WIN |
| 09PROBE-05969 | *Salix planifolia* | JN965947 | JN966627 | JN999582 | 58.4670 | -93.2100 | 19-Jul-2002 | B.A.Ford, et al. | WIN |
| 09PROBE-05970 | *Salix planifolia* | JN965946 | JN966626 | JN999583 | 57.7930 | -93.3840 | 12-Jul-2004 | B.A.Ford, et al. | WIN |
| 09PROBE-05971 | *Salix planifolia* | JN965945 |  | JN999569 | 58.7240 | -93.8380 | 29-Jul-1994 | G.M.Keleher, E.Punter | WIN |
| 09PROBE-05972 | *Salix planifolia* | JN965944 | JN966625 | JN999584 | 58.7720 | -93.8450 | 26-Jul-1995 | G.M.Keleher, E.Punter | WIN |
| 09PROBE-05973 | *Salix planifolia* | JN965943 | JN966624 | JN999585 | 58.7400 | -93.8200 | 14-Jul-1993 | G.M.Keheler | WIN |
| L#09PROBE-0421_HOST | *Salix planifolia* |  | JN966623 |  | 58.6920 | -94.1320 | 27-Jul-2009 | E.Noel | BIO |
| L#09PROBE-0424_HOST | *Salix planifolia* | JN965942 | JN966622 | JN999589 | 58.6300 | -93.8190 | 28-Jul-2009 | E.Noel | BIO |
| L#09PROBE-0428_HOST | *Salix planifolia* | JN965940 | JN966620 |  | 58.6920 | -94.1320 | 27-Jul-2009 | E.Noel | BIO |
| L#09PROBE-0433_HOST | *Salix planifolia* | JN965939 | JN966619 | JN999591 | 58.6260 | -94.2290 | 27-Jul-2009 | E.Noel | BIO |
| L#09PROBE-0434_HOST | *Salix planifolia* | JN965938 | JN966618 |  | 58.6920 | -94.1320 | 27-Jul-2009 | E.Noel | BIO |
| L#09PROBE-0439_HOST | *Salix planifolia* | JN965937 | JN966617 |  | 58.6920 | -94.1320 | 27-Jul-2009 | E.Noel | BIO |
| L#09PROBE-0440_HOST | *Salix planifolia* | JN965936 | JN966616 |  | 58.6300 | -93.8190 | 28-Jul-2009 | E.Noel | BIO |
| L#09PROBE-0601_HOST | *Salix planifolia* | JN965935 | JN966615 | JN999592 | 58.6300 | -93.8190 | 26-Jul-2009 | E.Noel | BIO |
| L#09PROBE-0612_HOST | *Salix planifolia* | JN965934 | JN966614 | JN999573 | 58.6350 | -93.7880 | 29-Jul-2009 | E.Noel | BIO |
| L#09PROBE-0619_HOST | *Salix planifolia* | JN965933 | JN966613 | JN999574 | 58.6190 | -93.8290 | 29-Jul-2009 | E.Noel | BIO |
| L#09PROBE-0646_HOST | *Salix planifolia* | JN965930 | JN966610 | JN999586 | 58.6180 | -93.8100 | 26-Jul-2009 | E.Noel | BIO |
| L#09PROBE-0651_HOST | *Salix planifolia* | JN965929 | JN966609 |  | 58.6190 | -93.8290 | 29-Jul-2009 | E.Noel | BIO |
| L#09PROBE-0652_HOST | *Salix planifolia* | JN965928 | JN966608 |  | 58.6350 | -93.7880 | 29-Jul-2009 | E.Noel | BIO |
| L#09PROBE-0656_HOST | *Salix planifolia* | JN965927 | JN966607 | JN999587 | 58.6190 | -93.8290 | 29-Jul-2009 | E.Noel | BIO |
| L#09PROBE-1009_HOST | *Salix planifolia* | JN965925 | JN966605 | JN999572 | 58.6300 | -93.8190 | 26-Jul-2009 | E.Noel | BIO |
| L#09PROBE-0427_HOST | *Salix planifolia* | JN965941 | JN966621 | JN999590 | 58.6300 | -93.8190 | 28-Jul-2009 | E.Noel | BIO |
| L#09PROBE-0641_HOST | *Salix planifolia* | JN965932 | JN966612 | JN999575 | 58.6300 | -93.8190 | 28-Jul-2009 | E.Noel | BIO |
| L#09PROBE-0642_HOST | *Salix planifolia* | JN965931 | JN966611 |  | 58.6300 | -93.8190 | 28-Jul-2009 | E.Noel | BIO |
| L#09PROBE-1002_HOST | *Salix planifolia* | JN965926 | JN966606 | JN999588 | 58.6300 | -93.8190 | 26-Jul-2009 | E.Noel | BIO |
| 09PROBE-05080 | *Salix pseudomyrsinites* | JN965957 | JN966637 | JN999593 | 58.6290 | -93.7980 | 19-Jul-2009 | K.Johnson, M.Kuzmina | BIO |
| 09PROBE-05305 | *Salix pseudomyrsinites* | JN965958 | JN966638 | JN999594 | 58.6260 | -94.2300 | 21-Jul-2009 | M.Kuzmina, K.Johnson | BIO |
| 09PROBE-05536 | *Salix pseudomyrsinites* | JN965959 | JN966639 | JN999597 | 58.6260 | -94.2300 | 27-Jul-2009 | M.Kuzmina | BIO |
| 09PROBE-05621 | *Salix pseudomyrsinites* | JN965960 | JN966640 | JN999595 | 58.7180 | -94.1240 | 29-Jul-2009 | M.Kuzmina | BIO |
| 09PROBE-05635 | *Salix pseudomyrsinites* | JN965961 | JN966641 | JN999596 | 58.7050 | -94.0520 | 01-Aug-2009 | Rihard Joos | BIO |
| 09PROBE-05013 | *Salix reticulata* | JN965972 | JN966651 | JN999608 | 58.7690 | -93.8620 | 18-Jul-2009 | K.Johnson, M.Kuzmina | BIO |
| 09PROBE-05974 | *Salix reticulata* | JN965971 | JN966650 | JN999601 | 57.8300 | -92.8080 | 22-Jul-2003 | E.Punter, M.Piercey-Normore | WIN |
| 09PROBE-05975 | *Salix reticulata* | JN965970 | JN966649 | JN999605 | 58.7670 | -93.2510 | 16-Jul-2004 | B.A.Ford, et al. | WIN |
| 09PROBE-05976 | *Salix reticulata* | JN965969 | JN966648 | JN999606 | 58.7500 | -94.1100 | 13-Jul-1983 | J.M.Shay | WIN |
| 09PROBE-05977 | *Salix reticulata* | JN965968 |  | JN999598 | 58.7400 | -93.8200 | 07-Jul-1988 | L.M.Ross | WIN |
| 09PROBE-05978 | *Salix reticulata *** | JN965967 | JN966647 | JN999599 | 58.7330 | -93.8080 | 26-Jul-1994 | G.M.Keleher, E.Punter | WIN |
| 09PROBE-05979 | *Salix reticulata* | JN965966 | JN966646 | JN999600 | 58.4670 | -93.2100 | 19-Jul-2002 | B.A.Ford, et al. | WIN |
| 09PROBE-05980 | *Salix reticulata* | JN965965 | JN966645 | JN999607 | 58.3830 | -93.2250 | 20-Jul-2002 | B.A.Ford, et al. | WIN |
| 09PROBE-05981 | *Salix reticulata* | JN965964 | JN966644 | JN999602 | 58.6660 | -93.1880 | 17-Jul-2002 | B.A.Ford, et al. | WIN |
| 09PROBE-05982 | *Salix reticulata* | JN965963 | JN966643 | JN999603 | 57.8560 | -92.7530 | 21-Jul-2003 | E.Punter, M.Piercey-Normore | WIN |
| 09PROBE-05983 | *Salix reticulata *** | JN965962 | JN966642 | JN999604 | 57.7930 | -93.3840 | 12-Jul-2004 | B.A.Ford, et al. | WIN |
| 09PROBE-05632 | *Salix richardsonii* | JN965973 | JN966652 | JN999609 | 58.7370 | -93.8190 | 01-Aug-2009 | M.Kuzmina | BIO |
| 09PROBE-05984 | *Salix serissima* | JN965974 |  | JN999610 | 57.0030 | -92.3040 | 20-Jul-1949 | H.J.Scoggan | WIN |
| 09PROBE-05063 | *Salix vestita* | JN965978 | JN966656 | JN999614 | 58.3350 | -93.0180 | 19-Jul-2009 | B.Bennett | BIO |
| 09PROBE-05079 | *Salix vestita* | JN965979 | JN966657 | JN999613 | 58.6290 | -93.7980 | 19-Jul-2009 | K.Johnson, M.Kuzmina | BIO |
| 09PROBE-05470 | *Salix vestita* | JN965980 | JN966658 | JN999611 | 58.7050 | -94.0520 | 26-Jul-2009 | M.Kuzmina | BIO |
| 09PROBE-05546 | *Salix vestita* | JN965981 | JN966659 | JN999612 | 58.6920 | -94.1320 | 27-Jul-2009 | M.Kuzmina | BIO |
| 09PROBE-05985 | *Salix vestita* | JN965977 |  | JN999619 | 58.7740 | -94.1670 | 25-Aug-1973 | K.L.Johnson | WIN |
| 09PROBE-05986 | *Salix vestita* |  | JN966655 | JN999618 | 57.0020 | -92.3060 | 25-Jul-1970 | D.Punter | WIN |
| 09PROBE-05987 | *Salix vestita* | JN965976 | JN966654 | JN999617 | 58.7400 | -93.8200 | 04-Jul-1973 | P.Cotterill | WIN |
| 09PROBE-05988 | *Salix vestita *** | JN965975 | JN966653 | JN999616 | 58.6290 | -93.7980 | 09-Jul-1968 | J.M.Walker | WIN |
| 09PROBE-05016 | *Viola adunca *** | JN966059 | JN966732 | JN999694 | 57.3510 | -93.5240 | 18-Jul-2009 | B.Bennett | BIO |
| 09PROBE-05045 | *Viola renifolia *** | JN966060 | JN966733 | JN999696 | 58.6340 | -93.7860 | 19-Jul-2009 | M.Kuzmina, K.Johnson | BIO |
| 09PROBE-05214 | *Viola renifolia *** | JN966061 | JN966734 | JN999697 | 58.7640 | -93.8970 | 20-Jul-2009 | M.Kuzmina, K.Johnson | BIO |
| 09PROBE-05253 | *Viola renifolia* | JN966062 |  | JN999695 | 58.1780 | -93.6370 | 21-Jul-2009 | B.Bennett | BIO |
| 09PROBE-05009 | *Shepherdia canadensis* | JN965990 | JN966665 | JN999629 | 58.7690 | -93.8620 | 18-Jul-2009 | K.Johnson, M.Kuzmina | BIO |
| 09PROBE-05053 | *Shepherdia canadensis* | JN965991 | JN966666 | JN999628 | 58.6330 | -93.7900 | 19-Jul-2009 | K.Johnson, M.Kuzmina | BIO |
| 09PROBE-05105 | *Shepherdia canadensis* | JN965992 | JN966667 | JN999630 | 57.3510 | -93.5230 | 19-Jul-2009 | S.Ponomarenko | BIO |
| 09PROBE-05362 | *Comarum palustre* | JN965451 | JN966247 | JN999123 | 58.7890 | -94.2270 | 21-Jul-2009 | M.Kuzmina | BIO |
| 09PROBE-05015 | *Dryas octopetala* | JN965495 | JN966272 | JN999166 | 58.7690 | -93.8620 | 18-Jul-2009 | K.Johnson, M.Kuzmina | BIO |
| 09PROBE-05354 | *Dryas octopetala* | JN965496 | JN966273 | JN999165 | 58.7890 | -94.2270 | 21-Jul-2009 | M.Kuzmina | BIO |
| 09PROBE-05991 | *Fragaria virginiana ssp glauca* | JN965552 | JN966305 | JN999221 | 49.7670 | -97.1670 | 29-May-1995 | S.Morgan, et al. | WIN |
| 09PROBE-05003 | *Fragaria virginiana ssp virginiana* | JN965554 | JN966307 | JN999222 | 58.7690 | -93.8620 | 18-Jul-2009 | K.Johnson, M.Kuzmina | BIO |
| 09PROBE-05086 | *Fragaria virginiana ssp virginiana* | JN965555 | JN966308 | JN999224 | 58.6290 | -93.7980 | 19-Jul-2009 | K.Johnson, M.Kuzmina | BIO |
| 09PROBE-05990 | *Fragaria virginiana ssp virginiana* | JN965553 | JN966306 | JN999223 | 57.8300 | -92.8080 | 19-Jul-2003 | E.Punter, M.Piercey-Normore | WIN |
| 09PROBE-05586 | *Potentilla sp.* | JN965762 | JN966454 | JN999407 | 58.7470 | -94.1150 | 29-Jul-2009 | M.Kuzmina | BIO |
| 09PROBE-05224 | *Potentilla anserina ssp egedii* | JN965763 | JN966455 | JN999408 | 58.7710 | -93.8430 | 20-Jul-2009 | M.Kuzmina, K.Johnson | BIO |
| 09PROBE-05291 | *Potentilla anserina ssp egedii* | JN965764 | JN966456 |  | 58.6260 | -94.2300 | 21-Jul-2009 | M.Kuzmina, K.Johnson | BIO |
| 09PROBE-05497 | *Potentilla anserina ssp egedii* | JN965765 | JN966457 | JN999409 | 58.7800 | -94.1950 | 27-Jul-2009 | M.Kuzmina | BIO |
| 09PROBE-05557 | *Potentilla anserina ssp egedii* | JN965766 | JN966458 | JN999410 | 58.7690 | -93.8560 | 29-Jul-2009 | M.Kuzmina | BIO |
| 09PROBE-05202 | *Potentilla bimundorum* |  | JN966459 |  | 58.7700 | -94.1720 | 20-Jul-2009 | B.Bennett | BIO |
| 09PROBE-05320 | *Potentilla bipinnatifida* | JN965768 | JN966461 | JN999412 | 58.7610 | -94.0370 | 20-Jul-2009 | M.Kuzmina, K.Johnson | BIO |
| 09PROBE-05039 | *Potentilla nivea* | JN965769 | JN966462 | JN999414 | 58.7500 | -94.1100 | 18-Jul-2009 | K.Johnson, M.Kuzmina | BIO |
| 09PROBE-05277 | *Potentilla nivea* | JN965770 | JN966463 | JN999415 | 58.7340 | -94.1120 | 21-Jul-2009 | M.Kuzmina, K.Johnson | BIO |
| 09PROBE-05329 | *Potentilla nivea* | JN965771 | JN966464 | JN999413 | 58.7890 | -94.2270 | 21-Jul-2009 | M.Kuzmina | BIO |
| 09PROBE-05495 | *Potentilla nivea* | JN965772 | JN966465 | JN999416 | 58.7800 | -94.1950 | 27-Jul-2009 | M.Kuzmina | BIO |
| 09PROBE-05201 | *Potentilla pensylvanica ** | JN965767 | JN966460 | JN999411 | 58.7700 | -94.1720 | 20-Jul-2009 | B.Bennett | BIO |
| 09PROBE-05359 | *Potentilla pensylvanica* | JN965773 | JN966466 | JN999419 | 58.7890 | -94.2270 | 21-Jul-2009 | M.Kuzmina | BIO |
| 09PROBE-05385 | *Potentilla pensylvanica* | JN965774 | JN966467 | JN999417 | 58.6630 | -94.1670 | 22-Jul-2009 | H.Barron | BIO |
| 09PROBE-05478 | *Potentilla pensylvanica* | JN965775 | JN966468 | JN999418 | 58.7050 | -94.0520 | 26-Jul-2009 | M.Kuzmina | BIO |
| 09PROBE-05319 | *Potentilla pulchella* | JN965776 | JN966469 | JN999420 | 58.7610 | -94.0370 | 20-Jul-2009 | M.Kuzmina, K.Johnson | BIO |
| 09PROBE-05358 | *Potentilla pulchella* | JN965777 | JN966470 | JN999421 | 58.7890 | -94.2270 | 21-Jul-2009 | M.Kuzmina | BIO |
| 09PROBE-05496 | *Potentilla pulchella* | JN965778 | JN966471 | JN999422 | 58.7800 | -94.1950 | 27-Jul-2009 | M.Kuzmina | BIO |
| 09PROBE-05610 | *Rosa acicularis *** | JN965822 | JN966509 | JN999466 | 58.7180 | -94.1240 | 29-Jul-2009 | M.Kuzmina | BIO |
| 09PROBE-05035 | *Rubus acaulis* | JN965823 | JN966510 | JN999469 | 58.7390 | -94.1120 | 18-Jul-2009 | K.Johnson, M.Kuzmina | BIO |
| 09PROBE-05069 | *Rubus acaulis* | JN965824 | JN966511 | JN999468 | 58.3350 | -93.0180 | 19-Jul-2009 | B.Bennett | BIO |
| 09PROBE-05325 | *Rubus acaulis* | JN965825 | JN966512 | JN999467 | 58.7890 | -94.2270 | 21-Jul-2009 | M.Kuzmina | BIO |
| 09PROBE-05232 | *Rubus chamaemorus* | JN965826 | JN966513 | JN999470 | 58.7710 | -93.8430 | 20-Jul-2009 | M.Kuzmina, K.Johnson | BIO |
| 09PROBE-05458 | *Rubus chamaemorus* | JN965827 | JN966514 | JN999471 | 58.7340 | -93.8010 | 24-Jul-2009 | M.Kuzmina | BIO |
| 09PROBE-05615 | *Rubus idaeus* | JN965828 | JN966515 |  | 58.7180 | -94.1240 | 29-Jul-2009 | M.Kuzmina | BIO |
| 09PROBE-05556 | *Urtica dioica* |  | JN966727 | JN999689 | 58.7690 | -93.8560 | 29-Jul-2009 | M.Kuzmina | BIO |
| 09PROBE-05077 | *Ribes hudsonianum* | JN965813 | JN966500 | JN999457 | 58.6290 | -93.7980 | 19-Jul-2009 | K.Johnson, M.Kuzmina | BIO |
| 09PROBE-05245 | *Ribes hudsonianum* | JN965814 | JN966501 | JN999458 | 58.1780 | -93.6370 | 21-Jul-2009 | B.Bennett | BIO |
| 09PROBE-05021 | *Ribes oxyacanthoides* | JN965815 | JN966502 | JN999462 | 58.7710 | -93.8510 | 18-Jul-2009 | K.Johnson, M.Kuzmina | BIO |
| 09PROBE-05117 | *Ribes oxyacanthoides* | JN965816 | JN966503 | JN999460 | 58.6160 | -93.8080 | 19-Jul-2009 | K.Johnson, M.Kuzmina | BIO |
| 09PROBE-05219 | *Ribes oxyacanthoides* | JN965817 | JN966504 | JN999461 | 58.7640 | -93.8970 | 20-Jul-2009 | M.Kuzmina, K.Johnson | BIO |
| 09PROBE-05324 | *Ribes oxyacanthoides* | JN965818 | JN966505 | JN999459 | 58.7890 | -94.2270 | 21-Jul-2009 | M.Kuzmina | BIO |
| 09PROBE-05048 | *Ribes triste* | JN965819 | JN966506 | JN999464 | 58.6330 | -93.7900 | 19-Jul-2009 | K.Johnson, M.Kuzmina | BIO |
| 09PROBE-05244 | *Ribes triste* | JN965820 | JN966507 | JN999463 | 58.1780 | -93.6370 | 21-Jul-2009 | B.Bennett | BIO |
| 09PROBE-05088 | *Arabidopsis petraea ssp umbrosa* | JN965252 | JN966099 | JN998930 | 58.6290 | -93.7980 | 19-Jul-2009 | K.Johnson, M.Kuzmina | BIO |
| 09PROBE-05457 | *Arabidopsis petraea ssp umbrosa* | JN965253 | JN966100 | JN998931 | 58.7720 | -94.1630 | 24-Jul-2009 | M.Kuzmina | BIO |
| 09PROBE-05499 | *Arabidopsis petraea ssp umbrosa* | JN965254 |  | JN998932 | 58.7800 | -94.1950 | 27-Jul-2009 | M.Kuzmina | BIO |
| 09PROBE-05916 | *Arabidopsis petraea ssp umbrosa* | JN965251 | JN966098 | JN998929 | 58.7670 | -93.2510 | 16-Jul-2004 | B.A.Ford, et al. | WIN |
| 09PROBE-05917 | *Arabidopsis petraea ssp umbrosa* | JN965250 | JN966097 | JN998928 | 58.6740 | -93.1910 | 22-Jul-2002 | B.A.Ford, et al. | WIN |
| 09PROBE-05914 | *Arabis alpina* | JN965256 | JN966102 | JN998933 | 58.6300 | -93.8190 | 09-Aug-1972 | J.Hunt | WIN |
| 09PROBE-05915 | *Arabis alpina* | JN965255 | JN966101 | JN998934 | 58.6300 | -93.8190 | 22-Jun-1973 | M.Austin | WIN |
| 09PROBE-05380 | *Barbarea orthoceras* | JN965286 | JN966130 |  | 58.6630 | -94.1670 | 22-Jul-2009 | H.Barron | BIO |
| 09PROBE-05623 | *Barbarea orthoceras* | JN965287 |  | JN998967 | 58.6630 | -94.1670 | 29-Jul-2009 | M.Kuzmina | BIO |
| 09PROBE-05447 | *Capsella bursa-pastoris* | JN965329 | JN966165 | JN999009 | 58.7720 | -94.1630 | 24-Jul-2009 | M.Kuzmina | BIO |
| 09PROBE-05287 | *Cardamine pratensis* | JN965330 |  | JN999010 | 58.6260 | -94.2300 | 21-Jul-2009 | M.Kuzmina, K.Johnson | BIO |
| 09PROBE-05581 | *Cardamine pratensis* |  | JN966166 | JN999011 | 58.7450 | -93.8950 | 29-Jul-2009 | M.Kuzmina | BIO |
| 09PROBE-05073 | *Draba alpina* | JN965471 | JN966257 | JN999143 | 58.3350 | -93.0180 | 19-Jul-2009 | B.Bennett | BIO |
| 09PROBE-05212 | *Draba alpina* | JN965472 | JN966258 | JN999141 | 58.7470 | -93.8630 | 20-Jul-2009 | M.Kuzmina, K.Johnson | BIO |
| 09PROBE-05909 | *Draba alpina* | JN965470 |  | JN999142 | 58.7960 | -94.2040 |  | J.C.Ritchi | WIN |
| 09PROBE-05262 | *Draba aurea* | JN965474 | JN966259 | JN999145 | 58.7340 | -94.1120 | 21-Jul-2009 | M.Kuzmina, K.Johnson | BIO |
| 09PROBE-05910 | *Draba aurea* | JN965473 |  | JN999144 | 58.6300 | -93.8190 | 09-Aug-1972 | J.Hunt | WIN |
| 09PROBE-05002 | *Draba glabella* | JN965477 | JN966262 | JN999147 | 58.7690 | -93.8620 | 18-Jul-2009 | K.Johnson, M.Kuzmina | BIO |
| 09PROBE-05321 | *Draba glabella* | JN965478 | JN966263 | JN999151 | 58.7890 | -94.2270 | 21-Jul-2009 | M.Kuzmina | BIO |
| 09PROBE-05337 | *Draba glabella* | JN965479 | JN966264 | JN999146 | 58.7890 | -94.2270 | 21-Jul-2009 | M.Kuzmina | BIO |
| 09PROBE-05483 | *Draba glabella* | JN965480 |  | JN999149 | 58.7920 | -93.7510 | 25-Jul-2009 | M.Kuzmina | BIO |
| 09PROBE-05485 | *Draba glabella* | JN965481 | JN966265 | JN999148 | 58.7920 | -93.7510 | 25-Jul-2009 | M.Kuzmina | BIO |
| 09PROBE-05566 | *Draba glabella* | JN965482 | JN966266 | JN999152 | 58.7690 | -93.8560 | 29-Jul-2009 | M.Kuzmina | BIO |
| 09PROBE-05919 | *Draba glabella* | JN965476 | JN966261 |  | 58.7670 | -93.2510 | 16-Jul-2004 | B.A.Ford, et al. | WIN |
| 09PROBE-05920 | *Draba glabella* | JN965475 | JN966260 | JN999150 | 58.4080 | -93.0610 | 18-Jul-2002 | B.A.Ford, et al. | WIN |
| 09PROBE-05921 | *Draba incana* | JN965484 |  | JN999155 | 58.7510 | -93.8230 | 29-Jul-1994 | G.M.Keleher, E.Punter | WIN |
| 09PROBE-05922 | *Draba incana* | JN965483 |  | JN999154 | 58.7050 | -94.0520 | 01-Sep-1970 | R.E.Longton | WIN |
| 09PROBE-05042 | *Draba nemorosa* | JN965487 | JN966268 | JN999157 | 58.7500 | -94.1100 | 18-Jul-2009 | K.Johnson, M.Kuzmina | BIO |
| 09PROBE-05115 | *Draba nemorosa* | JN965488 | JN966269 | JN999158 | 58.6170 | -93.8120 | 19-Jul-2009 | M.Kuzmina, K.Johnson | BIO |
| 09PROBE-05907 | *Draba nemorosa* | JN965486 | JN966267 | JN999156 | 58.7400 | -93.8200 | 16-Jul-1953 | E.Beckett | WIN |
| 09PROBE-05908 | *Draba nemorosa* | JN965485 |  | JN999159 | 56.3530 | -94.7140 |  | W.B.Schofield | WIN |
| 09PROBE-05911 | *Draba nivalis* | JN965491 | JN966270 | JN999161 | 58.7670 | -93.2510 | 16-Jul-2004 | B.A.Ford, et al. | WIN |
| 09PROBE-05912 | *Draba nivalis* | JN965490 |  | JN999162 | 58.6660 | -93.1880 | 17-Jul-2002 | B.A.Ford, et al. | WIN |
| 09PROBE-05913 | *Draba nivalis* | JN965489 |  | JN999160 | 58.6660 | -93.1880 | 17-Jul-2003 | B.A.Ford, et al. | WIN |
| 09PROBE-05511 | *Erysimum sp.* | JN965542 |  | JN999211 | 58.7700 | -94.1720 | 27-Jul-2009 | M.Kuzmina | BIO |
| 09PROBE-05392 | *Lesquerella arctica* | JN965626 | JN966346 | JN999288 | 58.7600 | -94.0880 | 22-Jul-2009 | M.Kuzmina, K.Johnson | BIO |
| 09PROBE-05381 | *Rorippa palustris* | JN965821 | JN966508 | JN999465 | 58.6630 | -94.1670 | 22-Jul-2009 | H.Barron | BIO |
| 09PROBE-05595 | *Thlaspi arvense* | JN966038 | JN966711 | JN999672 | 58.7470 | -94.1150 | 29-Jul-2009 | M.Kuzmina | BIO |
| 09PROBE-05569 | *Chamerion angustifolium* | JN965438 | JN966233 |  | 58.7690 | -93.8560 | 29-Jul-2009 | M.Kuzmina | BIO |
| 09PROBE-05570 | *Chamerion angustifolium* | JN965439 | JN966234 |  | 58.7690 | -93.8560 | 29-Jul-2009 | M.Kuzmina | BIO |
| 09PROBE-05107 | *Chamerion angustifolium* | JN965434 | JN966229 | JN999108 | 58.6160 | -93.8080 | 19-Jul-2009 | K.Johnson, M.Kuzmina | BIO |
| 09PROBE-05233 | *Chamerion angustifolium* | JN965435 | JN966230 | JN999106 | 58.7710 | -93.8430 | 20-Jul-2009 | M.Kuzmina, K.Johnson | BIO |
| 09PROBE-05288 | *Chamerion angustifolium* | JN965436 | JN966231 | JN999107 | 58.6260 | -94.2300 | 21-Jul-2009 | M.Kuzmina, K.Johnson | BIO |
| 09PROBE-05326 | *Chamerion angustifolium* | JN965437 | JN966232 | JN999109 | 58.7890 | -94.2270 | 21-Jul-2009 | M.Kuzmina | BIO |
| 09PROBE-05625 | *Chamerion angustifolium *** | JN965433 | JN966228 | JN999110 | 58.7580 | -94.0680 | 29-Jul-2009 | Maria Kuzmina | BIO |
| 09PROBE-05453 | *Chamerion latifolium *** | JN965442 | JN966236 | JN999113 | 58.7720 | -94.1630 | 24-Jul-2009 | M.Kuzmina | BIO |
| 09PROBE-05606 | *Chamerion latifolium* | JN965443 | JN966237 |  | 58.7180 | -94.1240 | 29-Jul-2009 | M.Kuzmina | BIO |
| 09PROBE-05993 | *Chamerion latifolium* | JN965441 |  | JN999111 | 58.7330 | -93.8000 | 28-Jul-1994 | G.M.Keleher, E.Punter | WIN |
| 09PROBE-05994 | *Chamerion latifolium* | JN965440 | JN966235 | JN999112 | 58.1280 | -92.8560 | 11-Aug-1983 | M.Zbigniewicz | WIN |
| 09PROBE-05996 | *Epilobium sp.* | JN965518 |  | JN999186 | 57.0030 | -92.3040 | 28-Jul-1949 | H.J.Scoggan | WIN |
| 09PROBE-06000 | *Epilobium sp.* | JN965517 |  | JN999185 | 58.7560 | -94.0790 | 08-Aug-1956 | J.C.Ritchi | WIN |
| 09PROBE-05121 | *Epilobium palustre *** |  | JN966286 | JN999193 | 58.7500 | -94.1100 | 21-Jul-1984 | M.Zbigniewicz | WIN |
| 09PROBE-05122 | *Epilobium palustre* | JN965524 |  | JN999192 | 58.7500 | -94.1100 | 21-Jul-1985 | M.Zbigniewicz | WIN |
| 09PROBE-05528 | *Epilobium palustre *** | JN965525 | JN966287 | JN999191 | 58.6260 | -94.2300 | 27-Jul-2009 | M.Kuzmina | BIO |
| 09PROBE-05992 | *Epilobium palustre* | JN965523 |  | JN999194 | 57.0030 | -92.3040 | 19-Jul-1970 | D.Punter | WIN |
| 09PROBE-05995 | *Epilobium palustre* | JN965522 | JN966285 | JN999189 | 57.8560 | -92.7530 | 21-Jul-2003 | E.Punter, M.Piercey-Normore | WIN |
| 09PROBE-05997 | *Epilobium palustre* | JN965521 |  | JN999190 | 58.6260 | -94.2290 | 03-Aug-1983 | M.Zbigniewicz | WIN |
| 09PROBE-05998 | *Epilobium palustre* | JN965520 |  | JN999187 | 58.7400 | -93.8200 | 02-Aug-1983 | M.Zbigniewicz | WIN |
| 09PROBE-05999 | *Epilobium palustre* | JN965519 |  | JN999188 | 57.0030 | -92.3040 | 26-Jul-1949 | H.J.Scoggan | WIN |
| 09PROBE-05209 | *Andromeda polifolia* | JN965229 | JN966077 | JN998916 | 58.7470 | -93.8630 | 20-Jul-2009 | M.Kuzmina, K.Johnson | BIO |
| 09PROBE-05353 | *Andromeda polifolia *** | JN965230 | JN966078 | JN998914 | 58.7890 | -94.2270 | 21-Jul-2009 | M.Kuzmina | BIO |
| 09PROBE-05567 | *Andromeda polifolia* | JN965231 | JN966079 | JN998915 | 58.7690 | -93.8560 | 29-Jul-2009 | M.Kuzmina | BIO |
| 09PROBE-05076 | *Arctostaphylos uva-ursi* | JN965264 | JN966109 | JN998943 | 58.6290 | -93.7980 | 19-Jul-2009 | K.Johnson, M.Kuzmina | BIO |
| 09PROBE-05599 | *Arctostaphylos uva-ursi* | JN965265 | JN966110 | JN998942 | 58.7470 | -94.1150 | 29-Jul-2009 | M.Kuzmina | BIO |
| 09PROBE-05023 | *Arctous alpina* | JN965266 | JN966111 | JN998945 | 58.7390 | -94.1120 | 18-Jul-2009 | K.Johnson, M.Kuzmina | BIO |
| 09PROBE-05371 | *Arctous alpina* | JN965267 | JN966112 | JN998944 | 57.3510 | -93.5230 | 21-Jul-2009 | S.Ponomarenko | BIO |
| 09PROBE-05008 | *Arctous ruber* | JN965268 | JN966113 | JN998948 | 58.7690 | -93.8620 | 18-Jul-2009 | K.Johnson, M.Kuzmina | BIO |
| 09PROBE-05061 | *Arctous ruber* | JN965269 | JN966114 | JN998946 | 58.6330 | -93.7900 | 19-Jul-2009 | K.Johnson, M.Kuzmina | BIO |
| 09PROBE-05364 | *Arctous ruber* | JN965270 | JN966115 | JN998947 | 58.7890 | -94.2270 | 21-Jul-2009 | M.Kuzmina | BIO |
| 09PROBE-05024 | *Empetrum nigrum* | JN965513 | JN966281 | JN999182 | 58.7390 | -94.1120 | 18-Jul-2009 | K.Johnson, M.Kuzmina | BIO |
| 09PROBE-05056 | *Empetrum nigrum* | JN965514 | JN966282 | JN999184 | 58.6330 | -93.7900 | 19-Jul-2009 | K.Johnson, M.Kuzmina | BIO |
| 09PROBE-05562 | *Empetrum nigrum* | JN965515 | JN966283 | JN999181 | 58.7690 | -93.8560 | 29-Jul-2009 | M.Kuzmina | BIO |
| 09PROBE-05588 | *Empetrum nigrum* | JN965516 | JN966284 | JN999183 | 58.7470 | -94.1150 | 29-Jul-2009 | M.Kuzmina | BIO |
| 09PROBE-05085 | *Kalmia polifolia* | JN965613 | JN966341 | JN999278 | 58.6290 | -93.7980 | 19-Jul-2009 | K.Johnson, M.Kuzmina | BIO |
| 09PROBE-05255 | *Kalmia polifolia* | JN965614 | JN966342 | JN999277 | 58.1780 | -93.6370 | 21-Jul-2009 | B.Bennett | BIO |
| 09PROBE-05309 | *Kalmia polifolia* | JN965615 | JN966343 | JN999276 | 58.6920 | -94.1320 | 21-Jul-2009 | K.Johnson | BIO |
| 09PROBE-05084 | *Kalmia procumbens* | JN965616 | JN966344 | JN999279 | 58.6290 | -93.7980 | 19-Jul-2009 | K.Johnson, M.Kuzmina | BIO |
| 09PROBE-05060 | *Moneses uniflora* | JN965681 | JN966380 | JN999329 | 58.6330 | -93.7900 | 19-Jul-2009 | K.Johnson, M.Kuzmina | BIO |
| 09PROBE-05271 | *Moneses uniflora* | JN965682 | JN966381 | JN999328 | 58.7340 | -94.1120 | 21-Jul-2009 | M.Kuzmina, K.Johnson | BIO |
| 09PROBE-05539 | *Moneses uniflora *** | JN965683 | JN966382 | JN999330 | 58.6920 | -94.1320 | 27-Jul-2009 | M.Kuzmina | BIO |
| 09PROBE-05097 | *Orthilia secunda* | JN965693 | JN966388 | JN999342 | 58.6290 | -93.7980 | 19-Jul-2009 | K.Johnson, M.Kuzmina | BIO |
| 09PROBE-05263 | *Orthilia secunda* | JN965694 | JN966389 | JN999344 | 58.7340 | -94.1120 | 21-Jul-2009 | M.Kuzmina, K.Johnson | BIO |
| 09PROBE-05592 | *Orthilia secunda *** | JN965695 | JN966390 | JN999343 | 58.7470 | -94.1150 | 29-Jul-2009 | M.Kuzmina | BIO |
| 09PROBE-05307 | *Oxycoccus microcarpus *** | JN965696 | JN966391 | JN999345 | 58.6920 | -94.1320 | 21-Jul-2009 | K.Johnson | BIO |
| 09PROBE-05348 | *Oxycoccus microcarpus* | JN965697 | JN966392 | JN999346 | 58.7890 | -94.2270 | 21-Jul-2009 | M.Kuzmina | BIO |
| 09PROBE-05014 | *Pyrola grandiflora* | JN965790 | JN966483 | JN999438 | 58.7690 | -93.8620 | 18-Jul-2009 | K.Johnson, M.Kuzmina | BIO |
| 09PROBE-05058 | *Pyrola grandiflora* | JN965791 | JN966484 | JN999436 | 58.6330 | -93.7900 | 19-Jul-2009 | K.Johnson, M.Kuzmina | BIO |
| 09PROBE-05106 | *Pyrola grandiflora* | JN965792 | JN966485 | JN999439 | 57.3510 | -93.5230 | 19-Jul-2009 | S.Ponomarenko | BIO |
| 09PROBE-05290 | *Pyrola grandiflora* | JN965793 | JN966486 | JN999437 | 58.6260 | -94.2300 | 21-Jul-2009 | M.Kuzmina, K.Johnson | BIO |
| 09PROBE-05328 | *Pyrola grandiflora* | JN965794 | JN966487 | JN999435 | 58.7890 | -94.2270 | 21-Jul-2009 | M.Kuzmina | BIO |
| 09PROBE-05051 | *Rhododendron groenlandicum* | JN965806 | JN966493 |  | 58.6330 | -93.7900 | 19-Jul-2009 | K.Johnson, M.Kuzmina | BIO |
| 09PROBE-05466 | *Rhododendron groenlandicum* | JN965807 | JN966494 | JN999452 | 58.7310 | -93.7800 | 24-Jul-2009 | M.Kuzmina | BIO |
| 09PROBE-05211 | *Rhododendron lapponicum* | JN965808 | JN966495 |  | 58.7470 | -93.8630 | 20-Jul-2009 | M.Kuzmina, K.Johnson | BIO |
| 09PROBE-05352 | *Rhododendron lapponicum* | JN965809 | JN966496 | JN999454 | 58.7890 | -94.2270 | 21-Jul-2009 | M.Kuzmina | BIO |
| 09PROBE-05565 | *Rhododendron lapponicum* | JN965810 | JN966497 | JN999453 | 58.7690 | -93.8560 | 29-Jul-2009 | M.Kuzmina | BIO |
| 09PROBE-05057 | *Rhododendron tomentosum ssp decumbens* | JN965811 | JN966498 | JN999456 | 58.6330 | -93.7900 | 19-Jul-2009 | K.Johnson, M.Kuzmina | BIO |
| 09PROBE-05474 | *Rhododendron tomentosum ssp decumbens* | JN965812 | JN966499 | JN999455 | 58.7050 | -94.0520 | 26-Jul-2009 | M.Kuzmina | BIO |
| 09PROBE-05018 | *Vaccinium uliginosum* | JN966056 | JN966729 | JN999691 | 58.7690 | -93.8620 | 18-Jul-2009 | K.Johnson, M.Kuzmina | BIO |
| 09PROBE-05047 | *Vaccinium vitis-idaea* | JN966057 | JN966730 | JN999692 | 58.6330 | -93.7900 | 19-Jul-2009 | K.Johnson, M.Kuzmina | BIO |
| 09PROBE-05355 | *Vaccinium vitis-idaea* | JN966058 | JN966731 | JN999693 | 58.7890 | -94.2270 | 21-Jul-2009 | M.Kuzmina | BIO |
| 09PROBE-05040 | *Androsace septentrionalis* | JN965232 | JN966080 | JN998918 | 58.7500 | -94.1100 | 18-Jul-2009 | K.Johnson, M.Kuzmina | BIO |
| 09PROBE-05067 | *Androsace septentrionalis* | JN965233 | JN966081 | JN998919 | 58.3350 | -93.0180 | 19-Jul-2009 | B.Bennett | BIO |
| 09PROBE-05116 | *Androsace septentrionalis* | JN965234 | JN966082 | JN998917 | 58.6170 | -93.8120 | 19-Jul-2009 | M.Kuzmina, K.Johnson | BIO |
| 09PROBE-05004 | *Primula egaliksensis* | JN965779 | JN966472 | JN999423 | 58.7690 | -93.8620 | 18-Jul-2009 | K.Johnson, M.Kuzmina | BIO |
| 09PROBE-05339 | *Primula egaliksensis *** |  | JN966474 |  | 58.7890 | -94.2270 | 21-Jul-2009 | M.Kuzmina | BIO |
| 09PROBE-05026 | *Primula stricta* | JN965781 |  | JN999427 | 58.7390 | -94.1120 | 18-Jul-2009 | K.Johnson, M.Kuzmina | BIO |
| 09PROBE-05031 | *Primula stricta ** | JN965780 | JN966473 | JN999424 | 58.7390 | -94.1120 | 18-Jul-2009 | K.Johnson, M.Kuzmina | BIO |
| 09PROBE-05289 | *Primula stricta* | JN965782 | JN966475 | JN999428 | 58.6260 | -94.2300 | 21-Jul-2009 | M.Kuzmina, K.Johnson | BIO |
| 09PROBE-05583 | *Primula stricta* | JN965783 | JN966476 | JN999426 | 58.7450 | -93.8950 | 29-Jul-2009 | M.Kuzmina | BIO |
| 09PROBE-05133 | *Mertensia maritima* | JN965671 |  |  | 58.6890 | -93.1590 | 23-Jul-2002 | B.A.Ford, et al. | WIN |
| 09PROBE-05135 | *Mertensia paniculata* | JN965672 | JN966371 |  | 57.0030 | -92.3040 | 07-Aug-2007 | D.Punter | WIN |
| 09PROBE-05137 | *Mertensia paniculata* |  | JN966370 |  | 57.0030 | -92.3040 | 29-Jun-1969 | D.Punter | WIN |
| 09PROBE-05123 | *Gentianella amarella* | JN965560 |  | JN999225 | 58.6300 | -93.8190 | 01-Aug-1971 | R.E.Longton | WIN |
| 09PROBE-05124 | *Gentianella amarella *** | JN965559 | JN966312 | JN999228 | 58.6300 | -93.8190 | 09-Aug-1972 | J.Hunt | WIN |
| 09PROBE-05125 | *Gentianella amarella* | JN965558 | JN966311 | JN999227 | 58.7400 | -93.8200 | 01-Aug-1973 | K.L.Johnson | WIN |
| 09PROBE-05126 | *Gentianella amarella *** | JN965557 | JN966310 | JN999226 | 58.6300 | -93.8190 | 13-Aug-1971 | R.E.Longton | WIN |
| 09PROBE-05127 | *Gentianella propinqua *** | JN965565 | JN966315 | JN999229 | 58.6300 | -93.8190 | 09-Aug-1972 | J.Hunt | WIN |
| 09PROBE-05128 | *Gentianella propinqua* | JN965564 | JN966314 | JN999238 | 57.8300 | -92.8080 | 19-Jul-2003 | E.Punter, M.Piercey-Normore | WIN |
| 09PROBE-05129 | *Gentianella propinqua* | JN965563 |  | JN999237 | 57.0030 | -92.3040 | 24-Jul-1970 | D.Punter | WIN |
| 09PROBE-05130 | *Gentianella propinqua* | JN965562 | JN966313 | JN999236 | 58.7320 | -93.8220 | 30-Jul-1994 | G.M.Keleher, E.Punter | WIN |
| 09PROBE-05131 | *Gentianella propinqua* | JN965561 |  | JN999235 | 58.7400 | -93.8200 | 09-Jul-1959 | J.M.Walker | WIN |
| 09PROBE-05448 | *Gentianella propinqua* | JN965566 | JN966316 | JN999231 | 58.7720 | -94.1630 | 24-Jul-2009 | M.Kuzmina | BIO |
| 09PROBE-05514 | *Gentianella propinqua *** | JN965567 | JN966317 | JN999232 | 58.7700 | -94.1720 | 27-Jul-2009 | M.Kuzmina | BIO |
| 09PROBE-05529 | *Gentianella propinqua *** | JN965568 | JN966318 | JN999230 | 58.6260 | -94.2300 | 27-Jul-2009 | M.Kuzmina | BIO |
| 09PROBE-05590 | *Gentianella propinqua *** | JN965569 | JN966319 | JN999233 | 58.7470 | -94.1150 | 29-Jul-2009 | M.Kuzmina | BIO |
| 09PROBE-05614 | *Gentianella propinqua* | JN965570 | JN966320 | JN999234 | 58.7180 | -94.1240 | 29-Jul-2009 | M.Kuzmina | BIO |
| 09PROBE-05593 | *Galeopsis tetrahit* | JN965556 | JN966309 |  | 58.7470 | -94.1150 | 29-Jul-2009 | M.Kuzmina | BIO |
| 09PROBE-05554 | *Mentha canadensis* | JN965668 | JN966367 | JN999317 | 58.7690 | -93.8560 | 29-Jul-2009 | M.Kuzmina | BIO |
| 09PROBE-05594 | *Stachis palustris* | JN966013 | JN966688 |  | 58.7470 | -94.1150 | 29-Jul-2009 | M.Kuzmina | BIO |
| 09PROBE-05256 | *Pinguicula villosa* | JN965726 | JN966419 |  | 58.1780 | -93.6370 | 21-Jul-2009 | B.Bennett | BIO |
| 09PROBE-05311 | *Pinguicula villosa* | JN965727 | JN966420 | JN999373 | 58.6920 | -94.1320 | 21-Jul-2009 | K.Johnson | BIO |
| 09PROBE-05210 | *Pinguicula vulgaris* | JN965728 | JN966421 | JN999375 | 58.7470 | -93.8630 | 20-Jul-2009 | M.Kuzmina, K.Johnson | BIO |
| 09PROBE-05273 | *Pinguicula vulgaris* | JN965729 | JN966422 | JN999374 | 58.7340 | -94.1120 | 21-Jul-2009 | M.Kuzmina, K.Johnson | BIO |
| 09PROBE-05344 | *Pinguicula vulgaris* | JN965730 | JN966423 | JN999377 | 58.7890 | -94.2270 | 21-Jul-2009 | M.Kuzmina | BIO |
| 09PROBE-05597 | *Pinguicula vulgaris* | JN965731 | JN966424 | JN999376 | 58.7470 | -94.1150 | 29-Jul-2009 | M.Kuzmina | BIO |
| 09PROBE-05258 | *Utricularia intermedia* | JN966055 | JN966728 | JN999690 | 58.1780 | -93.6370 | 21-Jul-2009 | B.Bennett | BIO |
| 09PROBE-05213 | *Bartsia alpina* | JN965288 | JN966131 | JN998969 | 58.7470 | -93.8630 | 20-Jul-2009 | M.Kuzmina, K.Johnson | BIO |
| 09PROBE-05251 | *Bartsia alpina* | JN965289 | JN966132 | JN998968 | 58.1780 | -93.6370 | 21-Jul-2009 | B.Bennett | BIO |
| 09PROBE-05265 | *Bartsia alpina* | JN965290 | JN966133 | JN998970 | 58.7340 | -94.1120 | 21-Jul-2009 | M.Kuzmina, K.Johnson | BIO |
| 09PROBE-05108 | *Castilleja raupii* | JN965425 | JN966219 | JN999100 | 58.6160 | -93.8080 | 19-Jul-2009 | K.Johnson, M.Kuzmina | BIO |
| 09PROBE-05323 | *Castilleja raupii* | JN965426 | JN966220 | JN999101 | 58.7890 | -94.2270 | 21-Jul-2009 | M.Kuzmina | BIO |
| 09PROBE-05508 | *Castilleja raupii* | JN965427 | JN966221 |  | 58.7800 | -94.1950 | 27-Jul-2009 | M.Kuzmina | BIO |
| 09PROBE-05520 | *Euphrasia subarctica *** | JN965543 | JN966296 | JN999213 | 58.6260 | -94.2300 | 27-Jul-2009 | M.Kuzmina | BIO |
| 09PROBE-05601 | *Euphrasia subarctica *** | JN965544 | JN966297 | JN999212 | 58.7470 | -94.1150 | 29-Jul-2009 | M.Kuzmina | BIO |
| 09PROBE-05351 | *Pedicularis albolabiata* | JN965710 | JN966404 | JN999357 | 58.7890 | -94.2270 | 21-Jul-2009 | M.Kuzmina | BIO |
| 09PROBE-05046 | *Pedicularis flammea* | JN965711 | JN966405 | JN999358 | 58.6340 | -93.7860 | 19-Jul-2009 | M.Kuzmina, K.Johnson | BIO |
| 09PROBE-05205 | *Pedicularis flammea* | JN965712 | JN966406 | JN999360 | 58.7470 | -93.8630 | 20-Jul-2009 | M.Kuzmina, K.Johnson | BIO |
| 09PROBE-05350 | *Pedicularis flammea* | JN965713 | JN966407 | JN999361 | 58.7890 | -94.2270 | 21-Jul-2009 | M.Kuzmina | BIO |
| 09PROBE-05560 | *Pedicularis flammea* | JN965714 | JN966408 | JN999359 | 58.7690 | -93.8560 | 29-Jul-2009 | M.Kuzmina | BIO |
| 09PROBE-05274 | *Pedicularis groenlandica* | JN965715 | JN966409 | JN999363 | 58.7340 | -94.1120 | 21-Jul-2009 | M.Kuzmina, K.Johnson | BIO |
| 09PROBE-05605 | *Pedicularis groenlandica* | JN965716 | JN966410 | JN999362 | 58.7180 | -94.1240 | 29-Jul-2009 | M.Kuzmina | BIO |
| 09PROBE-05055 | *Pedicularis labradorica* | JN965717 | JN966411 | JN999365 | 58.6330 | -93.7900 | 19-Jul-2009 | K.Johnson, M.Kuzmina | BIO |
| 09PROBE-05314 | *Pedicularis labradorica* | JN965718 | JN966412 | JN999364 | 58.6920 | -94.1320 | 21-Jul-2009 | K.Johnson | BIO |
| 09PROBE-05109 | *Pedicularis lapponica* | JN965719 | JN966413 | JN999368 | 58.6160 | -93.8080 | 19-Jul-2009 | K.Johnson, M.Kuzmina | BIO |
| 09PROBE-05119 | *Pedicularis lapponica* | JN965720 | JN966414 | JN999367 | 58.7310 | -93.7800 | 20-Jul-2009 | D.Porco | BIO |
| 09PROBE-05252 | *Pedicularis lapponica* | JN965721 | JN966415 | JN999366 | 58.1780 | -93.6370 | 21-Jul-2009 | B.Bennett | BIO |
| 09PROBE-05506 | *Rhinanthus minus ssp borealis* | JN965804 |  | JN999450 | 58.7800 | -94.1950 | 27-Jul-2009 | M.Kuzmina | BIO |
| 09PROBE-05519 | *Rhinanthus minus ssp borealis* | JN965805 | JN966492 | JN999451 | 58.6260 | -94.2300 | 27-Jul-2009 | M.Kuzmina | BIO |
| 09PROBE-05513 | *Callitriche hermaphroditica *** | JN965323 | JN966161 | JN999003 | 58.7800 | -94.1950 | 27-Jul-2009 | M.Kuzmina | BIO |
| 09PROBE-05043 | *Hippuris tetraphylla* | JN965582 | JN966332 | JN999250 | 58.7220 | -93.4280 | 18-Jul-2009 | B.Bennett | BIO |
| 09PROBE-05065 | *Hippuris tetraphylla* | JN965583 | JN966333 | JN999252 | 58.3350 | -93.0180 | 19-Jul-2009 | B.Bennett | BIO |
| 09PROBE-05292 | *Hippuris tetraphylla* | JN965584 | JN966334 | JN999251 | 58.6260 | -94.2300 | 21-Jul-2009 | M.Kuzmina, K.Johnson | BIO |
| 09PROBE-05518 | *Hippuris tetraphylla* | JN965585 |  | JN999253 | 58.6260 | -94.2300 | 27-Jul-2009 | M.Kuzmina | BIO |
| 09PROBE-05387 | *Hippuris vulgaris* | JN965586 | JN966335 | JN999255 | 57.3510 | -93.5230 | 23-Jul-2009 | S.Ponomarenko | BIO |
| 09PROBE-05467 | *Hippuris vulgaris* | JN965587 | JN966336 | JN999254 | 58.7310 | -93.7800 | 24-Jul-2009 | M.Kuzmina | BIO |
| 09PROBE-05441 | *Linaria vulgaris* | JN965643 | JN966356 |  | 58.7720 | -94.1630 | 24-Jul-2009 | M.Kuzmina | BIO |
| 09PROBE-05512 | *Plantago major* | JN965732 | JN966425 | JN999378 | 58.7800 | -94.1950 | 27-Jul-2009 | M.Kuzmina | BIO |
| 09PROBE-05331 | *Plantago maritima *** | JN965733 | JN966426 | JN999380 | 58.7890 | -94.2270 | 21-Jul-2009 | M.Kuzmina | BIO |
| 09PROBE-05578 | *Cicuta virosa* | JN965450 | JN966246 | JN999121 | 58.7450 | -93.8950 | 29-Jul-2009 | M.Kuzmina | BIO |
| 09PROBE-05114 | *Achillea millefolium ssp borealis* | JN965213 | JN966063 | JN998898 | 58.6170 | -93.8120 | 19-Jul-2009 | M.Kuzmina, K.Johnson | BIO |
| 09PROBE-05297 | *Achillea millefolium ssp borealis* | JN965214 | JN966064 | JN998899 | 58.6260 | -94.2300 | 21-Jul-2009 | M.Kuzmina, K.Johnson | BIO |
| 09PROBE-05607 | *Achillea millefolium ssp borealis* | JN965215 | JN966065 | JN998900 | 58.7180 | -94.1240 | 29-Jul-2009 | M.Kuzmina | BIO |
| 09PROBE-05824 | *Antennaria pulcherrima* | JN965248 | JN966095 |  | 58.7400 | -93.8200 |  |  | MMMN |
| 09PROBE-05218 | *Arctanthemum arcticum* | JN965258 | JN966104 | JN998936 | 58.7640 | -93.8970 | 20-Jul-2009 | M.Kuzmina, K.Johnson | BIO |
| 09PROBE-05332 | *Arctanthemum arcticum* | JN965259 | JN966105 | JN998937 | 58.7890 | -94.2270 | 21-Jul-2009 | M.Kuzmina | BIO |
| 09PROBE-05294 | *Arnica angustifolia ssp angustifolia* | JN965272 | JN966117 | JN998952 | 58.6260 | -94.2300 | 21-Jul-2009 | M.Kuzmina, K.Johnson | BIO |
| 09PROBE-05363 | *Arnica angustifolia ssp angustifolia* | JN965273 | JN966118 | JN998950 | 58.7890 | -94.2270 | 21-Jul-2009 | M.Kuzmina | BIO |
| 09PROBE-05505 | *Arnica angustifolia ssp angustifolia* | JN965274 | JN966119 | JN998951 | 58.7800 | -94.1950 | 27-Jul-2009 | M.Kuzmina | BIO |
| 09PROBE-05442 | *Packera paupercula* | JN965701 | JN966396 | JN999352 | 58.7720 | -94.1630 | 24-Jul-2009 | M.Kuzmina | BIO |
| 09PROBE-05482 | *Packera paupercula* | JN965702 | JN966397 | JN999351 | 58.7920 | -93.7510 | 25-Jul-2009 | M.Kuzmina | BIO |
| 09PROBE-05608 | *Packera paupercula* | JN965703 |  | JN999350 | 58.7180 | -94.1240 | 29-Jul-2009 | M.Kuzmina | BIO |
| 09PROBE-05007 | *Petasites frigidus ssp palmatus* | JN965722 | JN966416 | JN999371 | 58.7690 | -93.8620 | 18-Jul-2009 | K.Johnson, M.Kuzmina | BIO |
| 09PROBE-05075 | *Petasites frigidus ssp palmatus* | JN965723 | JN966417 | JN999370 | 58.6290 | -93.7980 | 19-Jul-2009 | K.Johnson, M.Kuzmina | BIO |
| 09PROBE-05275 | *Petasites frigidus ssp palmatus* |  | JN966418 | JN999369 | 58.7340 | -94.1120 | 21-Jul-2009 | M.Kuzmina, K.Johnson | BIO |
| 09PROBE-05225 | *Solidago multiradiata* | JN966003 | JN966679 | JN999641 | 58.7710 | -93.8430 | 20-Jul-2009 | M.Kuzmina, K.Johnson | BIO |
| 09PROBE-05443 | *Solidago multiradiata* | JN966004 |  | JN999640 | 58.7720 | -94.1630 | 24-Jul-2009 | M.Kuzmina | BIO |
| 09PROBE-05510 | *Solidago multiradiata* | JN966005 | JN966680 | JN999644 | 58.7800 | -94.1950 | 27-Jul-2009 | M.Kuzmina | BIO |
| 09PROBE-05584 | *Solidago multiradiata* | JN966006 | JN966681 | JN999643 | 58.7450 | -93.8950 | 29-Jul-2009 | M.Kuzmina | BIO |
| 09PROBE-05616 | *Solidago multiradiata* | JN966007 | JN966682 | JN999639 | 58.7180 | -94.1240 | 29-Jul-2009 | M.Kuzmina | BIO |
| 09PROBE-05617 | *Solidago multiradiata* | JN966008 | JN966683 | JN999642 | 58.7180 | -94.1240 | 29-Jul-2009 | M.Kuzmina | BIO |
| 09PROBE-05619 | *Solidago multiradiata* | JN966009 | JN966684 | JN999645 | 58.7470 | -94.1150 | 29-Jul-2009 | M.Kuzmina | BIO |
| 09PROBE-05530 | *Symphyotrichum boreale* | JN966028 | JN966701 | JN999662 | 58.6260 | -94.2300 | 27-Jul-2009 | M.Kuzmina | BIO |
| 09PROBE-05609 | *Symphyotrichum boreale* |  | JN966702 | JN999663 | 58.7180 | -94.1240 | 29-Jul-2009 | M.Kuzmina | BIO |
| 09PROBE-05098 | *Taraxacum ceratophorum* | JN966029 | JN966703 | JN999666 | 58.6290 | -93.7980 | 19-Jul-2009 | K.Johnson, M.Kuzmina | BIO |
| 09PROBE-05227 | *Taraxacum ceratophorum* | JN966030 | JN966704 | JN999665 | 58.7710 | -93.8430 | 20-Jul-2009 | M.Kuzmina, K.Johnson | BIO |
| 09PROBE-05501 | *Taraxacum ceratophorum* | JN966031 | JN966705 | JN999664 | 58.7800 | -94.1950 | 27-Jul-2009 | M.Kuzmina | BIO |
| 09PROBE-05203 | *Taraxacum lacerum* | JN966032 | JN966706 | JN999667 | 58.7700 | -94.1720 | 20-Jul-2009 | B.Bennett | BIO |
| 09PROBE-05295 | *Taraxacum lacerum* | JN966033 | JN966707 | JN999668 | 58.6260 | -94.2300 | 21-Jul-2009 | M.Kuzmina, K.Johnson | BIO |
| 09PROBE-05503 | *Taraxacum lacerum* | JN966034 | JN966708 |  | 58.7800 | -94.1950 | 27-Jul-2009 | M.Kuzmina | BIO |
| 09PROBE-05223 | *Tephroseris palustris* | JN966035 | JN966709 | JN999669 | 58.7710 | -93.8430 | 20-Jul-2009 | M.Kuzmina, K.Johnson | BIO |
| 09PROBE-05480 | *Tephroseris palustris* | JN966036 | JN966710 | JN999670 | 58.7920 | -93.7510 | 25-Jul-2009 | M.Kuzmina | BIO |
| 09PROBE-05120 | *Tripleurospermum maritimum ssp phaeocephalum* | JN966050 | JN966722 | JN999685 | 58.7700 | -94.1720 | 20-Jul-2009 | B.Bennett | BIO |
| 09PROBE-05384 | *Tripleurospermum maritimum ssp phaeocephalum* | JN966051 | JN966723 | JN999684 | 58.6630 | -94.1670 | 22-Jul-2009 | H.Barron | BIO |
| 09PROBE-05894 | *Campanula uniflora* | JN965328 | JN966164 | JN999006 | 59.0670 | -94.8000 | 15-Jul-1973 | K.L.Johnson | MMMN |
| 09PROBE-05895 | *Campanula uniflora* | JN965327 | JN966163 | JN999007 | 59.0670 | -94.8000 | 15-Jul-1973 | K.L.Johnson | MMMN |
| 09PROBE-05896 | *Campanula uniflora* | JN965326 |  | JN999008 | 58.7640 | -94.2250 | 07-Jul-1942 | A.Simpson | MMMN |
| 09PROBE-05379 | *Menyanthes trifoliata* | JN965669 | JN966368 | JN999318 | 58.6630 | -94.1670 | 22-Jul-2009 | H.Barron | BIO |
| 09PROBE-05459 | *Menyanthes trifoliata* | JN965670 | JN966369 |  | 58.7340 | -93.8010 | 24-Jul-2009 | M.Kuzmina | BIO |
| 09PROBE-05247 | *Linnaea borealis* | JN965645 | JN966359 |  | 58.1780 | -93.6370 | 21-Jul-2009 | B.Bennett | BIO |
| 09PROBE-05892 | *Linnaea borealis* | JN965644 | JN966358 | JN999299 | 57.0030 | -92.3040 | 19-Jul-1979 | K.L.Johnson | MMMN |
| 09PROBE-05893 | *Linnaea borealis* |  | JN966357 | JN999300 | 57.0030 | -92.3040 | 27-Jul-1974 | B.Briscoe | MMMN |

* Identification corrected using barcods as supplemetary information

** matK barcode amplified with matK_390f/matK_1326r primers
