# Supplementary material for: Identification of the vascular plants of Churchill, Manitoba, using a DNA barcode library
Source: BMC Ecol. 2012 Nov 28;12:25. doi: 10.1186/1472-6785-12-25 (PMC3538695; doi:10.1186/1472-6785-12-25)
Supplement: Additional file 2 — Pseudogenes amplified with matK_390f/matK_1326r. [file 1472-6785-12-25-S2.docx]

**Appendix 2B. The *mat*K pseudogenes amplified by matK_390f/matK_1326r primers**

>09PROBE-05812_Limnorchis aquilonis

CATTTGTTGCGATTTATTTTCCACGAATATCATAAGAGTTTCATTACTTCAAAGAAAGACATTGCGTGGATGGGGCTTTTCAAAAAGAAAGAAAAGATTTTTTTGGTTCTGACATAATTCTTCTGTATATGAATGCGAATATATATTCCTGTTTCTTCGCAAACAGTCTTCTTATTTACGATCAACATCTTTTGAAGTCTTTCTTGAGCGAACTGGAAAAAGAGAAGATTTTAGAGTAATGTATTGTAATTCTTTTCAGAGGATTCTATGGTTCCTCAAAGAACCTTTCTTATGTTCGATATCAAGGATAATAAATTATGGCTTCAAAGGTTCTGATGAAGAGATGTTCATTTTTGGCAATTTTATTTTCACTTTTGGTCTCAACCTTCTAGGATCCCATAGAAAGGAATTACCCAACTCTTCCTTTCCTTCTCTTTTCTGGGGTCTTTTTTAAGTGTACTAAAAAAGCAAGAGCTTTGGTAGTAAGAAAGAAAATGCTGGAGAATTCATTTTGAAGAAAGACTCTGACTCAGAAATGAGATACCATAGCCCCAGTGATTTCTCTTATTGGAGCATTGTCAAAAGCTCAATTTTGTACCCATCCCATTAGTCAACCAATCTGGACCGATTTCTCGGATTCTGATATTCTTGATCGATTTTGTCGGATCGGATATGTAGAAATCTTTGTCGTTATCACAGTGGATCCTCAAAACAGGTTTTGTATCGTATAA

>09PROBE-05813_Limnorchis hyperborea

CCTTCTTTGCATTTGTTGCGATTTATTTTCCACGAATATCATAAGAGTTTCATTACTTCAAAGAAAGACATTGCGTGGATGGGGCTTTTCAAAAAGAAAGAAAAGATTTTTTTGGTTCTGACATAATTCTTCTGTATATGAATGCGAATATATATTCCTGTTTCTTCGCAAACAGTCTTCTTATTTACGATCAACATCTTTTGAAGTCTTTCTTGAGCGAACTGGAAAAAGAGAAGATTTTAGAGTAATGTATTGTAATTCTTTTCAGAGGATTCTATGGTTCCTCAAAGAACCTTTCTTATGTTCGATATCAAGGATAATAAATTATGGCTTCAAAGGTTCTGATGAAGAGATGTTCATTTTTGGCAATTTTATTTTCACTTTTGGTCTCAACCTTCTAGGATCCCATAGAAAGGAATTACCCAACTCTTCCTTTCCTTCTCTTTTCTGGGGTCTTTTTTAAGTGTACTAAAAAAGCAAGAGCTTTGGTAGTAAGAAAGAAAATGCTGGAGAATTCATTTTGAAGAAAGACTCTGACTCAGAAATGAGATACCATAGCCCCAGTGATTTCTCTTATTGGAGCATTGTCAAAAGCTCAATTTTGTACCCATCCCATTAGTCAACCAATCTGGACCGATTTCTCGGATTCTGATATTCTTGATCGATTTTGTCGGATCGGATATGTAGAAATCTTTGTCGTTATCACAGTGGATCCTCAAAACAGGTTTTGTATCGTATAA

>09PROBE-05814_Limnorchis hyperborea CCTTCTTTGCATTTGTTGCGATTTATTTTCCACGAATATCATAAGAGTTTCATTACTTCAAAGAAAGACATTGCGTGGATGGGGCTTTTCAAAAAGAAAGAAAAGATTTTTTTGGTTCTGACATAATTCTTCTGTATATGAATGCGAATATATATTCCTGTTTCTTCGCAAACAGTCTTCTTATTTACGATCAACATCTTTTGAAGTCTTTCTTGAGCGAACTGGAAAAAGAGAAGATTTTAGAGTAATGTATTGTAATTCTTTTCAGAGGATTCTATGGTTCCTCAAAGAACCTTTCTTATGTTCGATATCAAGGATAATAAATTATGGCTTCAAAGGTTCTGATGAAGAGATGTTCATTTTTGGCAATTTTATTTTCACTTTTGGTCTCAACCTTCTAGGATCCCATAGAAAGGAATTACCCAACTCTTCCTTTCCTTCTCTTTTCTGGGGTCTTTTTTAAGTGTACTAAAAAAGCAAGAGCTTTGGTAGTAAGAAAGAAAATGCTGGAGAATTCATTTTGAAGAAAGACTCTGACTCAGAAATGAGATACCATAGCCCCAGTGATTTCTCTTATTGGAGCATTGTCAAAAGCTCAATTTTGTACCCATCCCATTAGTCAACCAATCTGGACCGATTTCTCGGATTCTGATATTCTTGATCGATTTTGTCGGATCGGATATGTAGAAATCTTTGTCGTTATCACAGTGGATCCTCAAAACAGGTTTTGTATCGTATAA

>09PROBE-05817_Limnorchis huronensis

CATTTGTTGCGATTTATTTTCCACGAATATCATAAGAGTTTCATTACTTCAAAGAAAGACATTGCGTGGATGGGGCTTTTCAAAAAGAAAGAAAAGATTTTTTTGGTTCTGACATAATTCTTCTGTATATGAATGCGAATATATATTCCTGTTTCTTCGCAAACAGTCTTCTTATTTACGATCAACATCTTTTGAAGTCTTTCTTGAGCGAACTGGAAAAAGAGAAGATTTTAGAGTAATGTATTGTAATTCTTTTCAGAGGATTCTATGGTTCCTCAAAGAACCTTTCTTATGTTCGATATCAAGGATAATAAATTATGGCTTCAAAGGTTCTGATGAAGAGATGTTCATTTTTGGCAATTTTATTTTCACTTTTGGTCTCAACCTTCTAGGATCCCATAGAAAGGAATTACCCAACTCTTCCTTTCCTTCTCTTTTCTGGGGTCTTTTTTAAGTGTACTAAAAAAGCAAGAGCTTTGGTAGTAAGAAAGAAAATGCTGGAGAATTCATTTTGAAGAAAGACTCTGACTCAGAAATGAGATACCATAGCCCCAGTGATTTCTCTTATTGGAGCATTGTCAAAAGCTCAATTTTGTACCCATCCCATTAGTCAACCAATCTGGACCGATTTCTCGGATTCTGATATTCTTGATCGATTTTGTCGGATCGGATATGTAGAAATCTTTGTCGTTATCACAGTGGATCCTCAAAACAGGTTTTGTATCGTATAA

>09PROBE-05822_Amerorchis rotundifolia

ATTTTCCACGAATATCATAATTGAAAGAGTCTCTCATTACTTCAAAGAAAGACATTGCGTGGATGGGGCTTTTCAAAAAGAAAGAAAAGATTTTTTTGGTTCTGACATAATTCTTCTGTATATGAATGCGAATATATATTCCTGTTTCTTCGCAAACAGTCTTCTTATTTACCATCTTTTGATGTCTTTCTTGAGCGAACTGGAAAAAGAGAAGATTTTAGAGTAATGTAATGTATTGTAATTCTTTTCAGAGGATTCTATGGTTCCTCAAAGAACCTTTCTTATGTTCGATATCAAGGAAAATCAATTATGGCTTCAAAGGTTCTGATGAAGATGTTCATTTTTGGCAATTTTCTTTTCACTTTTGGTCTCAACCTTCTAGGATCCCATAGAAAGGAATTACCCAACTCTTCCTTTCCTTCTCTTTTCTGGGGTCTTTTTTAAGTGTACTAAAAAAGCAAGAAGAGCTTTGGTAGTAAGAAAGAAAATGCTGGAGAATTCATTTTGAAGAAAGACTCTGACTCAGAAATGAGATACCATAGCCCCAGTGATTTCTCTTATTGGAGCATTGTCAAAAGCTCAATTTTGTACCCATCCCATTAGTCAACCAATCTGGACCGATTTCTCGGATTCTGATATTCTTGATTTTGTCGGATCGGATATGTAGAAATCTTTGTCGTTATCACAGTGGATCCTCAAAAAAACAGGTTTTGTATCGTCTAA

>09PROBE-05819_Cypripedium passerinum CCATCTGGAAATCTTGGTTCAAAGTGCAAATCCTTCAATGCTGGATCAAAGATGTTCCCGATTGTTTTTCTCATAATTTGAATAGTCTCATTACTTCAAAGTAATCCATTTAGTCTTTTCAAAAAGAAAGAAAAGATTCTTTTTTTTCCCATATATATATTATGTATATATGTATATGAATGCGAATATCTATTCCTGTTTCTTCGTAAACAGTCTTGATCAACATCTTCTGGAATCGAACACATTTCTATGGAAAAATAGAACAAGTAGTGTGTTGTAATTCTTTTCAGAAGATCCTATGGTTCCTTAAAGATCCTTTCATGCATTATGTTCGATATCAAGGAAAAGCAATTCTAAATTAGTAAACCGATCTGGACCGATTTATCGGATATGTAGAAATCTTTGTCGTTATCACAGCGGATCCTCAAAAAAACAGGTTTTGTATCGTATAAAGAAAGTATATACTTCGACTTTCGTGTGCTAGAACTTTGGCTCGTAAACACAAAAGT

>09PROBE-05821_Cypripedium passerinum

TCCCGATTGTTTTTCTCATAATTTGAATAGTCTCATTACTTCAAAGTAATCCATTTAGTCTTTTCAAAAAGAAAGAAAAGATTCTTTTTTTTCCCATATATATATTATGTATATATGTATATGAATGCGAATATCTATTCCTGTTTCTTCGTAAACAGTCTTGATCAACATCTTCTGGAATCGAACACATTTCTATGGAAAAATAGAACAAGTAGTGTGTTGTAATTCTTTTCAGAAGATCCTATGGTTCCTTAAAGATCCTTTCATGCATTATGTTCGATATCAAGGAAAAGCAATTCTAAATTAGTAAACCGATCTGGACCGATTTATCGGATATGTAGAAATCTTTGTCGTTATCACAGCGGATCCTCAAAAAAACAGGTTTTGTATCGTATAAAGAAAGTATATACTTCGACTTTCGTGTGCTAGAACTTTGGCT

>09PROBE-05959_Salix myrtillifolia TATTTCTTTTTTTGCAAAAAAGACTCCAAGATTCTTCTTGTTCTTATATAATTCTCATGTATATGAATACGAGTCCGTTTTCTTTTTTCTTTGTAATCAATCCTTTCATTTCCGATTAACATTTTCTCAGGTCTTTCTTGAGCGAATATATTTTCTATGGAAAAATAGAACATTTTGTAGAAGTCTTTACTAAGGATTGGGGGGACAGCCTATGCTTGCTCAAGGATCCTTTCATACATTATCTTAGATATCAAGGAAAATCCATTTTTGTCTCAAAGGATACGCCTCTTCTGATGAAAAAATGGAAATATTACCTTGTCAATTTATGTCAATGTCATTTTGATGTGTGCTTTCAACCCCCCAGGATCCATATAAACCCATTTTCATTATACAAGCATTCTTTCGCCTTATTAGGTTATCTTTCAAGTTCAAGTGTGCGACTAAACCTTTCAGTGGTACGGAGTCAAATGCTAGAAAATGCATTTCTAATAGATAATATTATGAATAAACTCGATACAACAGTTTCAATTATTCCTTTGATTGGATCATTAGCAAAACTGAAATTTTGTAACGCAGTAGGACATCCCATTAGTAAACCGGCCTGGGCCGATTTTTCGGATTCTGATATTATCGACCGATTTGTCCGTATATGCAGAAATCTTTCTCATTATTATAG
