# Supplementary material for: Identification of the vascular plants of Churchill, Manitoba, using a DNA barcode library
Source: BMC Ecol. 2012 Nov 28;12:25. doi: 10.1186/1472-6785-12-25 (PMC3538695; doi:10.1186/1472-6785-12-25)
Supplement: Additional file 3 — Cases of intraspecific variation. [file 1472-6785-12-25-S3.docx]

**Appendix 3. Intraspecific variation for *mat*K and ITS2.**
